# GRAViTy-V2: a grounded viral taxonomy application

**DOI:** 10.1093/nargab/lqae183

**Published:** 2024-12-18

**Authors:** Richard Mayne, Pakorn Aiewsakun, Dann Turner, Evelien M Adriaenssens, Peter Simmonds

**Affiliations:** Peter Medawar Building for Pathogen Research, Nuffield Department of Medicine, University of Oxford, 3 South Parks Road, OX1 3SY Oxfordshire, UK; Department of Microbiology, Faculty of Science, Mahidol University, 272 Rama VI Road, Thung Phaya Thai, Ratchathewi, Bangkok 10400, Thailand; School of Applied Sciences, University of the West of England, Frenchay Campus, BS16 1QY Bristol, UK; Quadram Institute Bioscience, Rosalind Franklin Rd, NR4 7UQ Norwich, UK; Peter Medawar Building for Pathogen Research, Nuffield Department of Medicine, University of Oxford, 3 South Parks Road, OX1 3SY Oxfordshire, UK

## Abstract

Taxonomic classification of viruses is essential for understanding their evolution. Genomic classification of viruses at higher taxonomic ranks, such as order or phylum, is typically based on alignment and comparison of amino acid sequence motifs in conserved genes. Classification at lower taxonomic ranks, such as genus or species, is usually based on nucleotide sequence identities between genomic sequences. Building on our whole-genome analytical classification framework, we here describe Genome Relationships Applied to Viral Taxonomy Version 2 (GRAViTy-V2), which encompasses a greatly expanded range of features and numerous optimisations, packaged as an application that may be used as a general-purpose virus classification tool. Using 28 datasets derived from the ICTV 2022 taxonomy proposals, GRAViTy-V2 output was compared against human expert-curated classifications used for assignments in the 2023 round of ICTV taxonomy changes. GRAViTy-V2 produced taxonomies equivalent to manually-curated versions down to the family level and in almost all cases, to genus and species levels. The majority of discrepant results arose from errors in coding sequence annotations in INDSC records, or from inclusion of incomplete genome sequences in the analysis. Analysis times ranged from 1-506 min (median 3.59) on datasets with 17-1004 genomes and mean genome length of 3000–1 000 000 bases.

## Introduction

Taxonomic classification of viruses is essential for understanding their evolution, geographical distribution, host interactions and pathogenic mechanisms. Historically, attributes such as virion structure, pathogenicity in their hosts, replication mechanisms and epidemiology have been used to define virus taxa, typically classifying them into order, families, genera and species, analogous to the taxonomy of cellular life forms. However, the recent application of high throughput sequencing technologies to aquatic, terrestrial and gut microbiome samples has revealed an astonishing diversity of viruses infecting prokaryotes and eukaryotes (1–4).

To ensure that virus taxonomy better captures the true diversity of viruses, the International Committee on Taxonomy of Viruses (ICTV), on consultation with the wider virology community, agreed to formally classify viruses known only by their nucleotide sequences (5). More recently, the principles behind an evolutionarily based classification of viruses have been proposed based on genomic relatedness (6), although taxonomic groupings based on these criteria are typically congruent with structural, biological and morphological characteristics of the member viruses. While the recently proposed evolutionarily based framework for classification of viruses (6) is anchored on metrics of genomic relatedness, the resulting taxonomic assignments generally correlate with structural, biological and morphological characteristics of the member viruses.

Where a universal framework for taxonomy of cellular organisms is made possible by sequence comparisons from shared ancestral genetic components (e.g. ribosomal genes), there are no equivalent sets of shared genes shared among all viruses; different groups of viruses may have entirely separate evolutionary origins (7). Homology of hallmark genes may therefore only be used to elucidate taxonomic relationships within individual groups of viruses with a common ancestry (8). Consequently, taxonomic assignments in different virus groups may be based on sequence comparisons or, at deeper taxonomic levels, the detection of protein structural homologies between subjectively-selected gene sets. All of this requires expert curation, expertise in protein structure prediction and effective multiple sequence alignment (MSA) methodologies for often highly divergent virus sequences (9). Consequently, there is no simple, general-purpose approach for investigating virus relationships, even at lower taxonomic levels such as order, family and genus.

A further challenge that frustrates efforts to classify viruses relates to tractability of computation. Since the advent of metagenomic sequencing, the rate of new virus discovery has increased exponentially (10). Viral genomes are comparatively small, but calculating similarity between them based on the results of alignments—once a mainstay through tools such as PASC (11)—quickly becomes infeasible when hundreds of virus genomes are compared.

Standard methods for virus taxonomy assignment typically involve making a MSA, which requires the presence of sequences with detectable similarity across all organisms being analysed. This approach can present a significant challenge, particularly when studying a highly diverse set of viruses; gene or protein sequences that do not show detectable homology among all members of the dataset must be excluded from MSA-based analyses.

As a result, many high-level virus taxonomic classifications are currently based on MSAs of short, conserved genetic sequences (motifs), such as RNA-dependent RNA polymerases (RdRp) in RNA viruses (6). This process usually requires extensive human curation, and involves wasteful exclusion of a substantial portion of genome sequences, even though they could be taxonomically relevant within some specific subgroups.

Alignment-free sequence comparison methods are, conversely, not bound by the assumption of underlying sequence homology and therefore are more amenable to comparison of whole viral genomes that may have vastly different compositions (12). Many alignment-free approaches exist and are in common usage, a popular category of which involve subsetting sequences and calculating their comparative frequencies (*k*-mer methods) (12,13).

In previous work (14,15), we described and evaluated ‘GRAViTy’ (Genome Relationships Applied to Virus Taxonomy), a framework for identifying and classifying viruses, based on analysis of coding complete genomes. GRAViTy generates a single metric, termed the composite Jaccard score (CJS), to indicate the overall degree of similarity between each virus genome’s protein coding region profiles. This is achieved through analysing genomic elements encoded as as protein profile hidden Markov models (PPHMMs). This CJS additionaly takes into consideration PPHMM locations, orders, and orientations, which are collectively compiled as ‘genomic organisational models’ (GOMs). The method is not alignment-free, as MSAs are created during data preprocessing to cluster and normalise the length of amino acid sequences as prerequisites to their being encoded as PPHMMs. GRAViTy does not, however, use MSAs for virus genome comparison and does not need to be supplied with a MSA to function: it therefore has no requirement for all genomes within an analysis to show detectable similarity. This enables the software to make informative comparisons of viruses even when they have drastically different sets of conserved genes.

Initial iterations of the framework were computationally expensive, not packaged as a discrete software and insensitive in specific conditions, such as comparison of very long viral genomes, and computing classifications for datasets with a large proportion of previously unclassified sequences.

In this article we present ‘GRAViTy-V2’, which implements a comprehensively-updated, expanded and optimised framework as a standalone, user-friendly application. This implementation allows one or more virus sequences (new or directly from the published accession numbers of complete genomes) to be compared with viruses classified in the latest release of the ICTV taxonomy. Through computation of a CJS, overall genomic similarity to existing taxa can be quantified, and visualised in the form of heatmaps and dendrograms. The new version further introduces a range of ‘explainability’ features for describing how classifications were arrived at and hence support a wider range of taxonomy tasks.

The use of ‘grounded’ in this article’s title is derived from Computational Grounded Theory, which was used as inspiration for the design of our software and holds that more methodologically rigorous, interpretative approaches to content analysis are derived from a combination of computational pattern recognition and expert human knowledge (16).

We present here a comprehensive evaluation of GRAViTy-V2, in which we analysed sequence data from all accepted and ratified ICTV taxonomy proposals (TPs) from 2022, and compared results with expert-curated taxonomies. We demonstrate how our software may effectively complement the current gold standard of human curation and allow virologists additional insights into taxonomic classification. We conclude by discussing both the applications and limitations of the software and contrast GRAViTy-V2 with several other widely used and novel tools.

## Materials and methods

### Software

GRAViTy-V2 is written in Python 3.10 and is compatible with bash-like environments. It is distributed as an open-source software package, with installation and operating instructions, via GitHub (https://github.com/Mayne941/gravity2) with a GPL 3.0 license. It may be unpacked and installed, along with all dependencies, on Debian-based operating systems using a packaged bash installer script. A Dockerfile and DockerHub container are provided for users who do not have access to a Linux machine, or administrator credentials on their infrastructure.

The application runs on a local Uvicorn server as a RESTful API, composed in FastAPI (https://fastapi.tiangolo.com/). Users may interact with GRAViTy-V2 either through a browser-based graphical user interface (GUI; SI Document 1, S1.1), or by cURL’ing its endpoints from the command line or appropriate third-party programs. GRAViTy-V2 may also be interacted with via a command line interface (CLI), which may be preferable for expert users who prefer to run software on shared resources. It is designed to be compatible with personal computers but may also be deployed on HPC or cloud infrastructure.

### Algorithm

**Figure 1.**
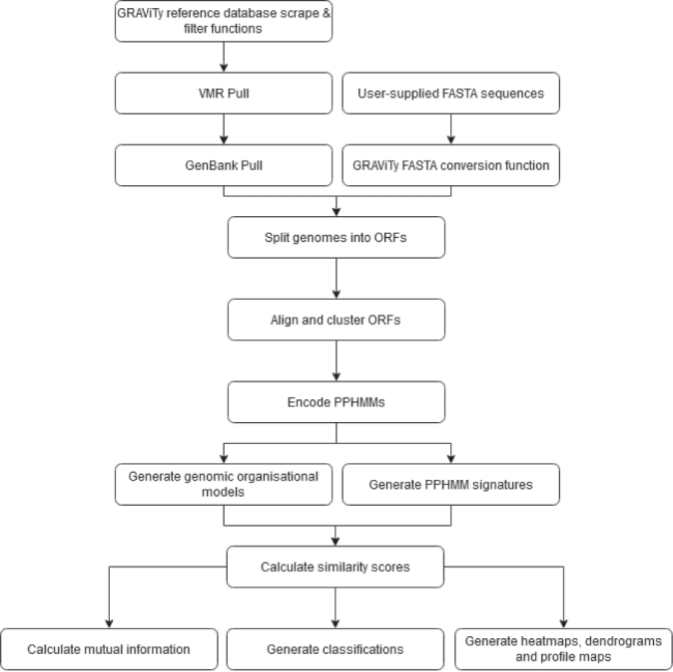
GRAViTy-V2 process flow.

The GRAViTy-V2 framework is a condensed version of the first, 2018 release in which the two pipelines (comparison of reference and unclassified genomes, respectively) have been merged (Figure 1). Input data requirements are a CSV file containing a formatted list of reference sequences and, optionally, additional sequences in FASTA format.

GRAViTy-V2 may be used to evaluate existing classifications, classify new sequences, or a combination of the two. The ICTV-curated Virus Metadata Resource (VMR) lists complete genome sequences of taxonomically classified viruses and provides optimal data for building reference databases. Example workflows for both of these use cases are included in the GRAViTy-V2 Wiki, which is hosted in the software’s GitHub repository. Several new utility functions have been developed to scrape and parse the latest VMR, extract a subset of taxonomically relevant viruses (either user-selected or automatically generated) and generate a database of sequences against which unclassified sequences may be compared.

The GRAViTy-V2 algorithm’s main steps are as follows.

- Read genome description tables.
  - Parse input VMR
  - Build genome description tables containing metadata for each genome
- Generate PPHMMs
  - Where required, pull sequences from GenBank
  - Extract and translate ORFs
  - Compute pairwise protein sequence similarity (users may specify Mash (17) or BLASTp)
  - Cluster protein sequences by pairwise scores (Mcl (18))
  - Create a MSA for each protein sequence cluster (Mafft (19))
  - Compile PPHMM database (HMMER3 (20))
- Annotate protein coding regions
  - Generate PPHMM signature database
  - (Optional) Remove singleton PPHMMs
  - (Optional) Sort PPHMMs
  - Generate GOM database
  - Calculate GOM signatures
  - Compile PPHMM and GOM signature tables
- Make classifications
  - Generate composite Jaccard score similarity matrix from the PPHMM and GOM signature tables.
  - Generate dendrogram, optionally with bootstrap support
  - Generate visualisations
  - (Optional) Calculate virus groupings
  - (Optional) Calculate mutual information

PPHMM generation begins with an algorithm that detects and extracts open reading frames (ORFs) of lengths exceeding a user-set threshold (default 100 amino acids), from coding complete viral genomes (step 2b, above). Where the first version of GRAViTy would use INDSC sequence annotations to identify protein coding regions where available, the current version always opts for *ex novo* ORF detection. This is because it is desirable to normalise PPHMM sizes, and secondly, to avoid spurious results due to sequence annotation errors. ORFs are translated into protein sequences and clustered using Mcl via pairwise similarity scores from either BLASTp (bitscore) or Mash (distance), the latter option being new in version 2 (steps 2c–d). Protein sequences within individual clusters are then aligned to create MSAs with Mafft, before they are encoded as PPHMMs and compiled into a database using HMMER3 (steps 2e–f).

Genome annotation steps generate the two metrics that are used in the final classification system. Firstly, a PPHMM signature table is generated via the HMMER hmmscan utility, which is an array of similarity scores for each virus amino acid sequence against all PPHMMs in the database (step 3a). Users may then opt to remove PPHMM signatures if they are not shared by *n* genomes (via a user-set threshold, step 3b). An additional optional function exists to sort PPHMMs by similarity, which may be used to generate similarity ranks for refining the classification process and may therefore be useful when highly dissimilar viruses are compared.

Secondly, GOMs are generated by encoding of PPHMM genomic locations (absolute position, reading frame and strand orientation) for each virus. GOM signatures may then be computed, which are arrays of absolute coordinate distance correlation scores indicating the overall similarity of PPHMM location profiles against all the GOMs in the GOM database (step 3d–e). Geonomic positions

Classification then begins by computing, for each pair of viruses, two similarity scores. One is the generalised Jaccard similarity index for their PPHMM signatures (*J*_*P*_), and two is the generalised Jaccard similarity index for their GOM signatures (*J*_*G*_), where a generalised Jaccard similarity index is equal to sum(min of every element pair in the signature) ÷ sum(max of every element pair in the signature). The geometric mean of the two scores, the CJS, is then used to represent the overall similarity between the two viruses (Eq. 1) (15).


(1) \begin{eqnarray*} J=\sqrt{J_{P} \times J_{G}} \end{eqnarray*}

Scores of 0 and 1 equate to no detectable similarity and 100% identity, respectively.

Dendrograms are drawn from the resulting similarity matrix using one of six methods (UPGMA default). User options are provided for bootsrapping the generation of both GOM databases and signatures using random subsamples of PPHMMs, with either sumtrees or booster (21) methods (step 4b). All visualisations are then generated (step 4c). Additional visualisations have been developed for version two to enhance model explainability, which include ‘barcode’ protein profile genomic position maps and matrices of normalised ratios of shared PPHMMs between each pair of genomes. These features are all enabled by an algorithm that reconstructs length-normalised representations of genomes as collections of protein profiles, based on information derived from the GOM and PPHMM databases after signature generation.

Final optional steps include making quantitative virus groupings using Thiel’s U statistic, which provides taxonomic group assignments along with a metric of forecast accuracy, and mutual information calculation, which provides a breakdown of each PPHMM’s feature importance in making the final classification (steps 4d-e).

A major algorithm modification in GRAViTy-V2 is the ‘multiple pass system’, whereby searches through very large data spaces are made more efficient by omitting unnecessary information in initial ‘passes’ through a dataset. For example, where a user may have an unclassified viral genome and has no clear idea of its taxonomy, they may run a ‘first pass’, which compares their unknown sequences against representative single genomes (either user-selected or automatically generated) from each family within the realm they are searching, wherein reference sequences may either be selected manually or automatically through a GRAViTy-V2 utility function. This provides an approximate taxonomy that allows the user to conduct a more granular ‘second pass’ run, using a greater quantity of reference genomes from clades most closely related (by similarity score) to the unclassified genome. Selection of second pass reference genomes is currently a manual process as a user’s rules for inclusion will vary, but endpoints are available through both GUI and CLI for filtering input VMRs to user’s selection of taxa. If applied to the realm *Riboviria*, a two pass strategy reduces the search space from approximately 5 × 10^3^ genomes on the first pass to 117 on the second pass and results in an approximately ten-fold reduction in compute time.

### Experimental evaluation

A total of 28 TPs were analysed to evaluate GRAViTy-V2, of which 21 introduced at least one new species, 6 assigned new genera, 4 new families and 2 taxon reorganisations (Table 1). All these proposals had been subject to manual classification by expert members of relevant ICTV study groups. TPs were sourced from ICTV archives (https://ictv.global/files/proposals/approved), all of which contained both tabular and graphical summaries of proposed taxonomies, although there was little consistency in the methods used by the various ICTV study groups to generate their classifications.

**Table 1.** Dataset descriptions and run times^1^

Taxon	ICTV Code	Grouping Level	N	Mean Len (bases)	Time (mins)	Operation
*Jingchuvirales*	2021.015M	*Jingchuvirales*	58	9.75 × 10^3^	1.93	2G, 10S
*Alternaviridae*	2022.001F	*Orthornavirae*	62	3.31 × 10^3^	1.95	1F
*Picornavirales*†	2022.001S	*Orthornavirae*	1004	5.72 × 10^3^	5.61	1F
*Herpesvirales*†	2022.002D	*Herpesvirales*	112	1.69 × 10^5^	506	9S
*Bacilladnaviridae*	2022.002F	*Arfiviricetes*	287	1.93 × 10^3^	3.93	RT
*Alpharhabdovirinae*	2022.002M	*Rhabdoviridae*	273	1.19 × 10^4^	19.9	1G, 14S
*Pestivirus*	2022.002S	*Pestivirus*	21	1.24 × 10^4^	0.72	8S
*Botourmiaviridae*	2022.003F	*Botourmiaviridae*	154	2.73 × 10^3^	1.70	15S
*Arenaviridae*	2022.003M	*Arenaviridae*	63	5.15 × 10^3^	1.83	1G, 1S
*Baculoviridae*	2022.004D	*Baculoviridae*	100	1.34 × 10^5^	110	6S
*Imitervirales*	2022.004F	*Imitervirales*	22	1.51 × 10^6^	150	RT
*Parvoviridae*	2022.005D	*Parvoviridae*	172	5.2 × 10^3^	3.26	2G, 49S
*Mamonoviridae*	2022.005F	*Nucleocytoviricota*	41	2.42 × 10^5^	61.3	1S
*Crispavirus*	2022.006S	*Dicistroviridae*	17	9.4 × 10^3^	0.40	1S
*Cytorhabdovirus*	2022.007M	*Rhabdoviridae*	277	1.19 × 10^4^	20.9	10S
*Ephemerovirus*	2022.008M	*Rhabdoviridae*	269	1.18 × 10^4^	19.3	2S
*Fraservirus*†	2022.010M	*Riboviria*	118	5.94 × 10^3^	5.02	1F
*Hartmanivirus*	2022.001M	*Arenaviridae*	64	5.18 × 10^3^	1.80	2S
*Lispiviridae*	2022.012M	*Lispiviridae*	30	1.20 × 10^4^	1.01	7G, 11S
*Mammarenavirus*	2022.013M	*Arenaviridae*	74	5.67 × 10^3^	2.07	1S
*Mymonaviridae*	2022.014M	*Mymonaviridae*	31	8.94 × 10^3^	0.78	13S
*Nyamiviridae*	2022.016M	*Mononegavirales*	21	1.02 × 10^4^	1.42	2S
*Orthobunyavirus*	2022.017M	*Peribunyaviridae*	143	4.11 × 10^3^	4.65	1S
*Orthobunyavirus*	2022.018M	*Peribunyaviridae*	171	4.64 × 10^3^	5.78	29S, RT
*Phasmaviridae*	2022.019M	*Phasmaviridae*	29	4.18 × 10^3^	0.98	3S
*Phenuviridae*	2022.020M	*Phenuiviridae*	142	4.17 × 10^3^	5.72	2G, 10S
*Varicosavirus*	2022.021M	*Rhabdoviridae*	276	1.16 × 10^4^	2.55	9S
*Vesiculovirus*	2022.022M	*Rhabdoviridae*	269	1.18 × 10^4^	9.13	2S

^1^In cases where multiple passes were used (†), combined times are shown. Grouping level: taxon at which VMR of reference genomes was filtered; N: number of genomes; S: new species operation; G: new genus operation; F: new family operation; RT: reorganise taxon operation.

All sequence data were downloaded automatically from GenBank via GRAViTy-V2 and where multipartite genomes were included, these were assembled in largest-to-smallest order. Experiments were conducted in a single pass except for new family operations, in which cases a two-pass methodology was adopted: representative single genomes from each family within the relevant realm were picked (automatically by the utility function) as reference viruses during the first pass, the purpose of which was to identify the closest three families. For the second pass, sequences from every species from these closest three families were used as reference viruses. Default GRAViTy-V2 parameters were used in the first instance for every dataset, but several were re-run with refined parameters.

Tabular outputs from GRAViTy-V2 were compared with ICTV-generated TP summary spreadsheets using a script which recorded discrepancies in family and genus assignments, in both existing taxa and new or reorganised taxa. All violations were investigated by manually comparing GRAViTy-V2 output with maximum likelihood (ML) trees from TP summaries.

### Hardware

All experiments were conducted on a consumer-grade laptop with an Intel i9-11980HK processor (3.30 GHz, 8 core + 8 threads) and 32 Gb DDR4 RAM (3200 Mhz), via Windows Subsystems Linux 2 running Ubuntu 22.04. Run times were benchmarked in all experiments.

## Results

### Software modifications

Key software modifications were:

- Packaging framework as a multi-operating system application behind an application programming interface (API), with GUI, CLI entrypoints, installer script, extensive error handling and PyTest suite.
- Refactored codebase to run entire workflow from a single pipeline that supports ‘fire and forget’ triggering of single and batch jobs.
- Implementation of a multi-stage workflow, which supports multiple ‘passes’ through reference datasets at different levels of granularity, which reduces run time by removing unnecessary calculations.
- Extensive optimisations, including rewriting the application in from Python 2.7 to 3.10, updating dependencies to latest versions and redesign of compute-intensive functions (PPHMM and GOM signature generation) to memory efficient, parallelised equivalents.
- New algorithm for extracting, assembling and calculating protein profile locations, in a scalable manner that supports accurate comparison of multipartite genomes.
- New output statistics and figures for explaining why the software has made a classification, including maps of protein profile locations within genomes, distance of protein profiles from mean location, and shared profile ratios normalised to genome length.
- Development of an optional, alternative similarity matrix scoring scheme that weights PPHMM signatures by ratio of shared PPHMMs for use with very large genomes.
- Support for use of Mash scores rather than BLASTp for initial distance estimation, which can reduce run times and enhance sensitivity in certain conditions.
- New utility functions to support taxonomic investigations, including FASTA to GRAViTy-V2-compatible input converter, utility for consistently concatenating multipartite genomes into ordered, linear sequences and web scraper function to gather the latest VMR version from the ICTV website.
- Creation of several ‘premade’ pipelines with parameter configurations optimised for specific scenarios (e.g. comparing highly divergent viruses), for which users only need specify three parameters.
- Option to export contiguous blocks of sequence in regions where PPHMM matches can be detected. The identification and extraction of sequences (e.g. RdRP and helicase regions of RNA viruses) greatly assists parallel analyses of virus relationships though conventional alignment and phylogeny methods.

**Figure 2.**
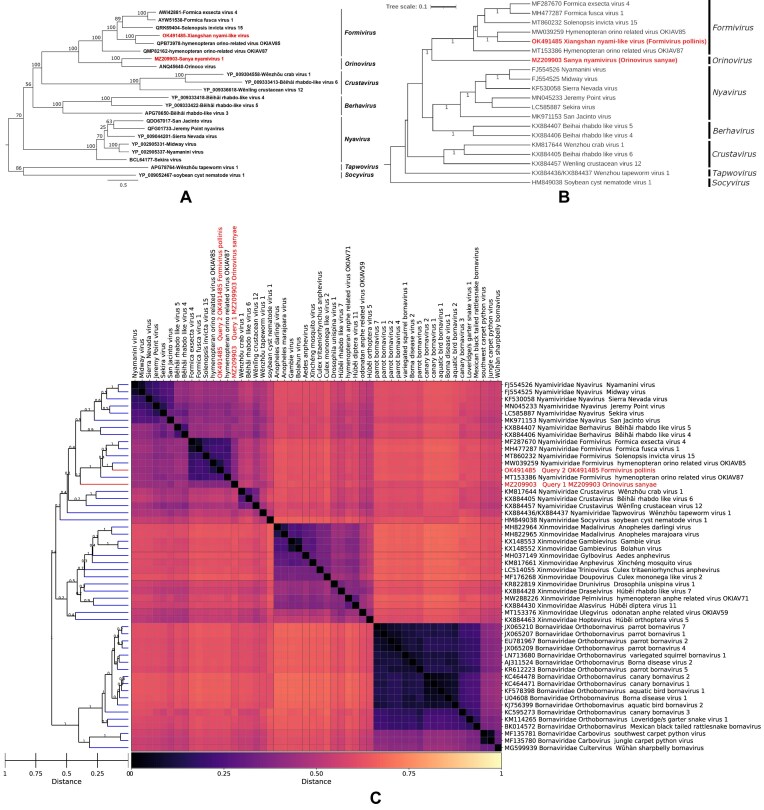
Comparison of ICTV and GRAViTy-V2 analyses for *Nyamiviridae* TP. (**A**) TP ML tree, from L protein AA alignment (22). (**B**) GRAViTy-V2 tree (Orinocovirus and Beihai rhabdo-like virus 3 omitted as genomes non-coding complete; bootstrap values <0.7 hidden). (**C**) GRAViTy-V2 heatmap, including neighbouring families *Xinmoviridae* and *Bornaviridae*. (Red text: sequences proposed as new taxa in TP).

**Figure 3.**
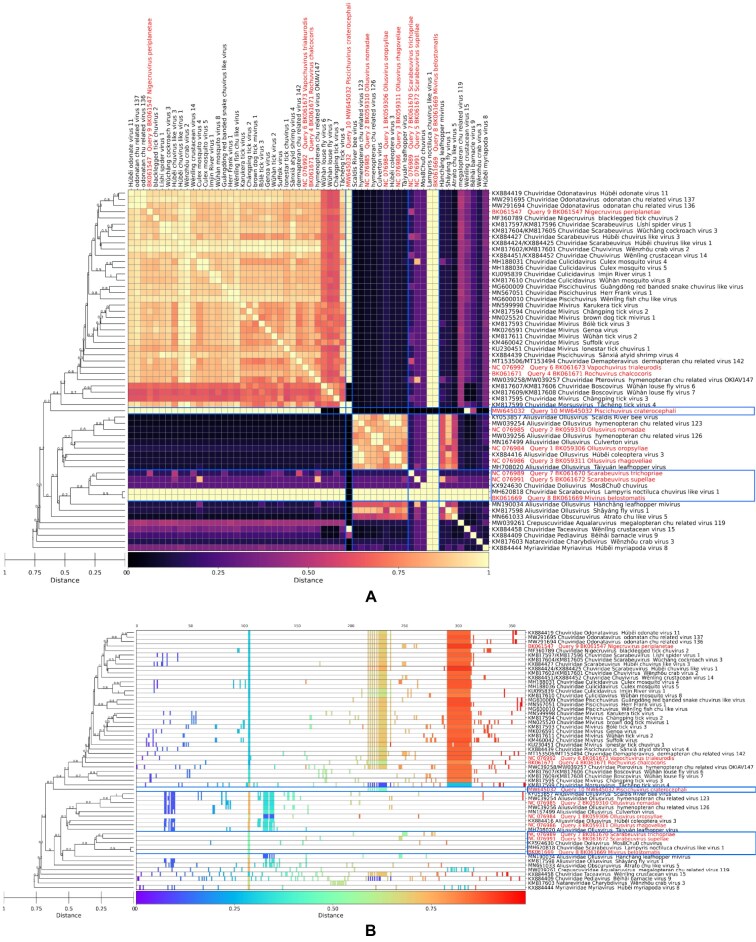
GRAViTy-V2 analysis of *Jingchuvirales* TP. (**A**) Shared normalised PPHMM ratio matrix. (**B**) Barcode heatmap. (Blue lines: incomplete sequences; red text: sequences proposed as new taxa in TP).

GRAViTy-V2 graphical outputs include:

- ‘GRAViTy-V2 heatmap’, where cell colour intensity corresponds to pairwise similarity score (as per the user-set similarity scheme used) between all genomes, accompanied by a bootstrapped dendrogram (Figure 2C). Darker cells correspond to higher degrees of similarity. These are similar to the original GRAViTy heatmaps but now incorporate more complete labelling, auto-scaling and a range of user-set customisations for producing publication-quality images.
- ‘Shared normalised PPHMM ratio matrix’, where cell colour intensity shows pairwise comparison of quantity of shared PPHMMs, normalised to the number of profiles assigned to each genome (Figure 3A). Darker cells correspond to higher ratios of shared PPHMM signatures and indicate greater quantities of shared information between genomes.
- ‘Barcode heatmap’, showing genomic position of each PPHMM midpoint, normalised against genome length (heatmap colour) verses median position among all genomes (X-axis) (Figure 3B). A vertical block of coloured cells at a position approximately half way along the X axis would indicate a non-zero PPHMM signature shared by all genomes whose midpoint sits, on average, half-way along the genome. Differing colour intensity in this vertical row would indicate variation in the relative position the PPHMM compared to the median.

Full graphical output from all experiments are included (SI Document 2).

### Run time benchmarking

**Figure 4.**
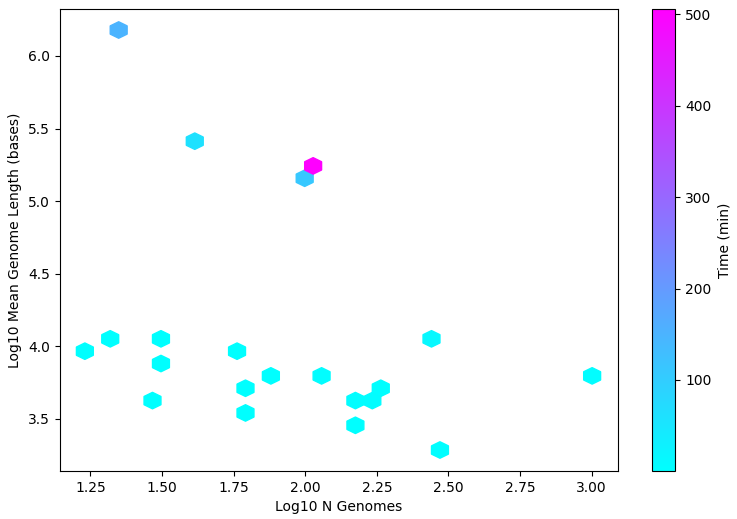
Histogram of GRAViTy-V2 run time benchmarks showing positive correlation with both mean genome length and number of genomes analysed.

Run times for each TP were benchmarked against mean genome size and the number of genomes compared (Table 1), and varied from 1 to 506 min (median 3.59) across datasets of size 21–1004 genomes. Time scaling increased with both mean genome size and number of pairwise comparisons (Figure 4). Total compute time across all experiments was approximately 16 hours and the batch was run in a single session. The longest run time outlier was for the *Herpesvirales* dataset, which was notable for it containing a large number of comparatively long genomes, for which two passes were run. HPC infrastructure was not required for our use case.

A subset of TPs (10) was run using the original GRAViTy version. Equivalent runs on GRAViTy-V2 completed 40–66% more quickly (median 52%). Experiments with original GRAViTy completed with more violations in 5/10 TPs and failed in 2/10, due to memory overflow in both cases (SI Document 3).

### Comparison between GRAViTy-V2 results and expert-curated taxonomies

**Figure 5.**
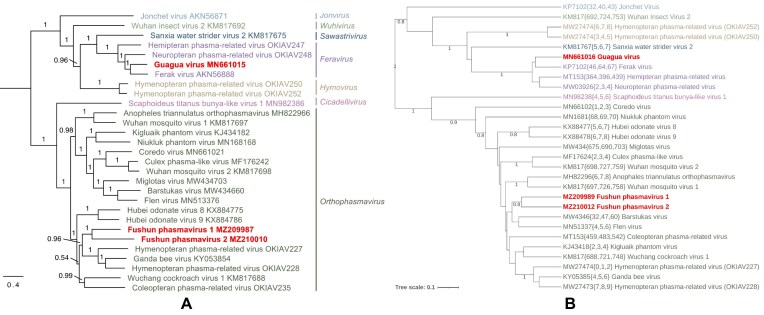
Comparison of ICTV and GRAViTy-V2 analyses for *Phasmaviridae* trees. (**A**) TP ML tree produced by alignment of L segment AA sequences (23). (**B**) GRAViTy-V2 tree, with colours adjusted to match TP. (Red text: sequences proposed as new taxa in TP; bootstrap values <0.7 hidden).

Overall, GRAViTy-V2 output reproduced patterns of phylogenetic clustering consistent with expert-curated taxonomies. Remarkably, this concordance was consistent across the diverse phylogenetic methods used by the study groups, which ranged from simple alignments of single RdRp genes (e.g. *Phasmaviridae*, Figure 5) to complex, curated alignments of multiple marker genes (e.g. *Mamonoviridae*). Overall, 22/28 classifications exhibited no family or genus-level violations between ICTV ratified taxonomic classifications and relationships determined by GRAViTy-V2 (Table 2).

**Table 2.** Results of GRAViTy-V2 TP analysis compared to curated classifications, with original TP taxonomic methods (tools listed where information provided in TP)^1^

Taxon	N	NF	NFV	NG	NGV	TP Method	Violations
*Jingchuvirales*	58	5	5	21	2	ML tree (PhyML) from RdRp alignment (ClustalW/Muscle, with manual curation).	6 incomplete genomes (BK061669, BK061670, BK061672, MW645032, MH620818, KX924630) causing FV/GVs.
*Alternaviridae*	62	6	0	11	0	ML trees from RdRp alignments; positions with low coverage eliminated.	
*Picornavirales*	1004	118	0	1004	0	Maximum-parsimony trees from predicted RdRp and helicase domain alignments.	
*Herpesvirales*	112	3	0	22	8	Neighbour-joining trees from alignments of concatenated predicted AA sequences of six conserved genes.	GVs 3 × *Rhadinovirus*, 2 × *Percavirus* genomes rearranged within *Gammaherpesvirinae*. GVs 3 × *Mardivirus* split main cluster, rearranged within *Alphaherpesvirinae*.
*Bacilladnaviridae*	287	8	0	41	3	ML trees (PhyML) constructed from alignments (Mafft) of representative AA sequences; unsupported branches collapsed (TreeGraph2).	GVs *Circoviridae* MH545516, KT73278{5–6} cluster with *Nenyaviridae*.
*Alpharhabdovirinae*	273	1	0	45	0	ML trees from complete L protein sequence alignments.	
*Pestivirus*	21	1	0	2	0	ML tree from alignment of RdRp sub-domain (conserved NSB5 peptide).	
*Botourmiaviridae*	154	1	0	12	2	ML trees (IQ-TREE) from multiple RdRp AA alignments (Mafft); low quality regions trimmed (trimAI).	GV *Magoulivirus* MK189195 clusters with *Betabotoulivirus*. *Rhizoulivirus* outgroups from *Betarhizoulivirus*.
*Arenaviridae*	63	1	0	5	0	ML trees from complete L and NP protein AA alignments.	
*Baculoviridae*	100	1	0	4	0	ML tree (RAxML) from concatenated alignments of 38 core gene AA sequences.	
*Imitervirales*	22	1	0	15	0	ML tree (IQ-TREE) from concatenated alignment of seven marker genes.	Sections of MT663534, KU877344 non-coding, but no violations.
*Parvoviridae*	172	1	0	29	4	Bayesian inference on three domains of the NS1 protein (BEAST, PhyML).	GVs *Sandeparvovirus* OK236393 and *Muscodensovirus* MT498824 outgroup. GVs *Dendroparvovirus* MG74567{0,7} cluster with *Erythroparvivirus*.
*Mamonoviridae*	41	7	0	39	0	ML tree (IQ-TREE) from concatenated AA alignment of seven marker genes (Mafft).	
*Crispavirus*	17	1	0	3	0	ML trees from nonstructural ORF alignments.	
*Cytorhabdovirus*	277	1	0	45	0	ML trees from L protein sequence alginments (Muscle)	
*Ephemerovirus*	269	1	0	45	0	ML tree from alignment of complete L protein sequence alignments.	
*Fraservirus*	118	3	0	20	0	ML trees (IQ-TREE) from RdRp and bunyaviral-like glycoprotein alignments.	
*Hartmanivirus*	64	1	0	5	0	Distance matrices for L and S segments.	
*Lispiviridae*	30	1	0	24	0	ML trees (ModelTest-NG) from RdRp AA alignments (Mafft).	
*Mammarenavirus*	74	1	0	10	0	Bayesian trees from nucleotide GPC, NP and L gene alignments.	
*Mymonaviridae*	31	1	0	8	0	ML tree (IQ-TREE) from RdRp AA alignments.	
*Nyamiviridae*	21	3	0	22	0	ML tree (IQ-TREE) from L protein AA alignments (Mafft).	
*Orthobunyavirus* 017M	143	1	0	7	0	ML trees from alignments of S, M and L segments.	
*Orthobunyavirus* 018M	171	1	0	7	1	ML tree from alignments of L segment (ClustalW).	GV *Orthobunyavirus* MK89661{5–7} outgroups, M segment (MK899616) doesn’t code for any ORFs.
*Phasmaviridae*	29	1	0	8	0	ML tree from AA alignments of L segment (Mafft).	
*Phenuviridae*	142	1	1	21	6	ML tree (IQ-TREE) from RdRp AA alignments (Mafft).	FV MW74189{4–6} all segments partial CDS with $\sim 30\%$ gaps. GVs 6 × double-segmented *Uukuvirus* cluster away from triple-segmented *Uukuvirus*.
*Varicosavirus*	276	1	0	6	0	ML tree from L protein AA sequences (Mafft).	
*Vesiculovirus*	269	1	0	31	0	ML tree from L protein AA sequences (Muscle).	

^1^N: Number of species; NF{/V}: Number of families / family-level violations compared to corresponding TP; NG{V}: Number of genera/ genus-level violations; CDS: (protein) coding sequence; ML: maximum likelihood.

**Figure 6.**
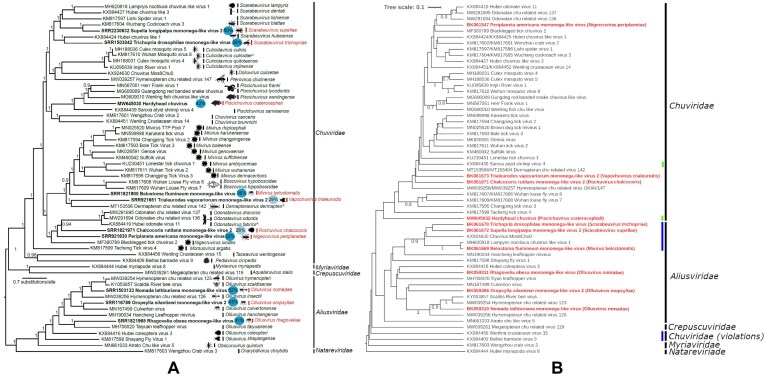
Comparison of ICTV and GRAViTy-V2 analyses for *Jingchuvirales* TP. (**A**) TP ML tree, from RdRp alignment (24). (**B**) GRAViTy-V2 tree (red: sequences proposed as new taxa in T; blue: family violations; green: genus violations; bootstrap values <0.7 hidden).

Of the comparisons yielding different virus relationships between methods, two of these violations were at family-level, in the *Phenuiviridae* and *Jingchuvirales* datasets (Table 2). In the former, the outlier was a tripartite genome with three coding incomplete GenBank sequences that precluded a valid comparison with their current taxonomy, as GRAViTy-V2 requires coding complete genomes for estimation of sequence relationships. In the *Jingchuvirales* set, five of seven family-level (and one genus) violations were caused by the original inadvertent inclusion of a large number of incomplete genomes by the Study Group responsible for the original classification, which was based on an RdRp alignment (Figure 6).

Incomplete sequences were identifiable in shared normalised PPHMM ratio and barcode heatmaps, as long bands of continuous colour and absent profiles, respectively (Figure 3). This demonstrates how manual assessment of GRAViTy-V2 output led to identification of violations resulting from either directly involved the incomplete genome sequences themselves (five), or indirectly where complete genomes were pulled out of their correct family assignment through their similarity to the incomplete sequences. In consequent experiments, the *Jingchuvirales* dataset was improved by locating missing components of four incomplete sequences (BK061669, KX924630, MW645032, MH620818) and removing those for which no replacements could be found (BK0616{70,72}), which reduced the number of family violations to zero and the number of genus violations to one (SI Document 1, S1.2).

Analyses of datasets from seven TPs resulted in genus-level violations (Table 2) and the majority of these exhibited sub-genus variation in TP-derived trees based on phylogenetic analysis of aligned conserved genome regions. The rearrangements observed in the GRAViTy-V2 output did not however violate their original taxonomic classifications. In investigating the causes of these discrepancies, two genus violations were the result of the original inclusion of non-coding complete sequences for which replacements could not be found, leaving five datasets where there were genuine discrepancies. These tended to occur in instances where clades contained genomes with either a variable quantity of segments (e.g. *Bacilladnaviridae*, SI Document 2, Supplementary Figures S9–S10); a single, very large ORF (e.g. *Mymonaviridae*, SI Document 2, Supplementary Figures S41–S42); or very remote homologies at the limit of what PPHMM profile comparisons can detect, as indicated by barcode graphs (*Herpesvirales*, SI Document 2, Supplementary Figures S7– S8).

Several TPs proposed the creation of new taxa and GRAViTy-V2 results were concordant with these, notwithstanding the violations listed (e.g. *Alternaviridae*, SI Document 2, Supplementary Figure S3). Where this is not always obvious in visual inspection of GRAViTy-V2 heatmaps, especially at lower ranks, the software additionally estimates virus groupings including new taxon assignments, results for which are automatically reported in tabular output. Assignment of taxonomic rank in GRAViTy-V2 is achieved using a bespoke similarity scoring system, which exemplifies how methods that do not require MSAs as input may be used to assign new taxa in the absence of reference sequences with definite homologies.

## Discussion

### GRAViTy-V2 performance and best practice

GRAViTy-V2 has more than thirty optional parameters, the majority of which control the command line tools the framework uses (Mash, BLASTp, Mafft, HMMER, HHsuite). While every attempt has been made to simplify the user experience, there is no single set of default parameters that will suit the requirement of every user. However, only a few parameters significantly influence classifications, specifically the Mash similarity threshold, minimum protein length, use of BLASTp or Mash and HMMER bitscore threshold. These parameters should be selected carefully and with reference to knowledge of the genomes being analysed. We have created several easy-to-use premade pipelines with parameter sets optimised for specific scenarios (comparison of similar, divergent or extremely long viral genomes) for which users only need specify their input VMR, folder for storing output and, optionally, input sequences if they are not available on GenBank.

Experiment duration was proportional to the length and number of genomes analysed, and the optional user-selected parameters. Some parameters may significantly increase run time and are not likely to improve classifications in the majority of use cases (e.g. alignment merging, PPHMM sorting). Conversely, two mandatory parameters that all users should optimise are ‘NThreads’ and ‘N_bootstrap’, which dictate the level or parallelism based on the number of CPU cores available, and the number of bootstrap iterations for tree building, respectively. Users should use default parameters in the first instance and refine them iteratively as required. A user guide for optimising GRAViTy-V2 parameters is included in the GitHub repository Wiki. It is advised to not classify datasets of >1000 genomes with GRAViTy-V2 in a single pass and instead use a multiple pass methodology should users wish to keep run times within the range of minutes, rather than hours.

### Mismatches between GRAViTy-V2 results and ICTV ratified TPs

Input sequence quality was found to be the cause of all cases of family violations and approximately one fifth of genus violations. Inclusion of non-coding complete sequences, such as in the *Jingchuvirales* dataset (Figure 6), or segmented genomes assembled inconsistently (SI Document 1, S1.3), were found to dramatically impact classifications. In initial experiments, four datasets exhibited genus-level violations as result of including incorrect (mislabelled accession IDs or incomplete genome) sequences, all of which were corrected manually and re-run.

It was simple to identify problematic sequences in GRAViTy-V2 graphs (Figure 6; SI Document 1, S1.4), whereas this is traditionally a challenging manual task when working with large quantities of novel genomes, especially when they are metagenomically derived. These observations highlight how taxonomists should be mindful of sequence quality in public databases.

There were several scenarios in which GRAViTy-V2 did not perform optimally, as measured by genus-level violations. Foremost were instances where viruses had single, extremely long ORFs, such as in the *Mymonaviridae*. The minimal genomic unit by which features are compared in GRAViTy-V2 is the ORF and hence, comparing the protein sequence similarity and genomic locus of single polyprotein genes between multiple viruses yields comparatively little information. Our solution here was to use a different similarity scoring scheme that omitted the contributions of the GOM and focused only on PPHMM signature similarity scores. For the same reason, GRAViTy-V2 is unlikely to classify two genomes within the same taxon if they contain a different number of segments, which was the cause of violations in the *Phenuiviridae* dataset.

### Use cases for GRAViTy-V2 in comparison with other tools

When choosing viral taxonomy software, there is currently no package that will suit the needs of every user. Certain tools rely on building pairwise distances between genomic components, including PASC (11) and newer derivatives such as DEmARC (25). These algorithms are effective for differentiating between similar genomes at species and often genus level, but not in defining similarity thresholds for family or higher rank assignments. VISTA (26) is an increasingly popular tool for lower rank assignments that greatly builds on the pairwise similarity paradigm and is highly scalable, but is, however, reliant on a high reference data input requirement and prone to bias should any taxa be over-represented.

Several alignment-free tools have emerged recently for classifying viruses infecting bacteria and archaea (e.g. vConTACT (27), VIRIDIC (28) and ViPTree (29)). These use sequence identity as base metrics in combination with network and clustering algorithms. While these approaches are comparatively rapid and effective for screening larger viruses, they are not complete taxonomic tools and are usually not phylogeny-aware.

Virus relationships as determined by GRAViTy-V2, conversely, are based on holistic comparisons of all coding regions within virus genomes. GRAViTy-V2 classifications are therefore not based on rules for taxonomic assignments of specific virus groups, such as the reliance on specific marker or hallmark genes, nor on assignments based on the adoption of a variety of threshold similarity values to define genera and species. Methodologies based on the properties of individual virus families, genera or species definitions, remain in the province of expert curation and cannot be reproduced by a general metric, including GRAViTy-V2’s GCJ. Our software can however provide an often highly informative measure of genetic relationships through analysis of multiple extracted genomic features. We therefore envisage the following uses for our software in the analysis of virus metagenomic sequence data:

- Running GRAViTy-V2 requires only the test sequence(s) or its accession number. Comparisons are made with the dataset of classified viruses accessed from ICTV databases (i.e. VMR) by the program itself. It therefore provides a useful screening tool for initial evaluation of virus sequence data and provisional assignments to orders, families or lower taxonomic ranks. Its output is far more informative than methods such as Kraken for the analysis of metagenomic sequence data that simply report profile or *k*-mer matches.
- Unlike MSA-based methods, GRAViTy-V2 can also identify and incorporate sequences into the analysis that possess no identifiable homology to existing classified viruses, corresponding to entirely new virus groups and component taxa. Conversely, methods based purely on MSAs cannot depict evolutionary relatedness between non-homologous sequences in its output.
- GRAViTy-V2 provides a general tool for re-examining existing classifications, and identification of incomplete or incorrectly assembled sequences, as exemplified in many of the reported analyses in the study.
- The reporting of positions of homology through PPHMM matching (Figure 3b) allows the identification of conserved motifs in stretches of contiguous sequence. These can be selected, exported and used for further analyses. Particularly for very distantly related viruses, the cumbersome and often manual identification, extraction and alignment of conserved genome regions can be avoided through using GRAViTy-V2 output.

## Conclusion

Evaluations of official TPs were completed within the scale of minutes to hours and were found to generate classifications similar to those created by expert groups. GRAViTy-V2 aided identification of several human errors, accidental inclusion of non-coding complete sequences and remote homologies (or lack thereof) between genomic components not identified in conventional, conserved region alignments. We propose that GRAViTy-V2’s approximate classifications are suitable for use in support of viral taxonomy workflows including, but not limited to, classification of newly-described viruses.

## Supplementary Material

lqae183_Supplemental_Files

## Data Availability

Data supporting this article (TPs and VMR) were accessed from the ICTV website https://ictv.global/. GRAViTy-V2 software is available via GitHub (https://github.com/Mayne941/gravity2) with a GPL 3.0 license and an imprint of the software version current with this manuscript is hosted at Zenodo (https://doi.org/10.5281/zenodo.13911725).
