# Supplementary material for: GRAViTy-V2: a grounded viral taxonomy application
Source: NAR Genom Bioinform. 2024 Dec 18;6(4):lqae183. doi: 10.1093/nargab/lqae183 (PMC11655284; doi:10.1093/nargab/lqae183)
Supplement: lqae183_Supplemental_Files [file lqae183_supplemental_files.zip › GRAViTy_manuscript_SI_Document_1.docx]

**GRAViTy-V2: a grounded viral taxonomy application**

**Mayne, R., Aiewsakun, P., Turner, D., *et al.* (2024)**

**Supplementary information, Document 1**

**S1.1 GRAViTy-V2 graphical user interface**


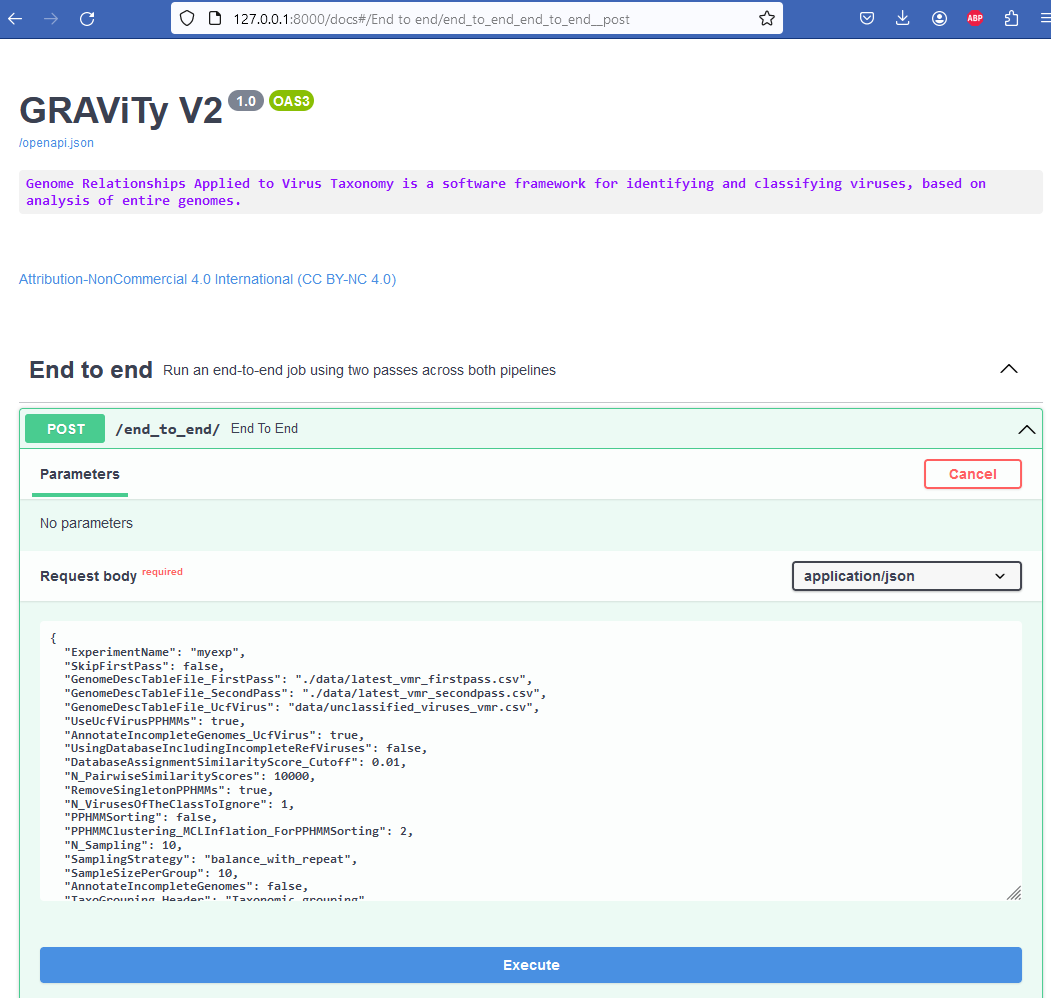


Fig. S1.1.1. GRAViTy-V2’s graphical user interface, as accessed via a browser. View shows the main end-to-end endpoint (function) window expanded, revealing the dialogue box and “execute” button that may be used to refine and trigger experiments, respectively.

**S1.2 Jingchuvirales dataset comparison, TP-derived sequences verses fixed**


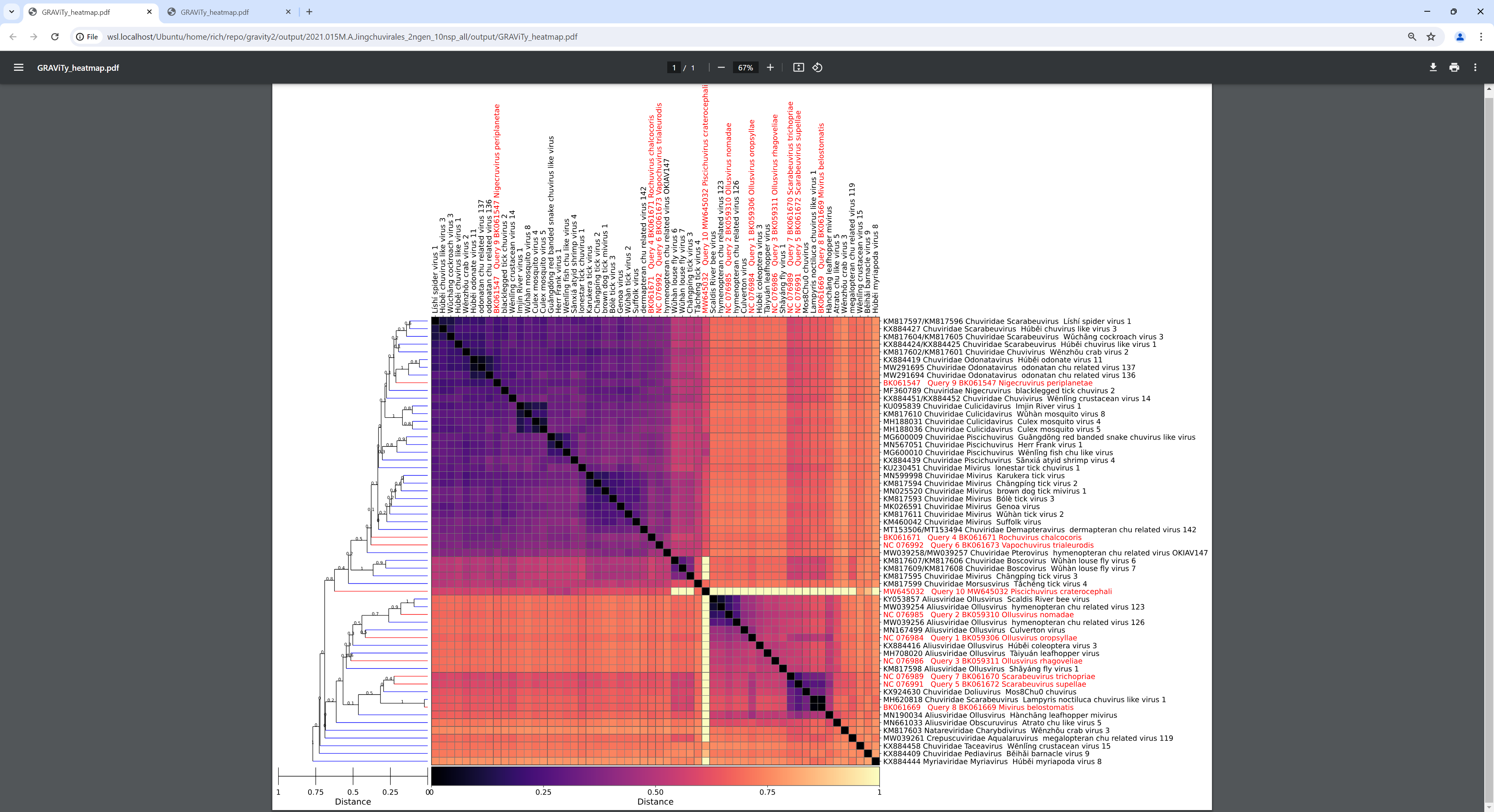


(a)


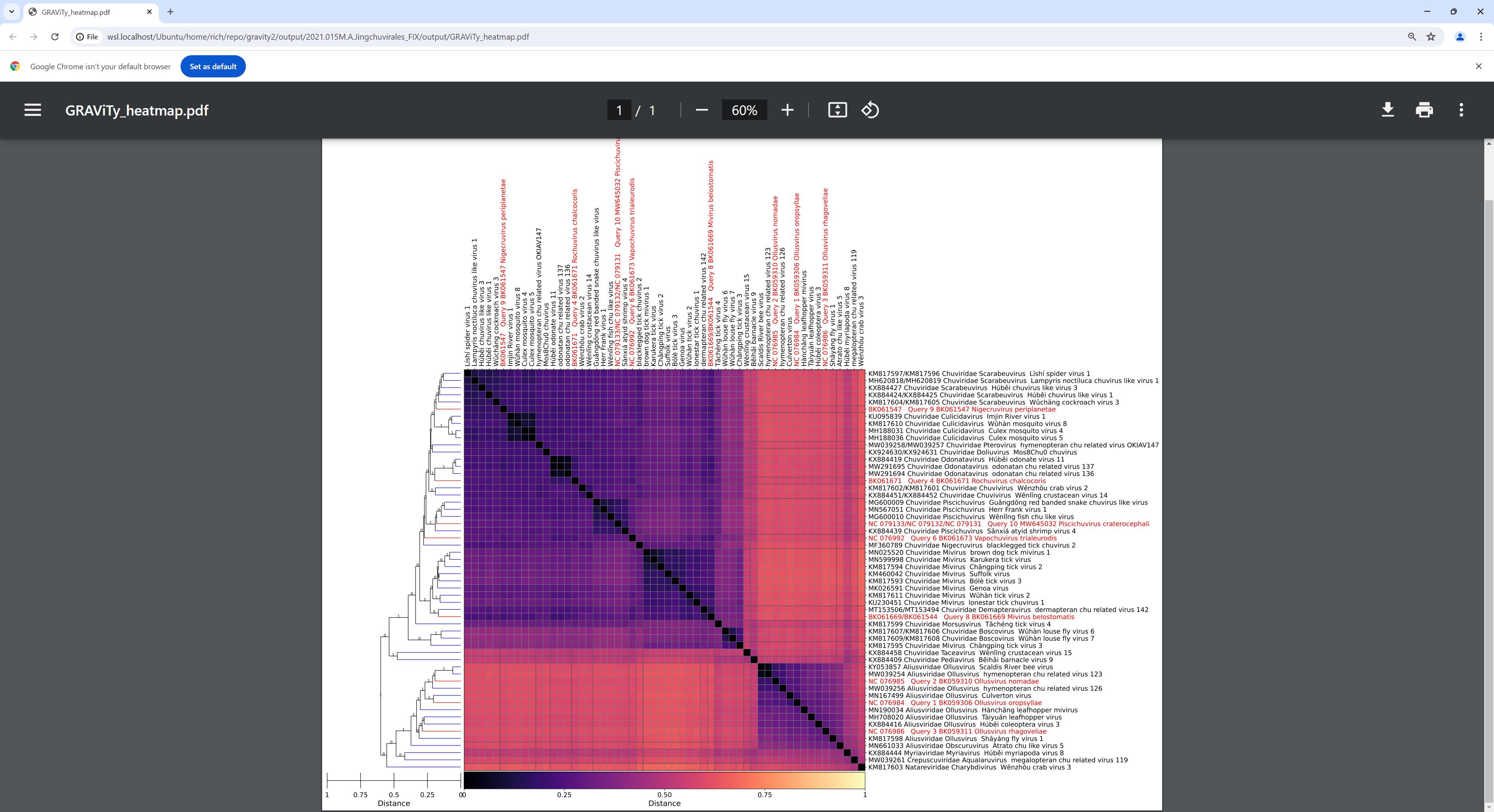


(b)

Fig. S1.2.1. GRAViTy-V2 heatmaps, Jingchuvirales. (a) Using sequences derived from TP, with 8 family violations and 2 genus violations due to including 6 incomplete sequences (BK061669, BK061670, BK061672, MW645032, MH620818 KX924630). (b) Corrected 4 sequences (BK061669, KX924630, MW645032, MH620818) and removed remaining 2 for which no replacements could be found (BK061670, BK061672), leaving a single genus violation (KM817595).

**S1.3 Effect of input sequence quality**


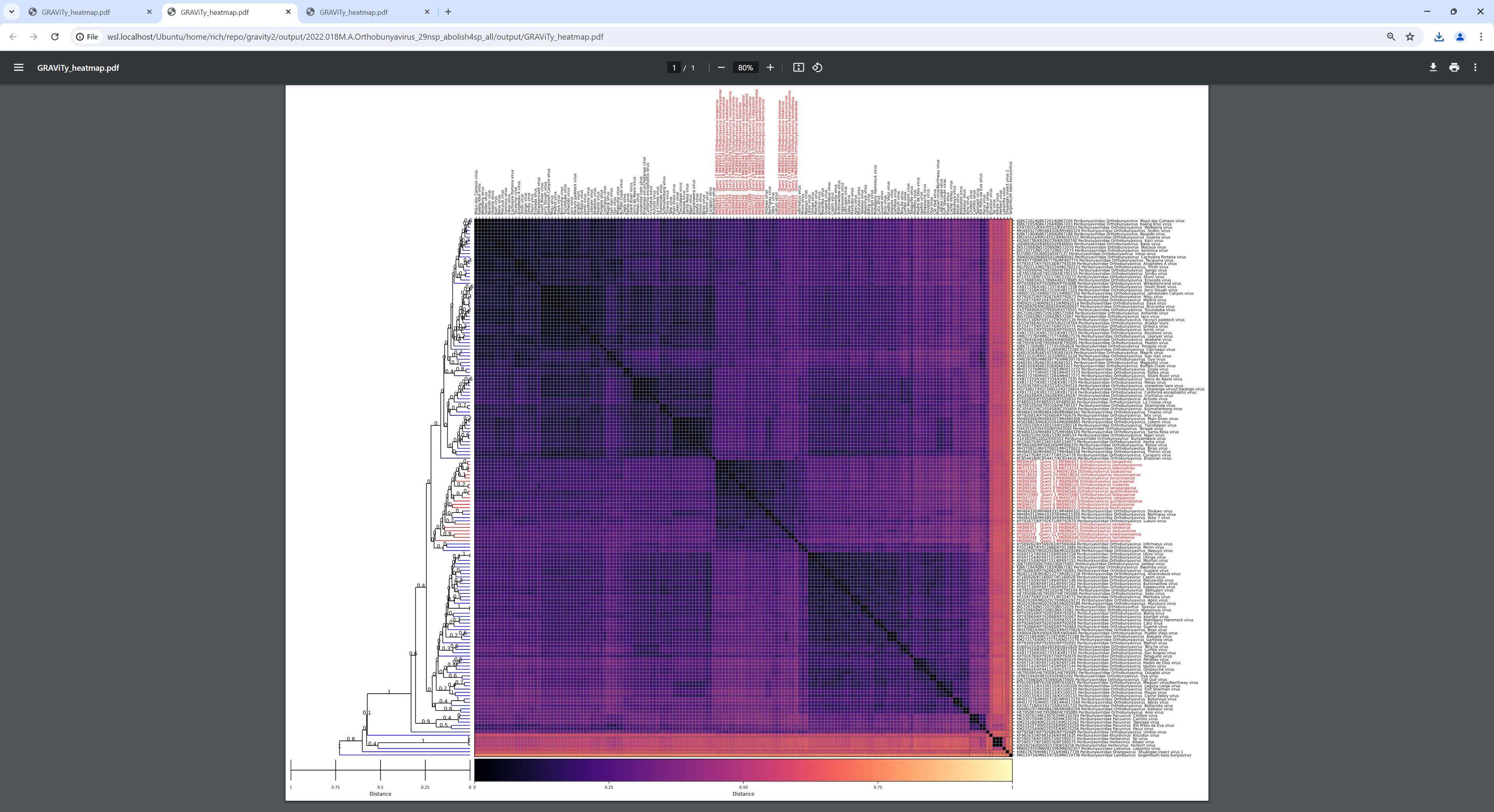


(a)


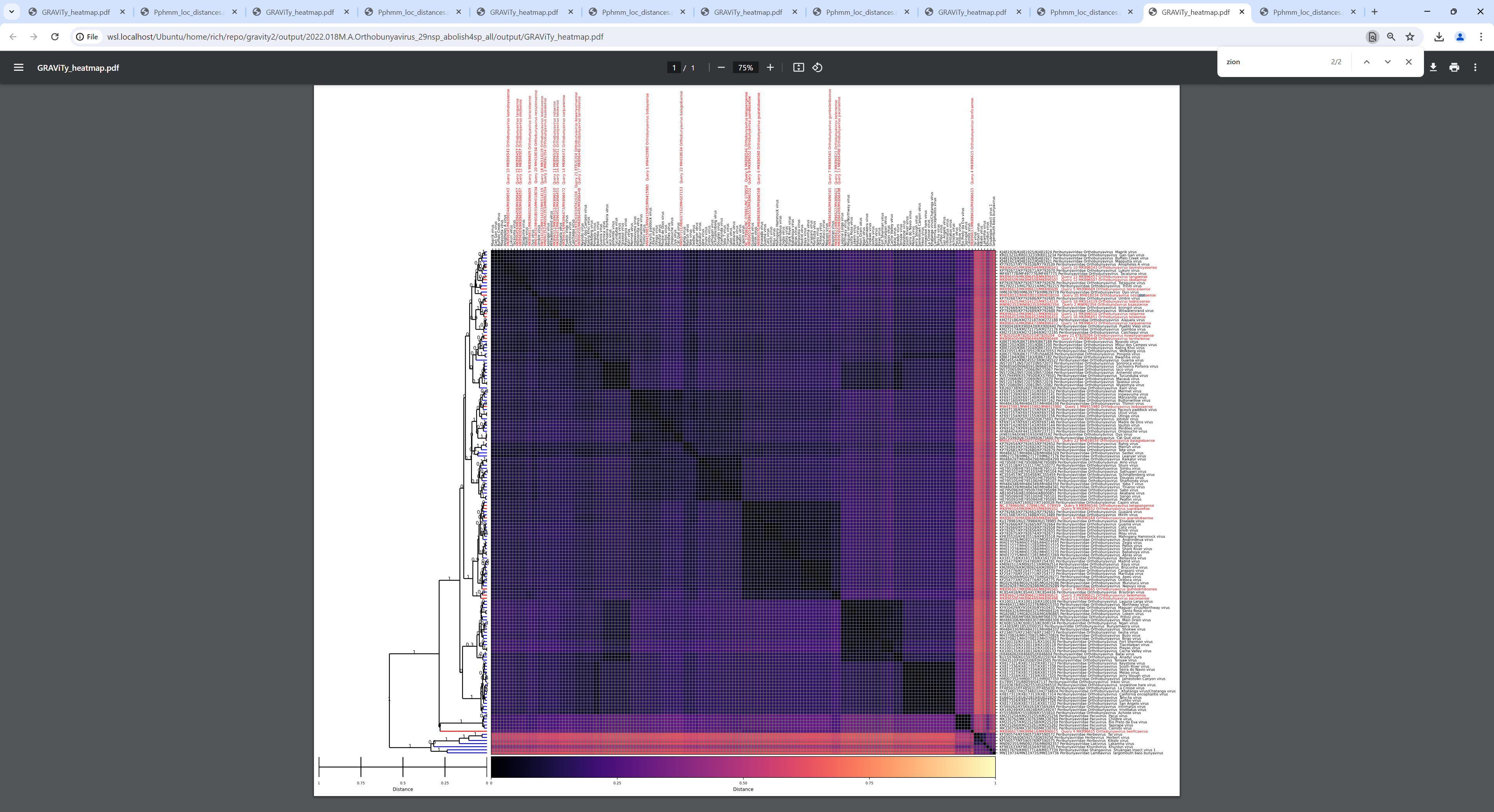


(b)

Fig. S1.3.1. GRAViTy-V2 heatmaps, Orthobunyavirus (018M). (a) Input unclassified sequences (red) were provided pre-assembled rather than being assembled by GRAViTy-V2 from GenBank sequences. Assembly was inverse order to classified sequences, causing unclassified sequences to cluster together incorrectly. (b) As in <a>, with correct assembly of unclassified sequences.

**S1.4 Identification of low-quality input sequences with GRAViTy-V2 output**


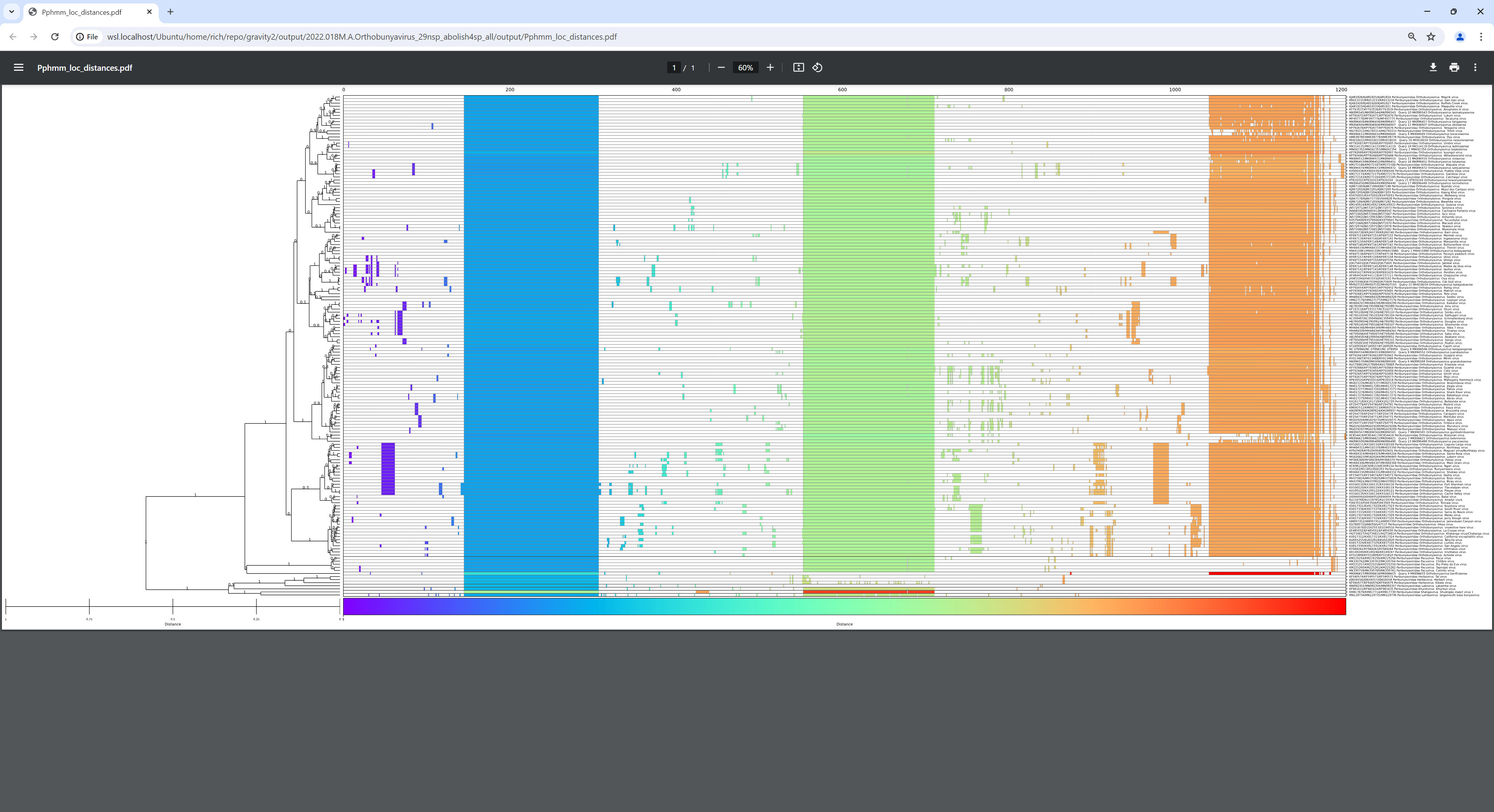


Fig. S1.4.1. Barcode, Orthobunyavirus (018M). Genus violation MK896615-7 (*Orthobunyavirus benficaense)* shows missing profile section in central (green) block, corresponding to M segment which is non-coding. Corresponds to heatmap above (Fig. S.4.1).
