# Supplementary material for: GRAViTy-V2: a grounded viral taxonomy application
Source: NAR Genom Bioinform. 2024 Dec 18;6(4):lqae183. doi: 10.1093/nargab/lqae183 (PMC11655284; doi:10.1093/nargab/lqae183)
Supplement: lqae183_Supplemental_Files [file lqae183_supplemental_files.zip › GRAViTy_manuscript_SI_Document_2.pdf]

GRAViTy-V2: a grounded viral taxonomy application

Mayne, R., Aiewsakun, P., Turner, D., Adriaenssens, E. and Simmonds, P. (2024)

Supplementary information, Document 2

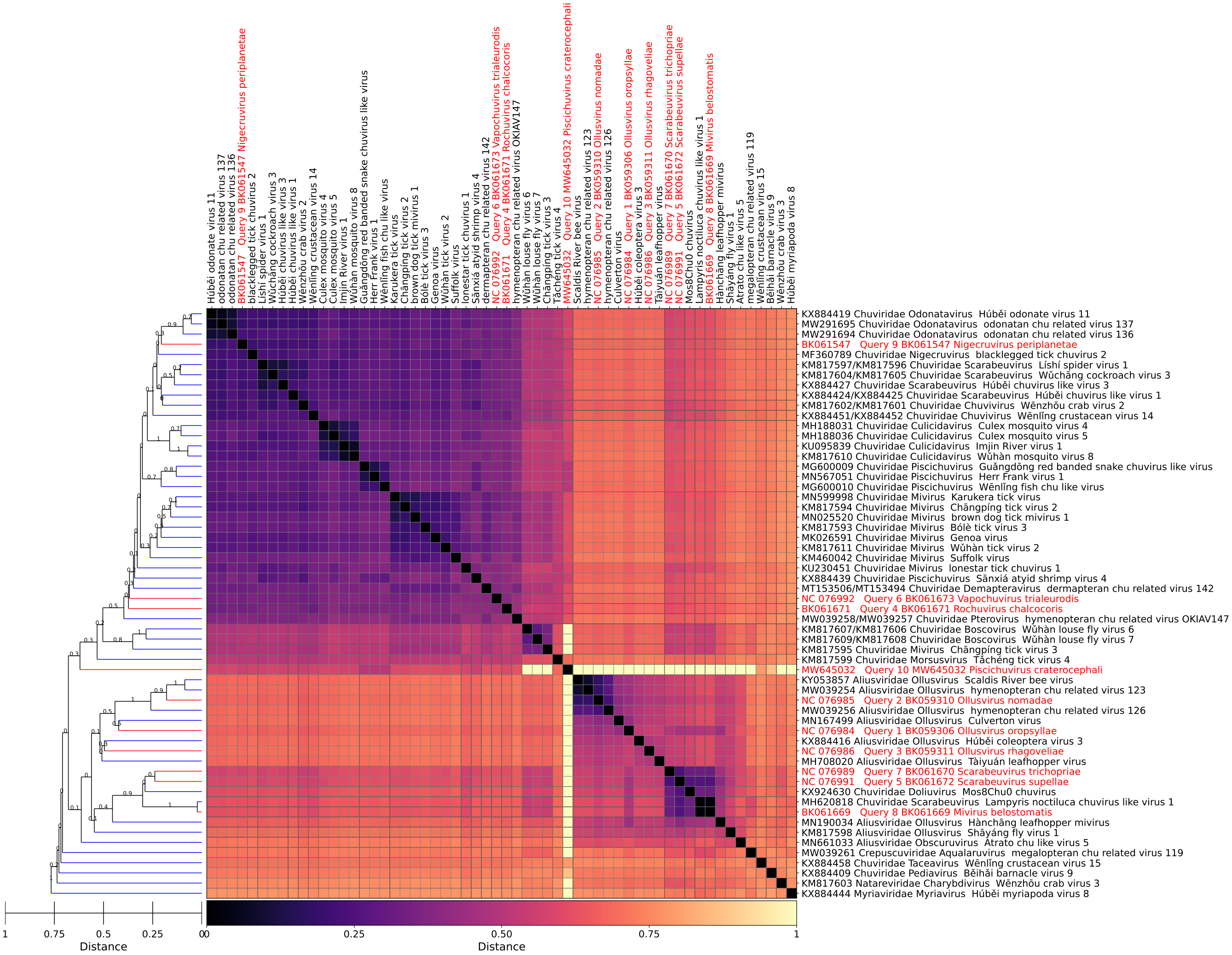

Figure 1: *Jingchuvirales*, GRAViTy-V2 heatmap

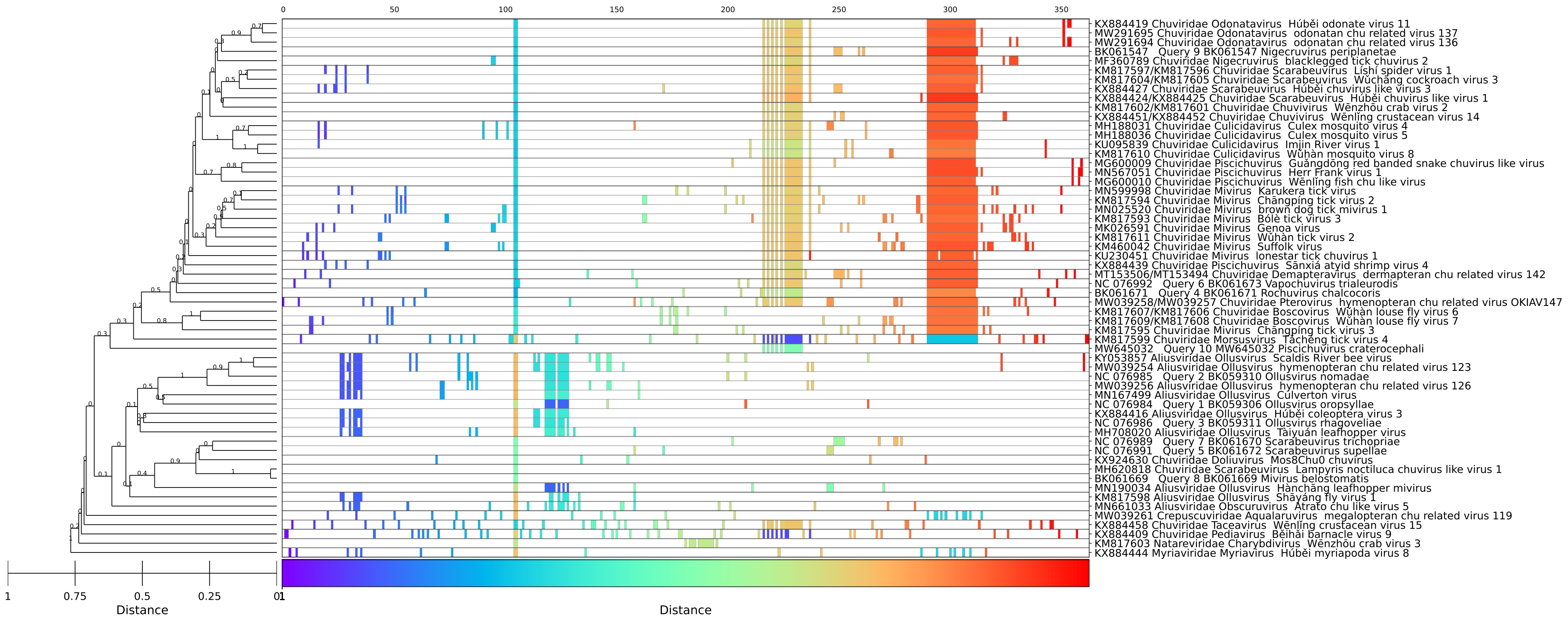

Figure 2: *Jingchuvirales*, GRAViTy-V2 barcode

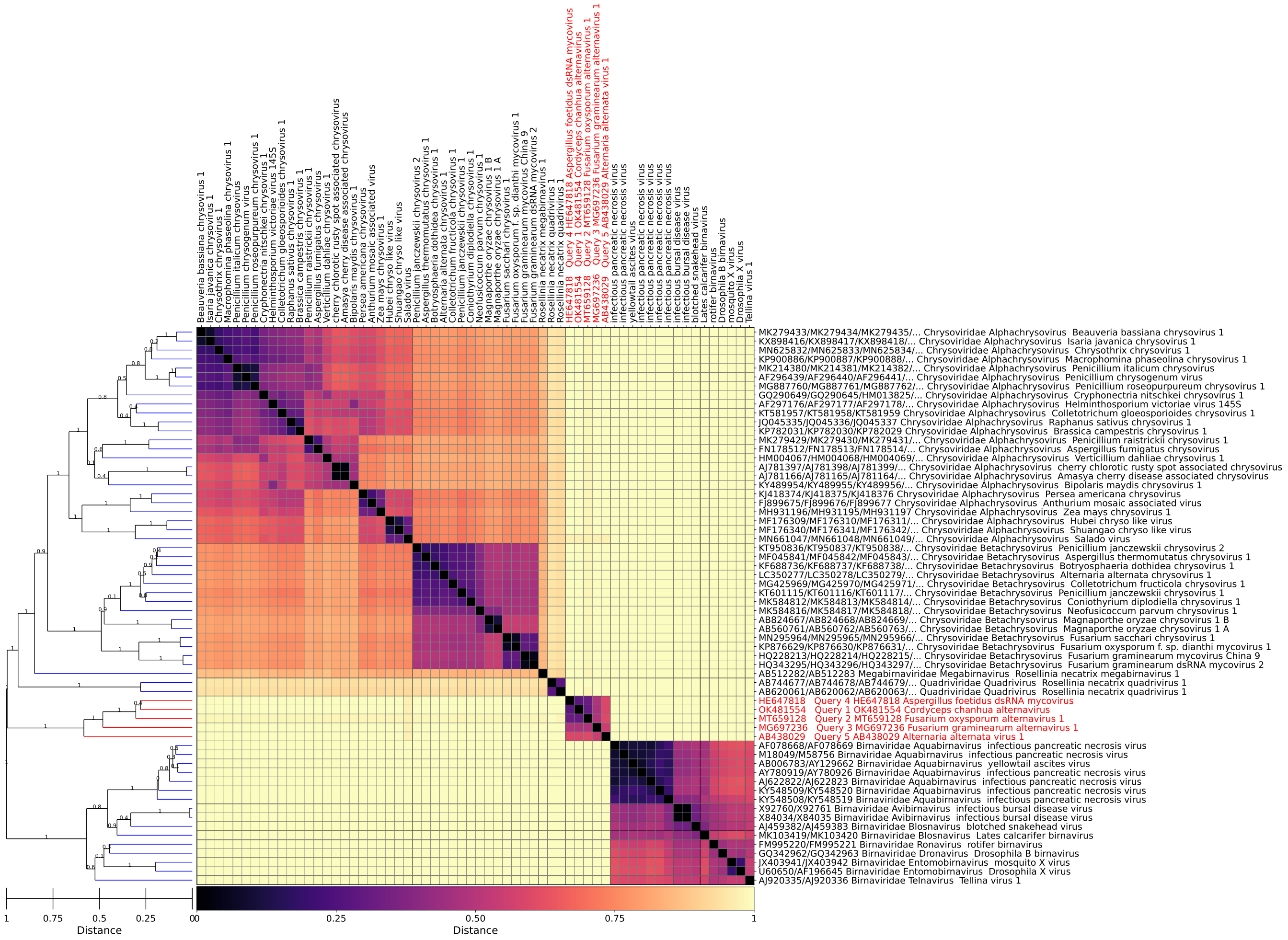

Figure 3: *Alternaviridae*, GRAViTy-V2 heatmap

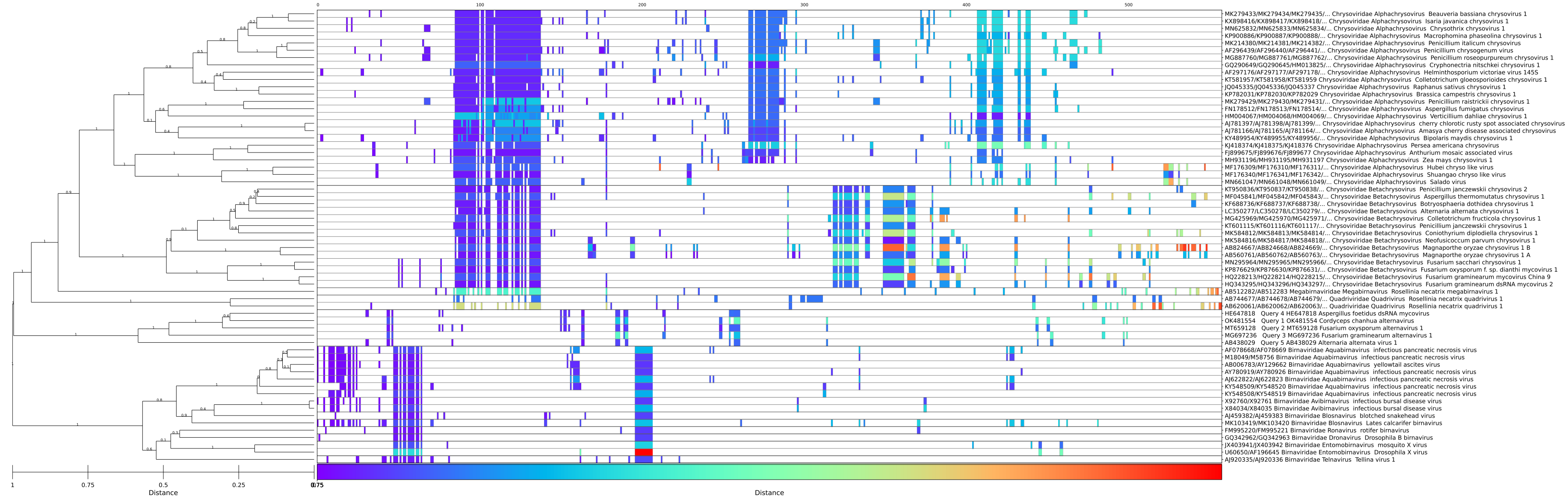

Figure 4: *Alternaviridae*, GRAViTy-V2 barcode

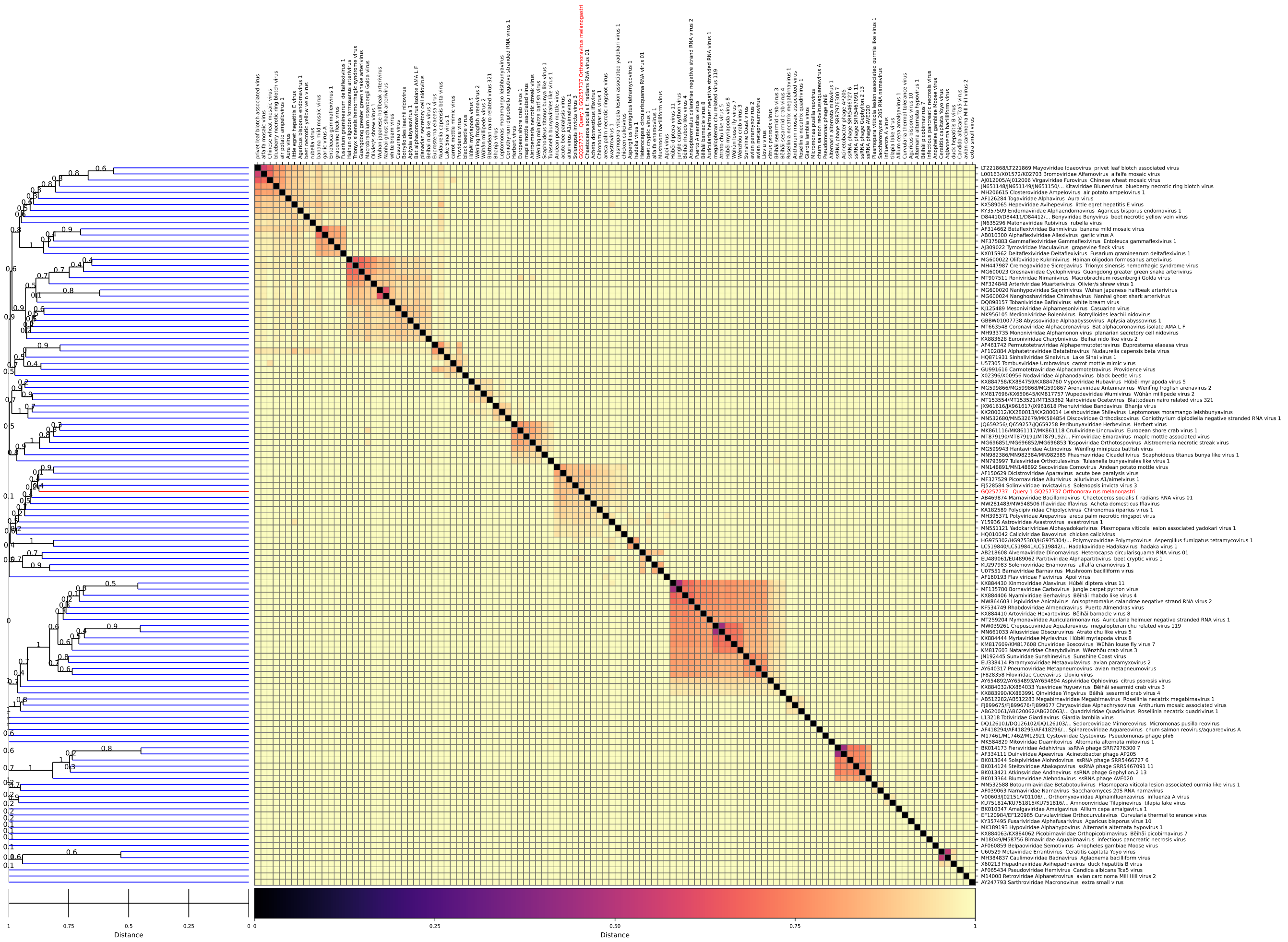

Figure 5: *Picornavirales*, GRAViTy-V2 heatmap

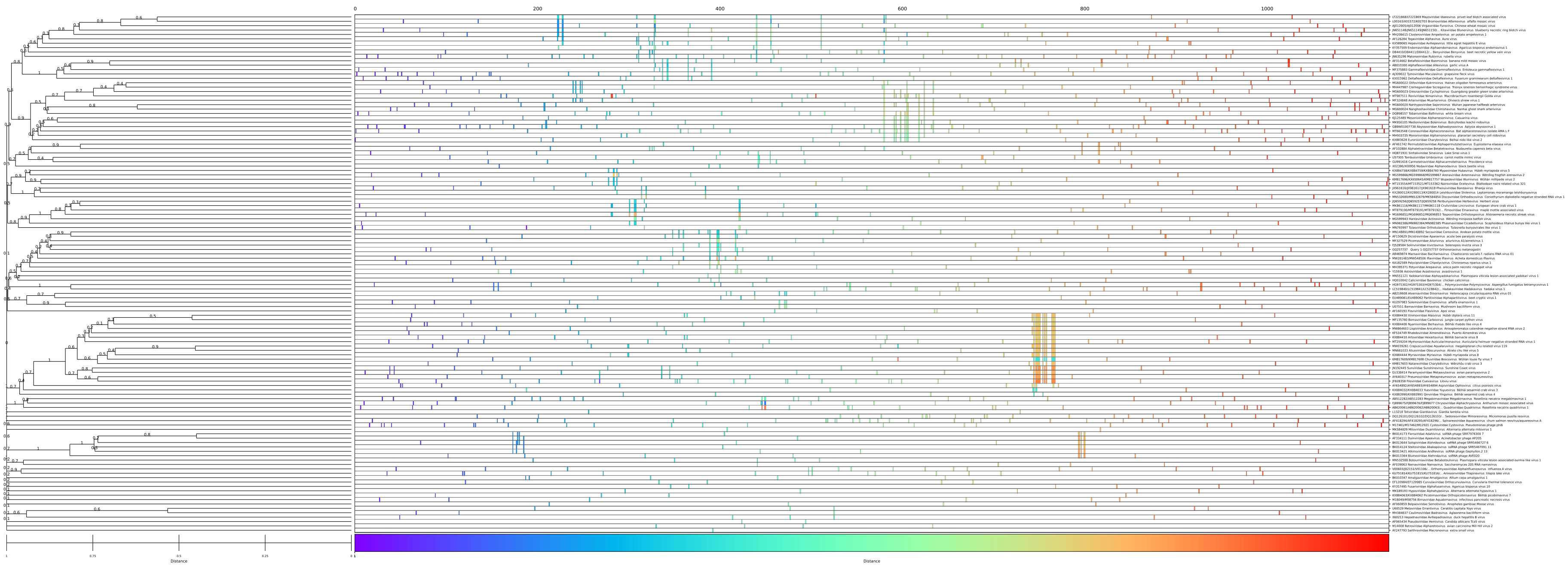

Figure 6: *Picornavirales*, GRAViTy-V2 barcode

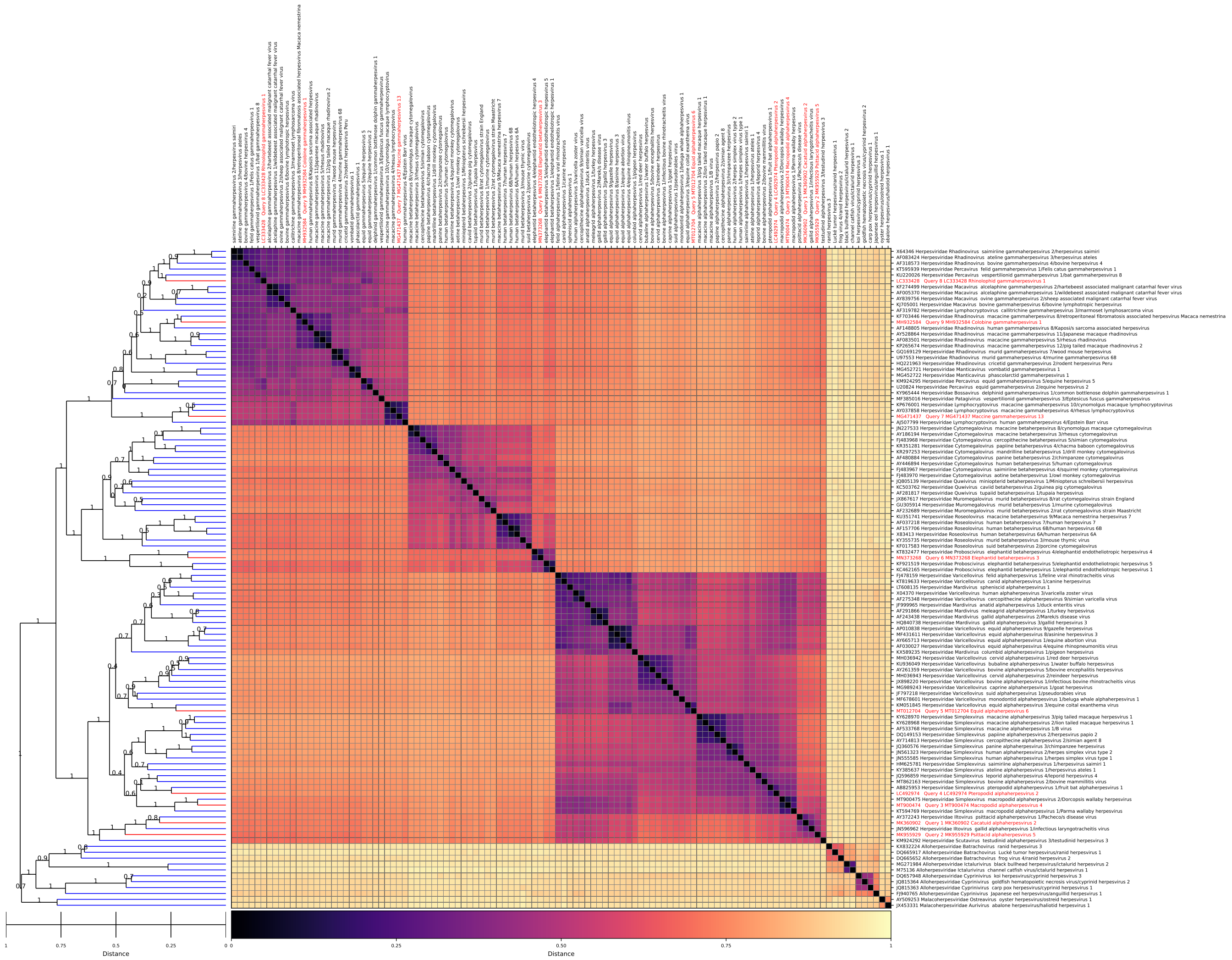

Figure 7: *Herpesvirales*, GRAViTy-V2 heatmap

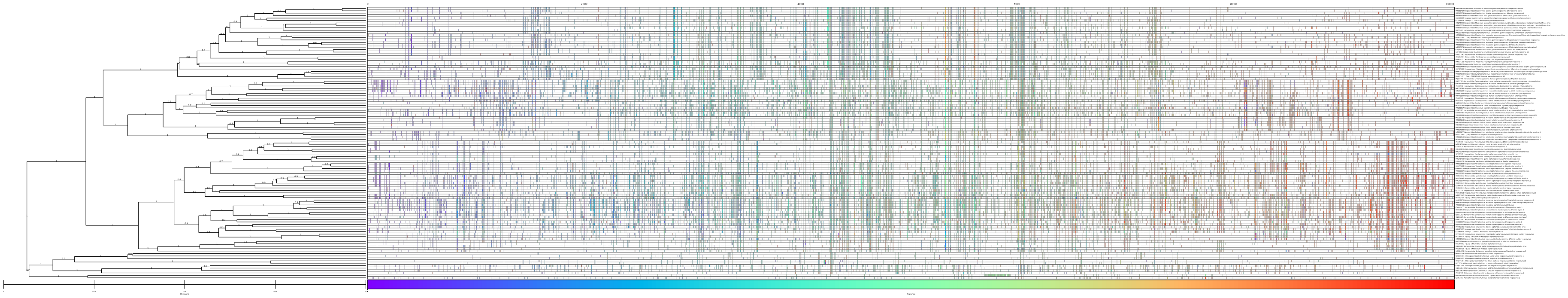

Figure 8: *Herpesvirales*, GRAViTy-V2 barcode

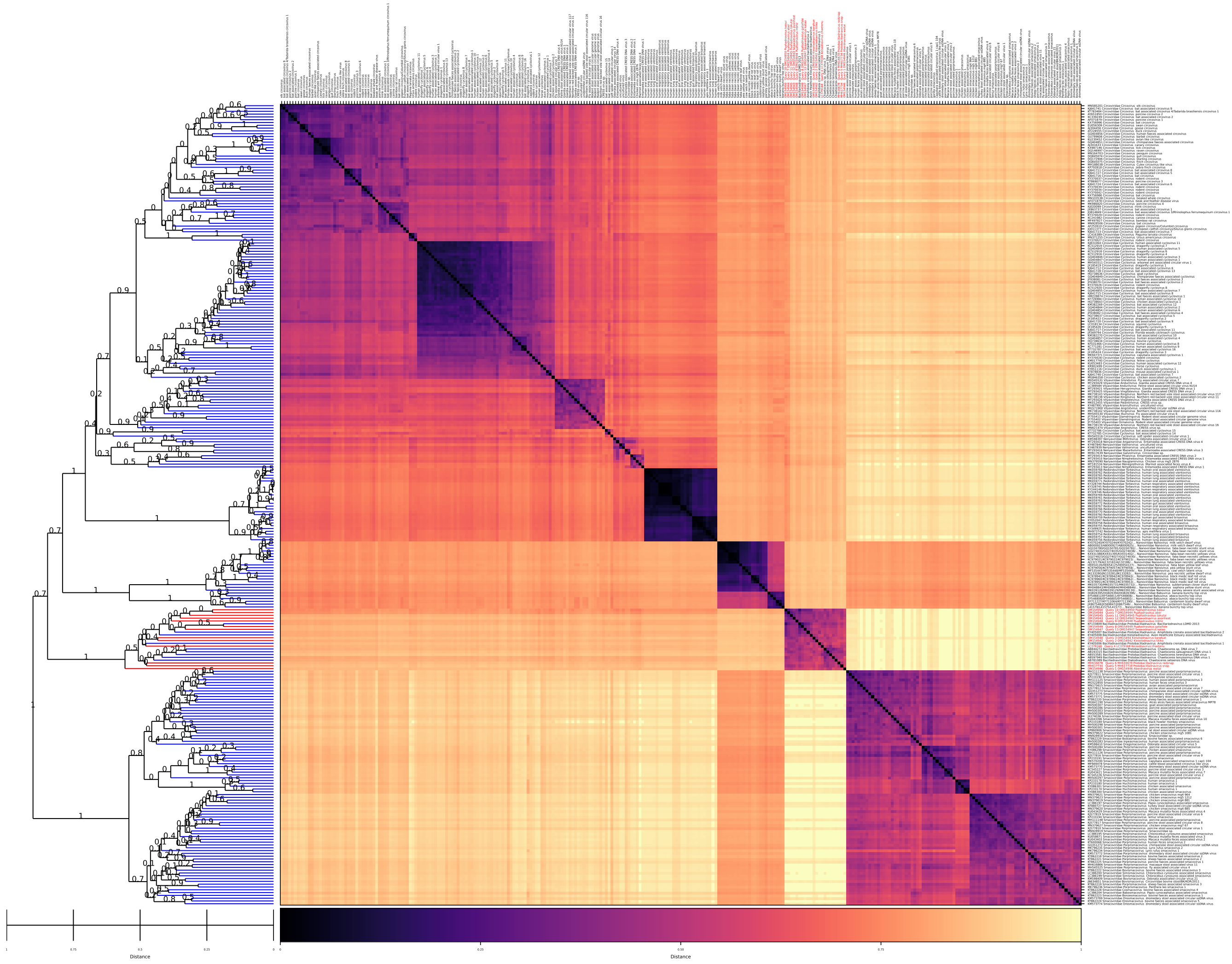

Figure 9: *Bacilladnaviridae*, GRAViTy-V2 heatmap

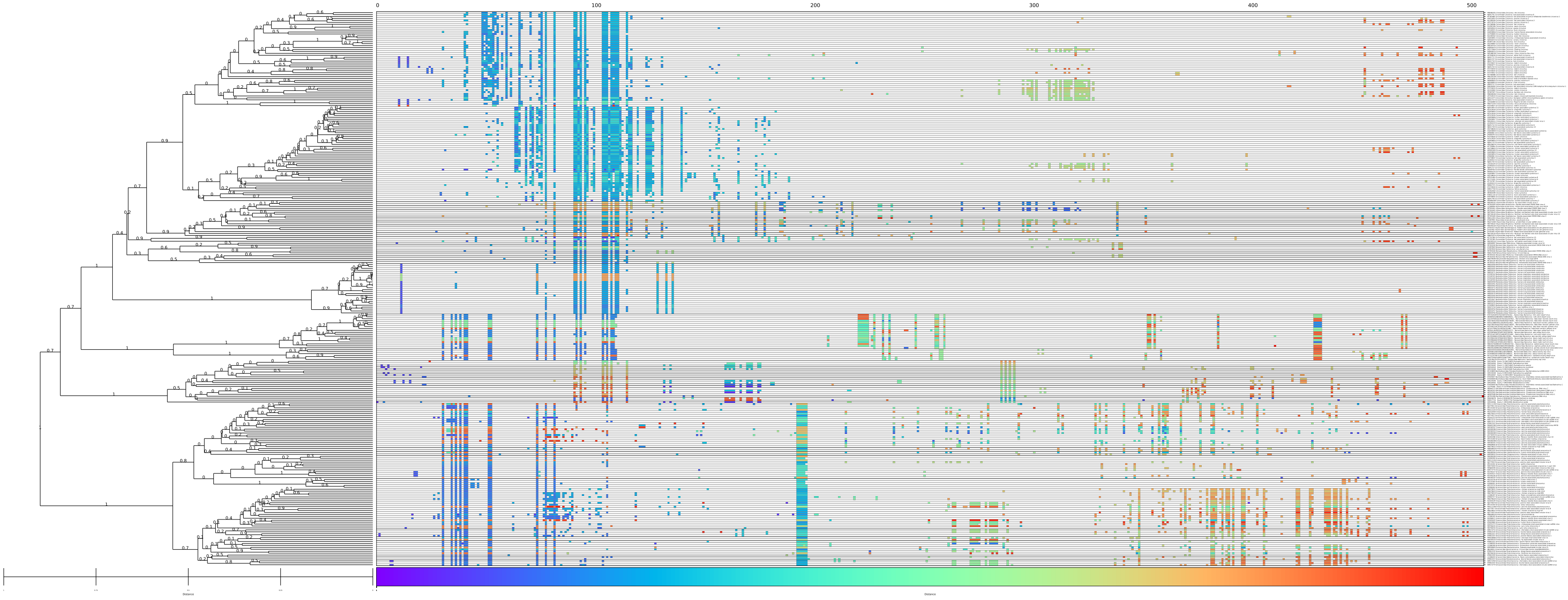

Figure 10: *Bacilladnaviridae*, GRAViTy-V2 barcode

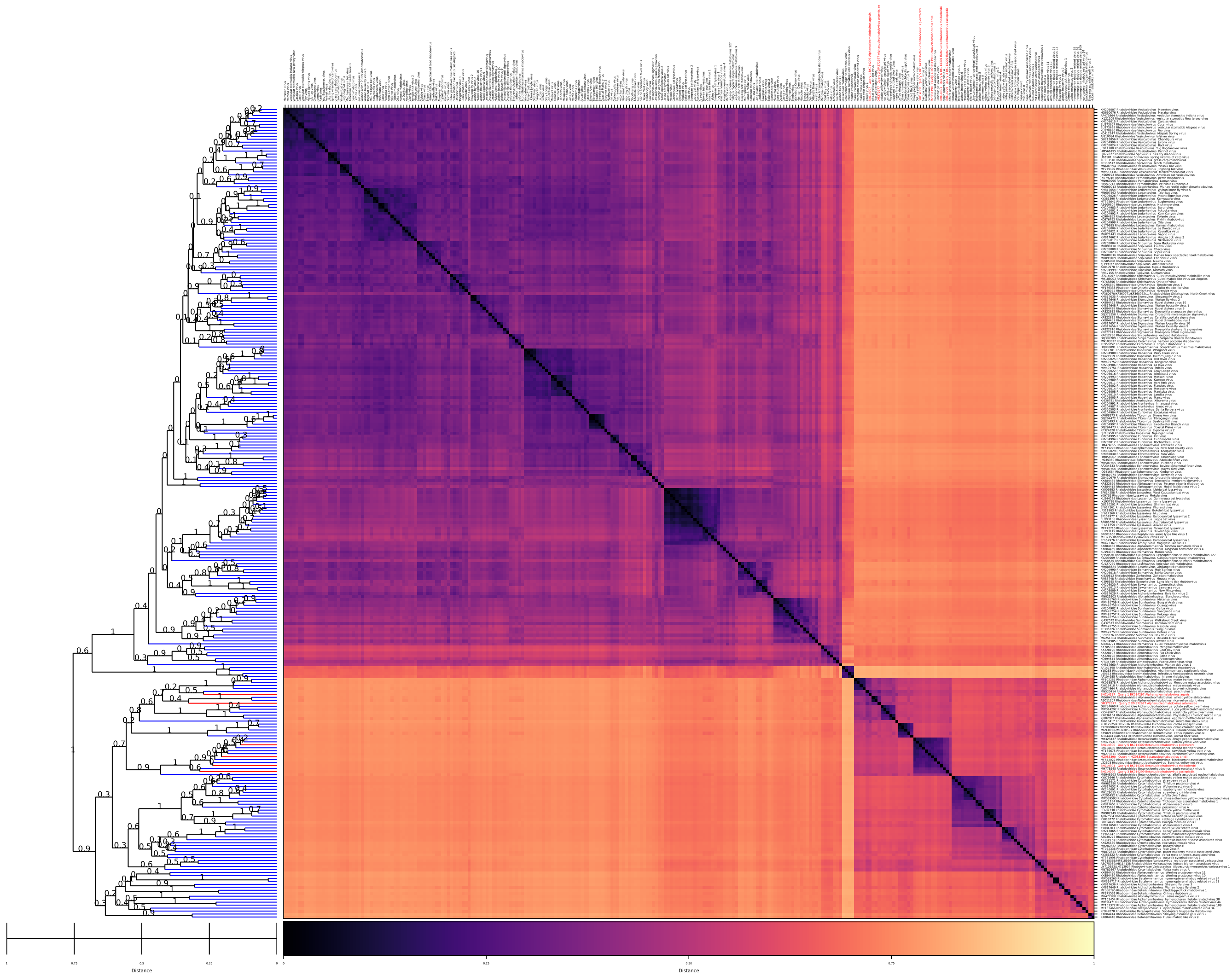

Figure 11: *Alpharhabdovirinae*, GRAViTy-V2 heatmap

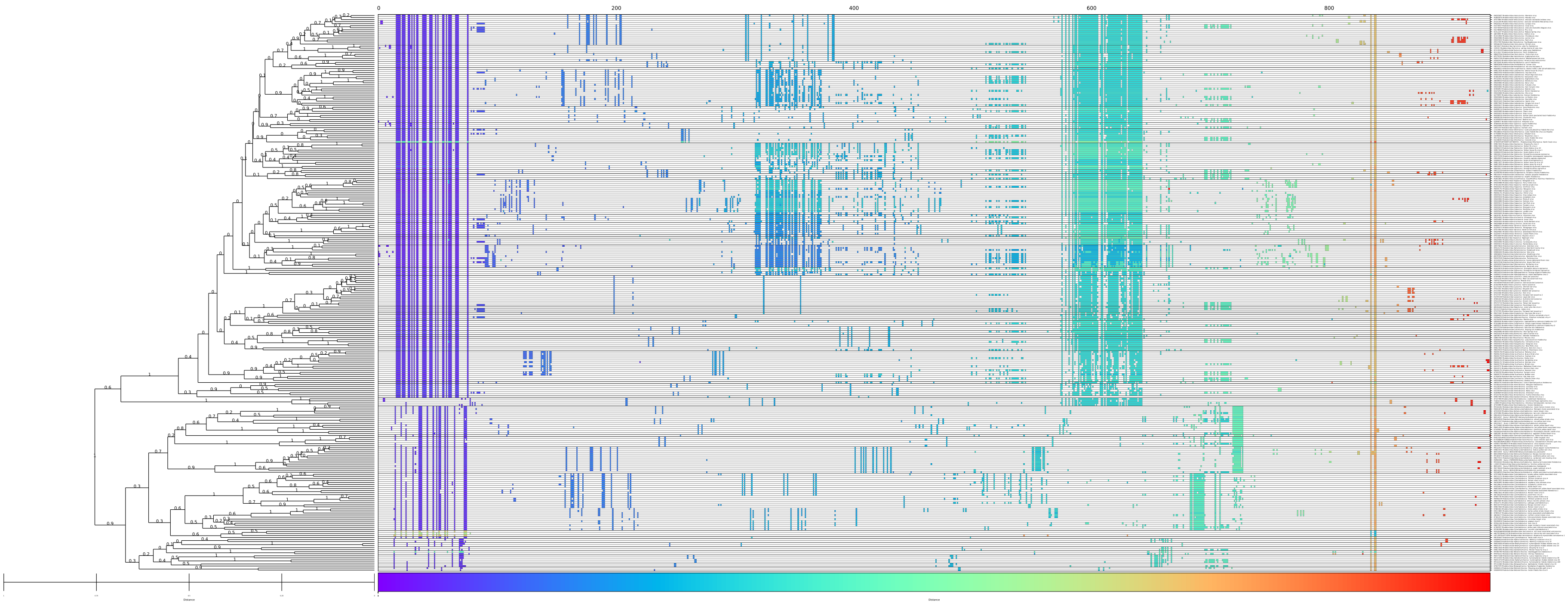

Figure 12: *Alpharhabdovirinae*, GRAViTy-V2 barcode

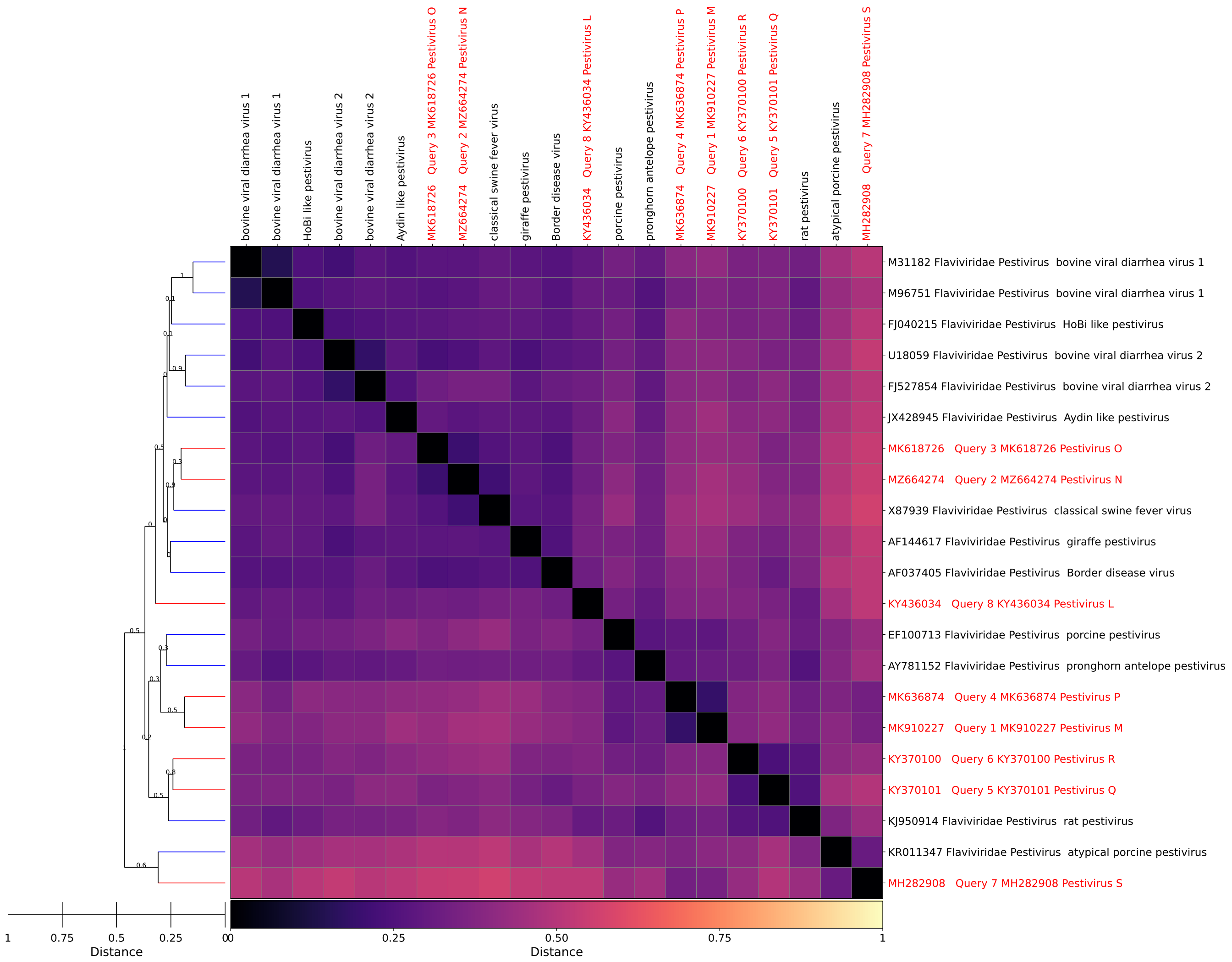

Figure 13: *Pestivirus*, GRAViTy-V2 heatmap

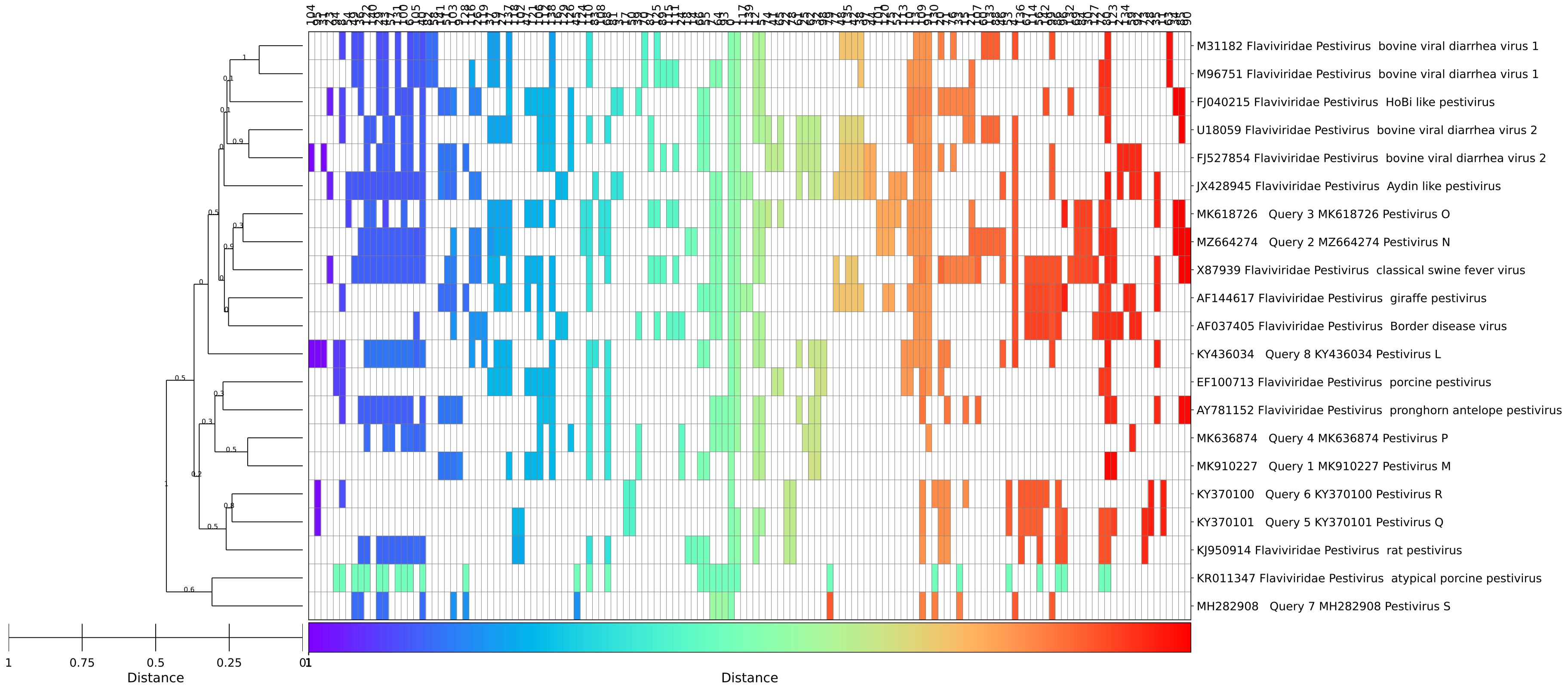

Figure 14: *Pestivirus*, GRAViTy-V2 barcode

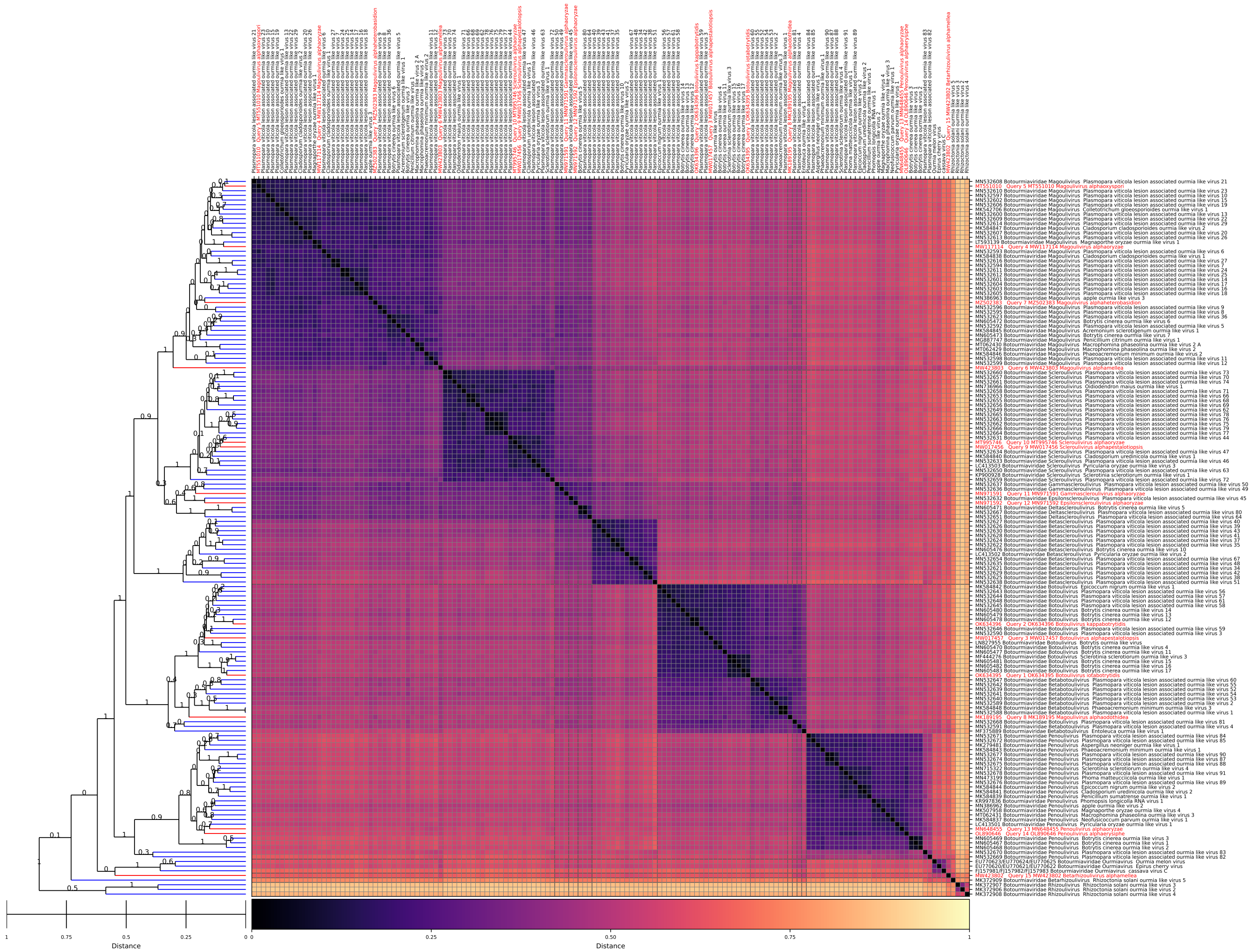

Figure 15: *Botourmiaviridae*, GRAViTy-V2 heatmap

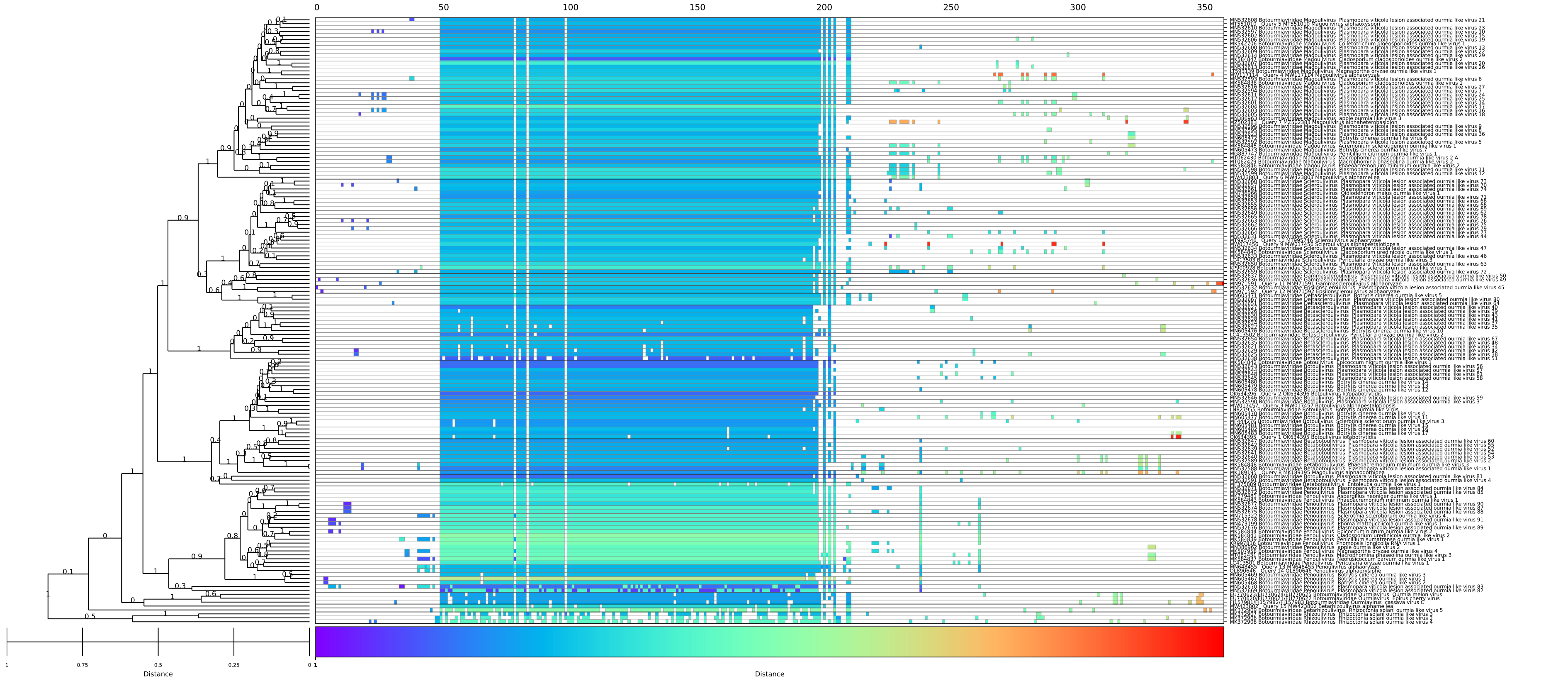

Figure 16: *Botourmiaviridae*, GRAViTy-V2 barcode

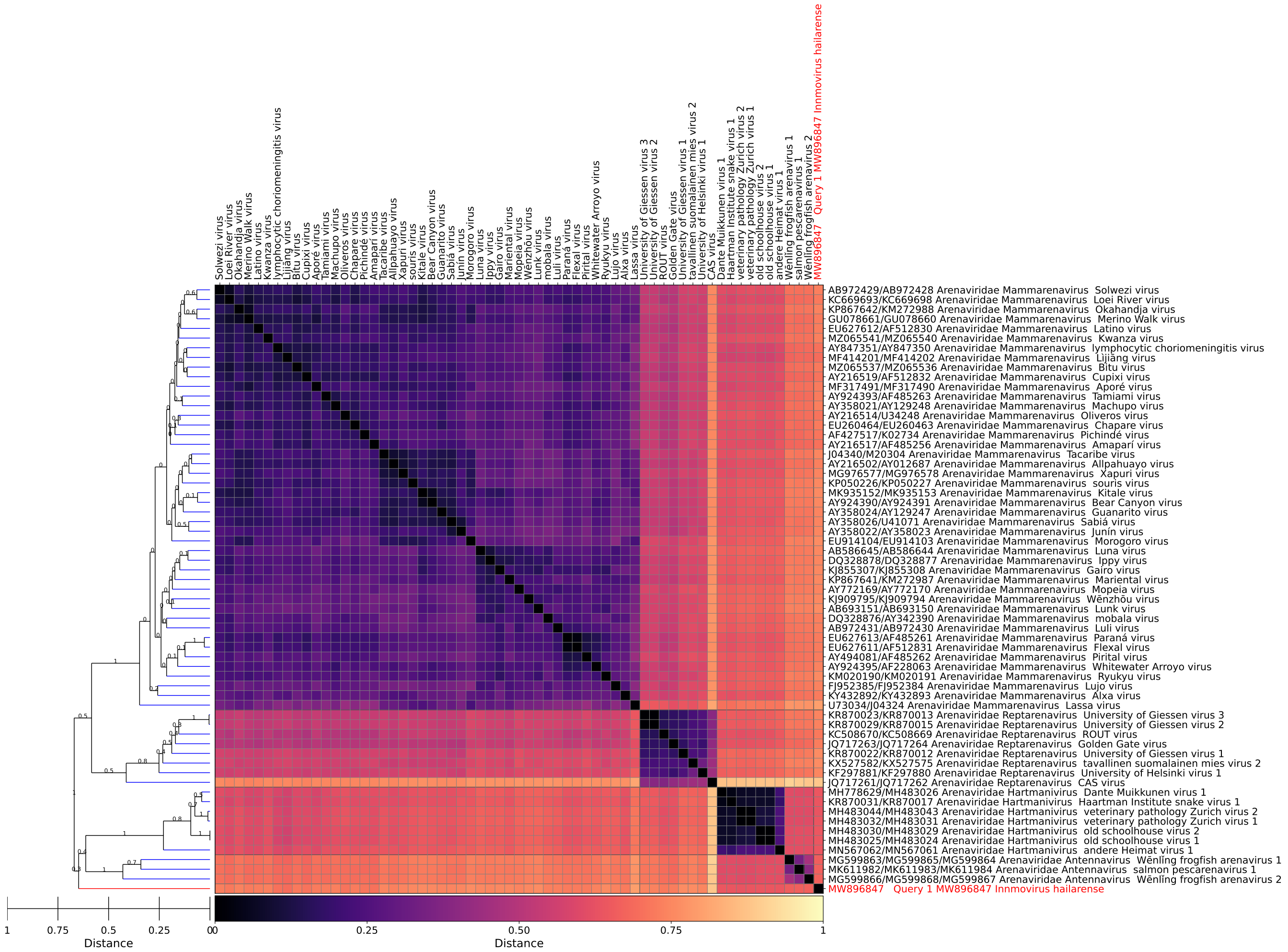

Figure 17: *Arenaviridae*, GRAViTy-V2 heatmap

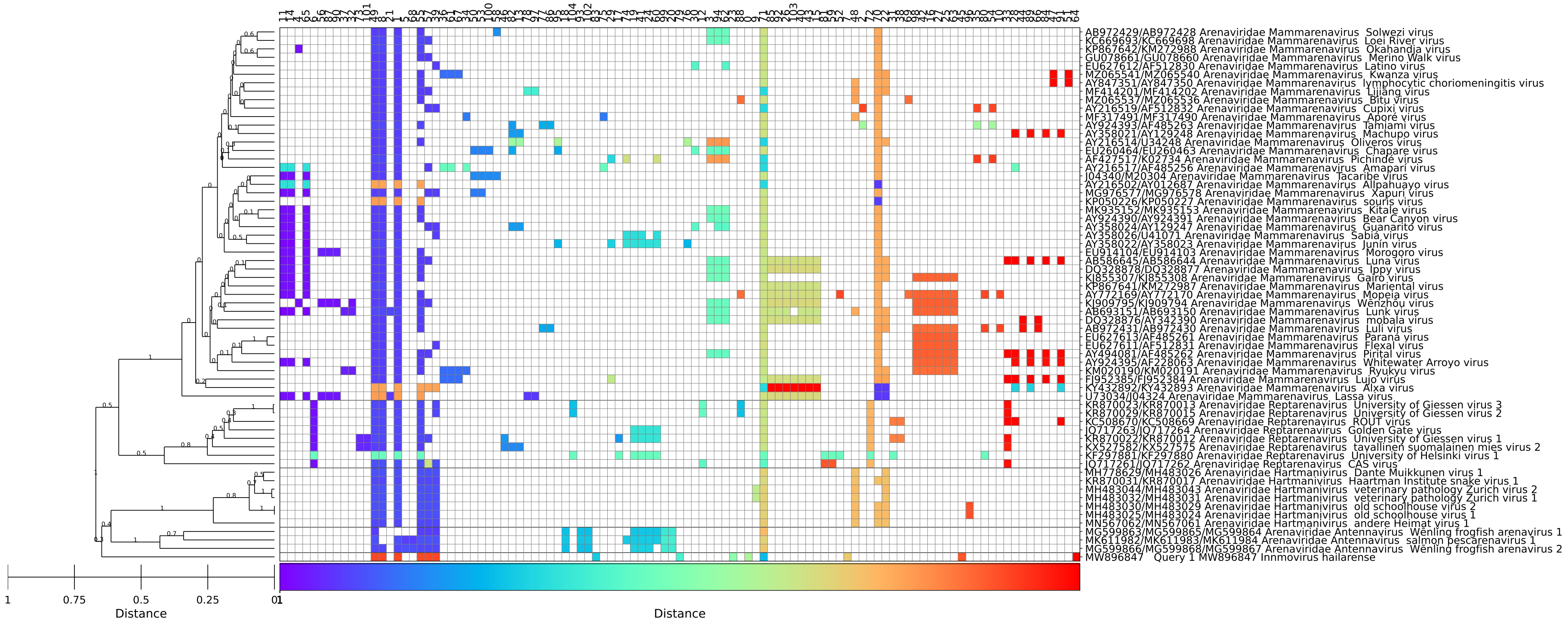

Figure 18: *Arenaviridae*, GRAViTy-V2 barcode

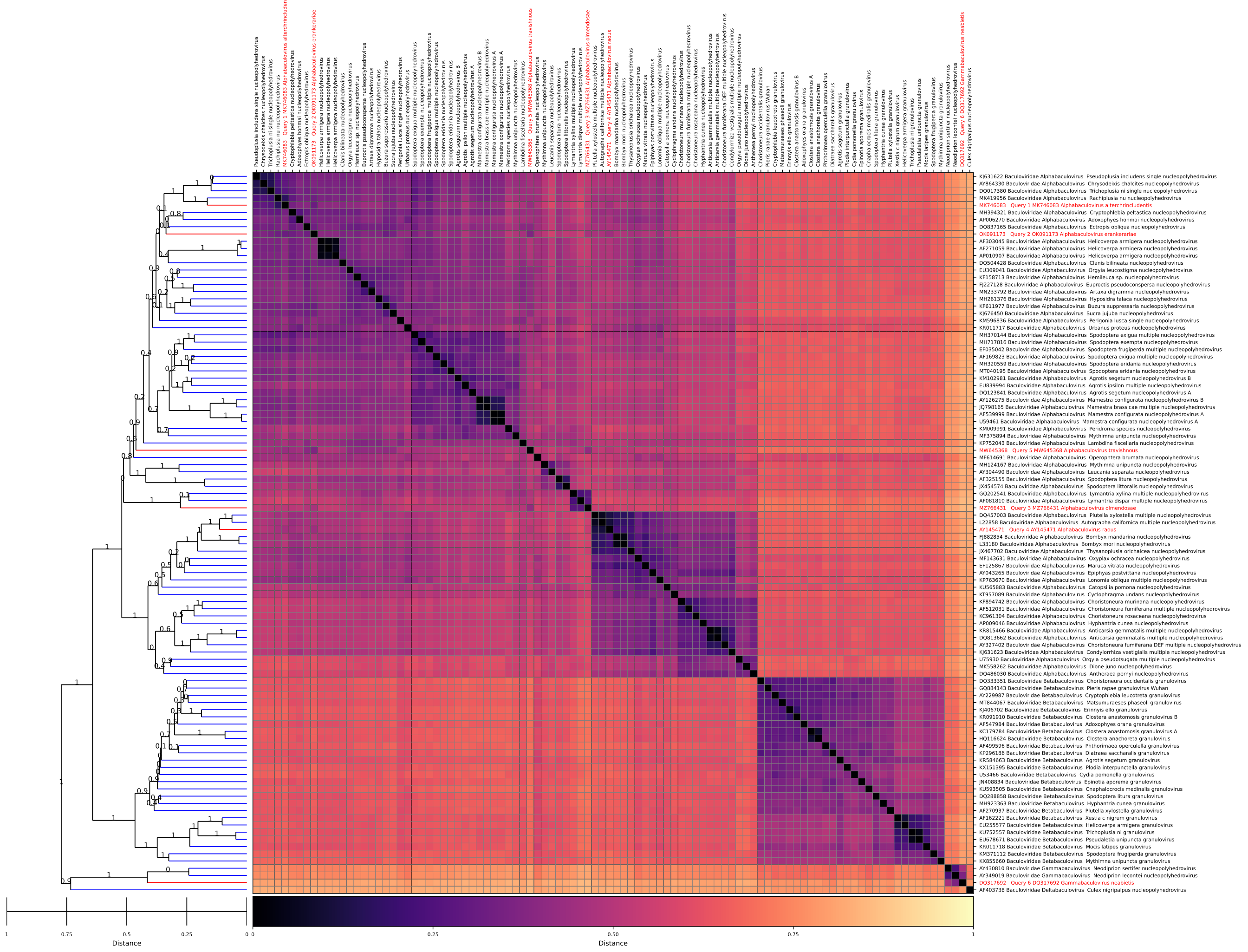

Figure 19: *Baculoviridae*, GRAViTy-V2 heatmap

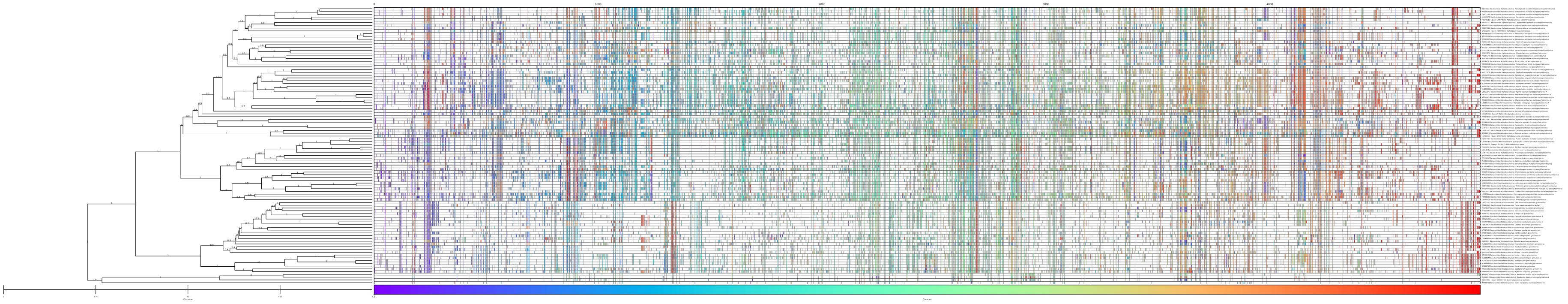

Figure 20: *Baculoviridae*, GRAVITy-V2 barcode

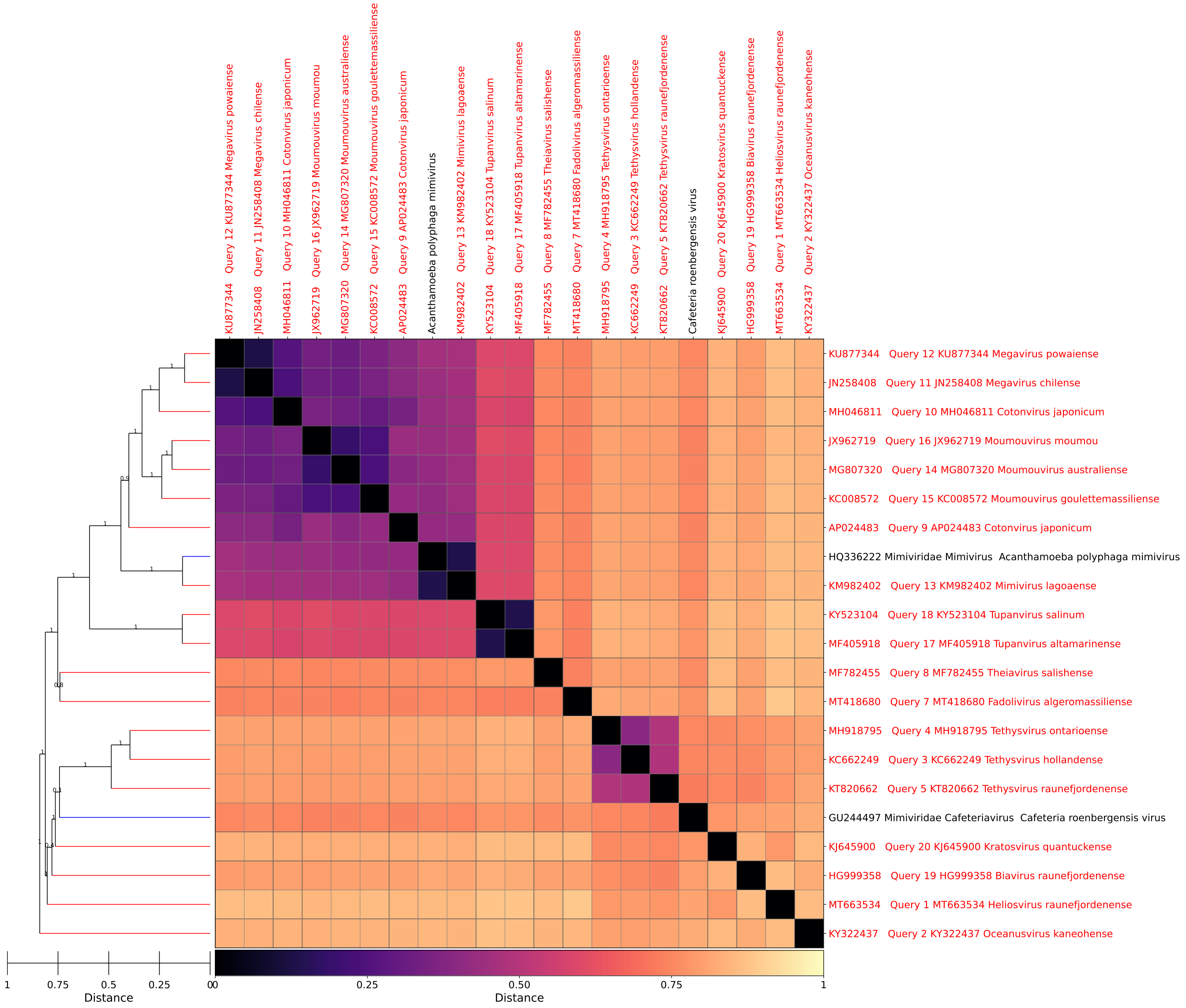

Figure 21: *Imitervirales*, GRAViTy-V2 heatmap

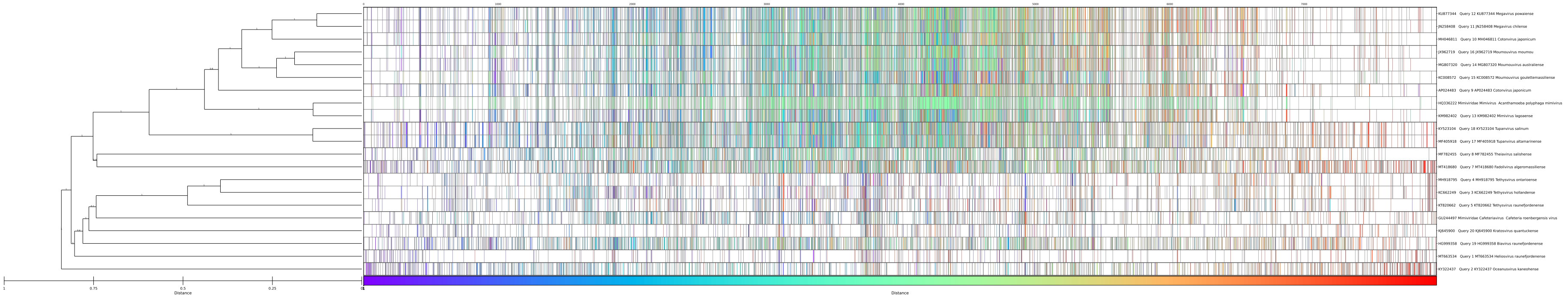

Figure 22: *Imitervirales*, GRAViTy-V2 barcode

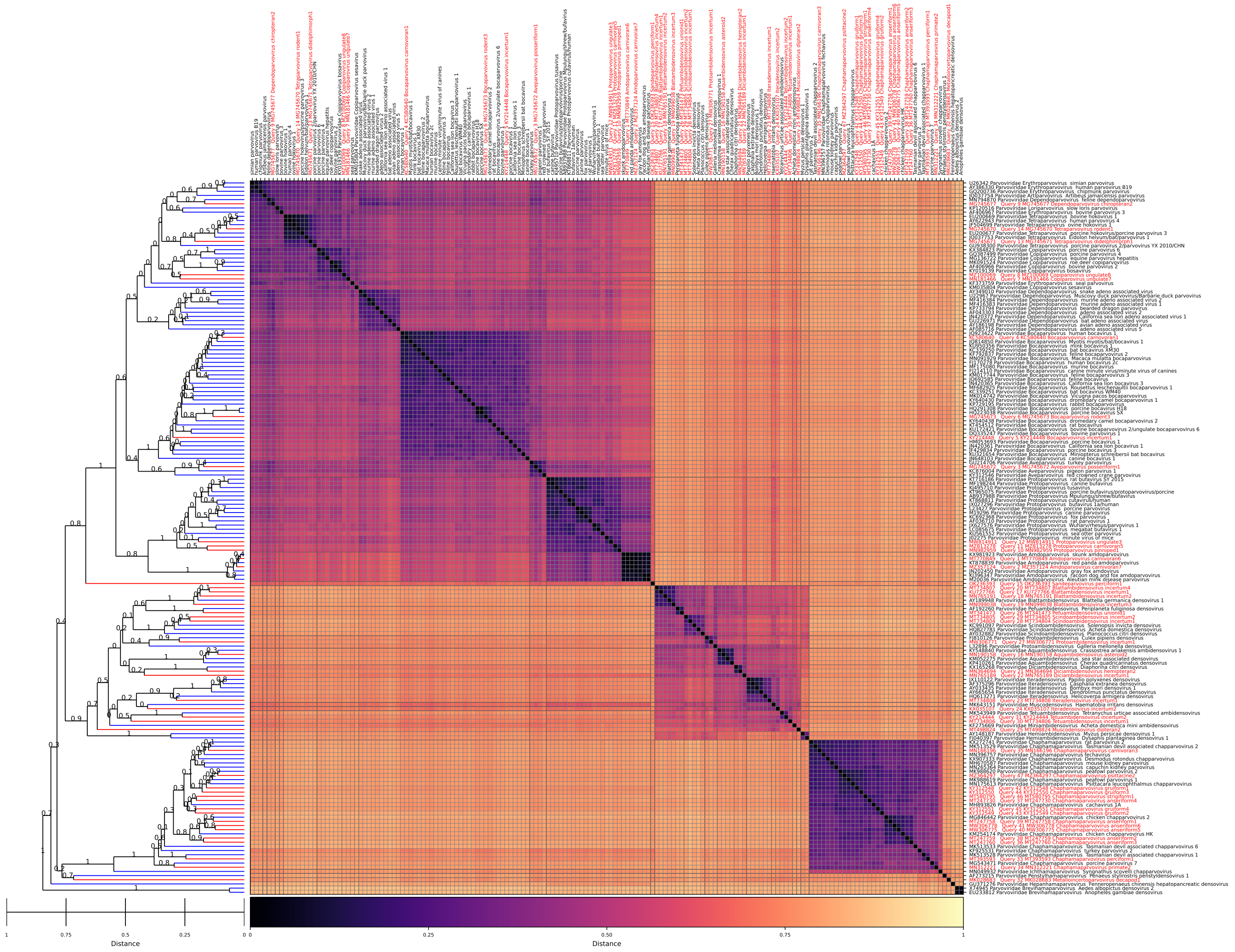

Figure 23: *Parvoviridae*, GRAViTy-V2 heatmap

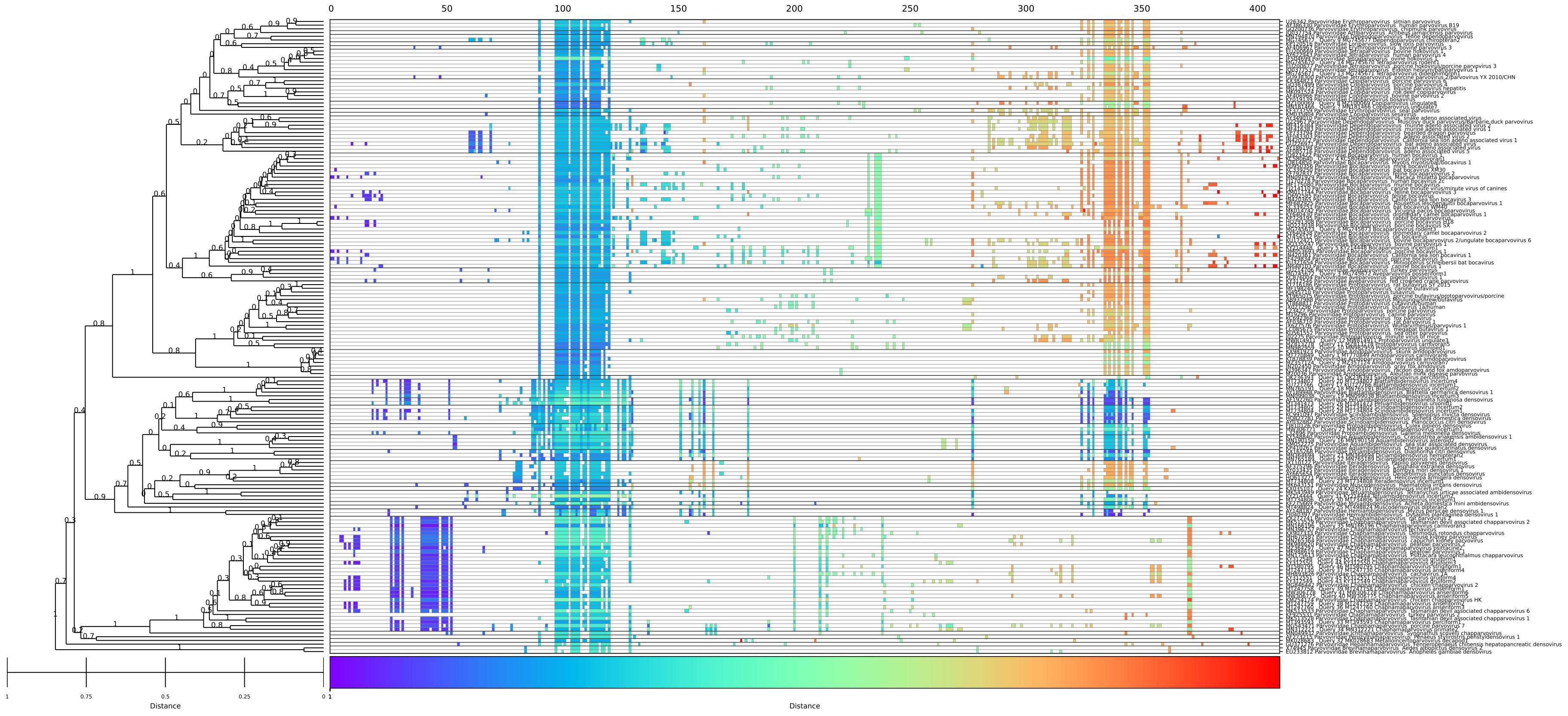

Figure 24: *Parvoviridae*, GRAViTy-V2 barcode

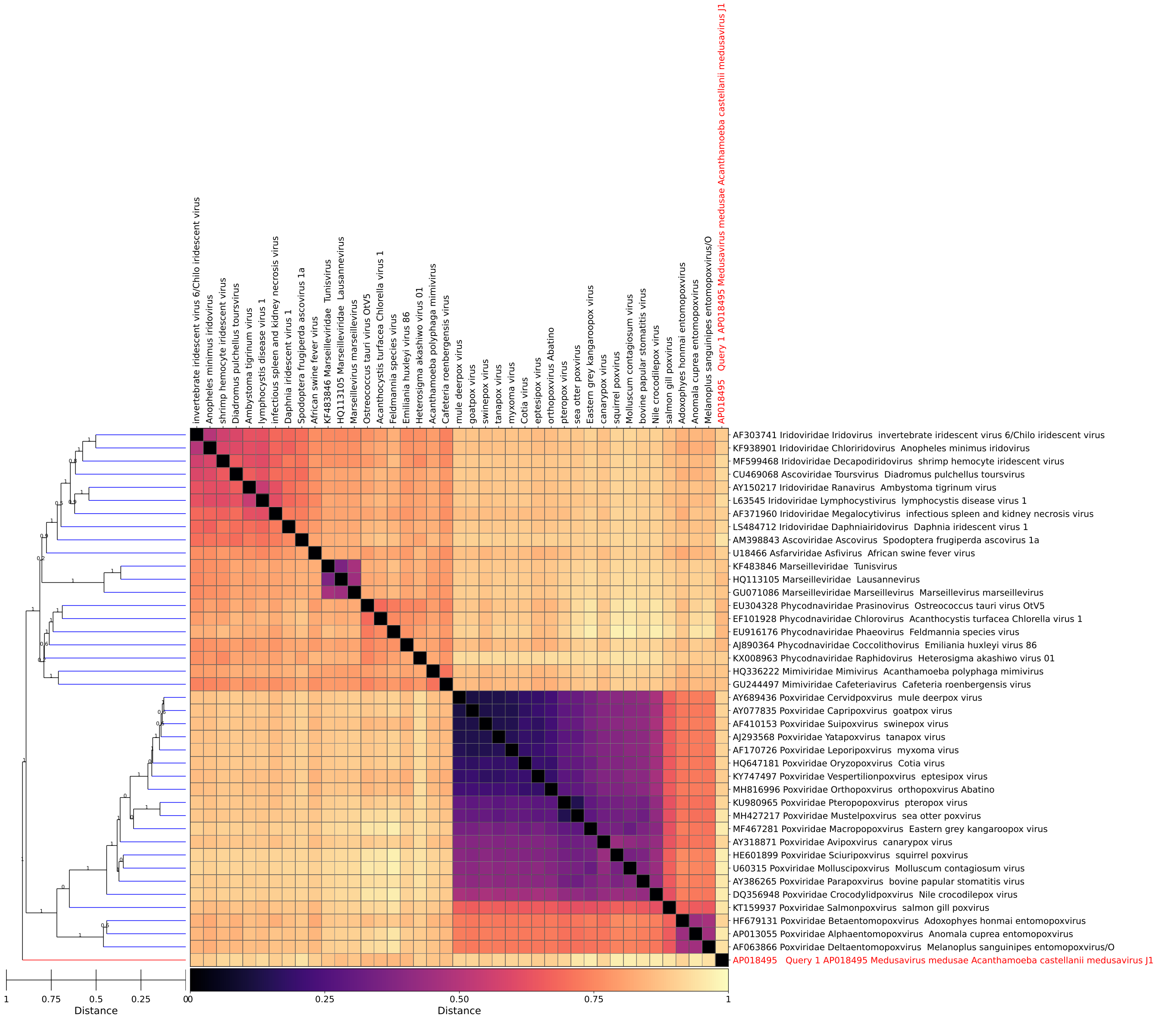

Figure 25: *Mamonoviridae*, GRAViTy-V2 heatmap

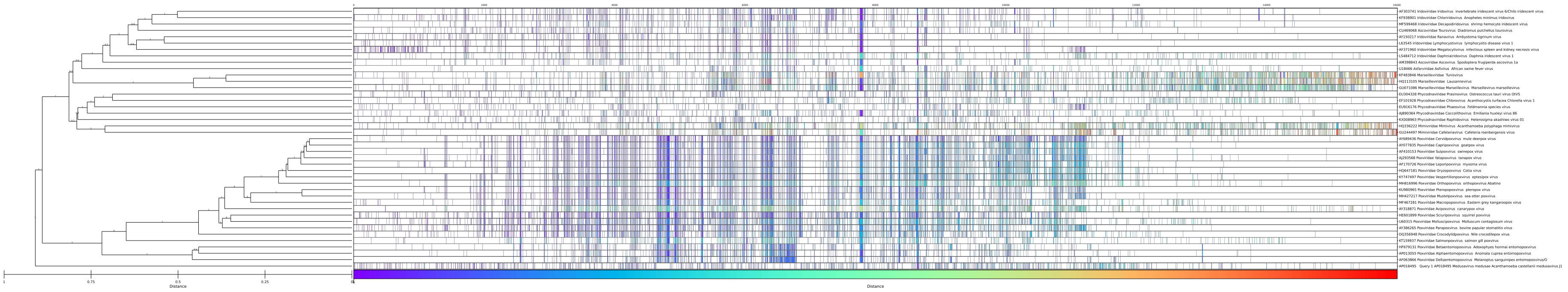

Figure 26: *Mamonoviridae*, GRAViTy-V2 barcode

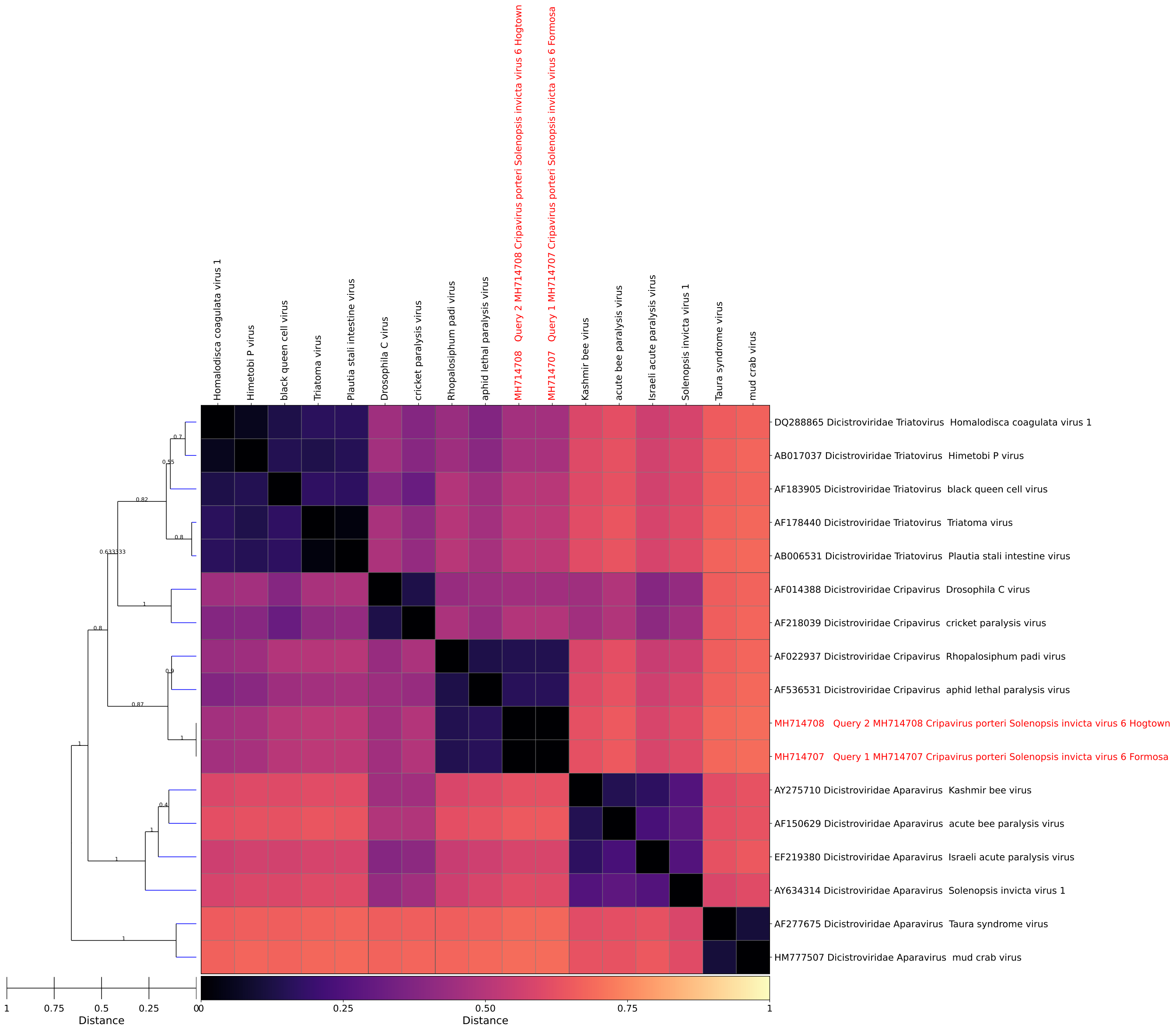

Figure 27: *Crispavirus*, GRAViTy-V2 heatmap

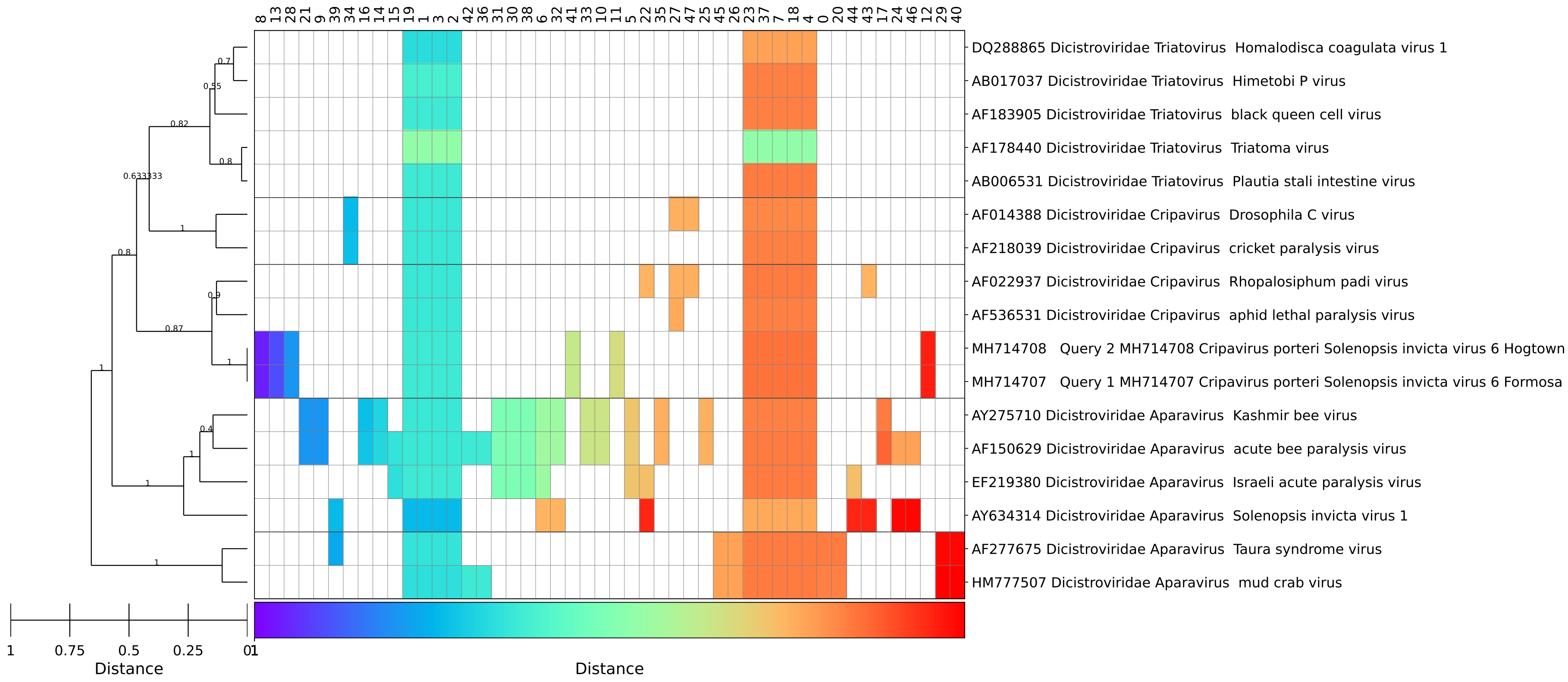

Figure 28: *Crispavirus*, GRAViTy-V2 barcode

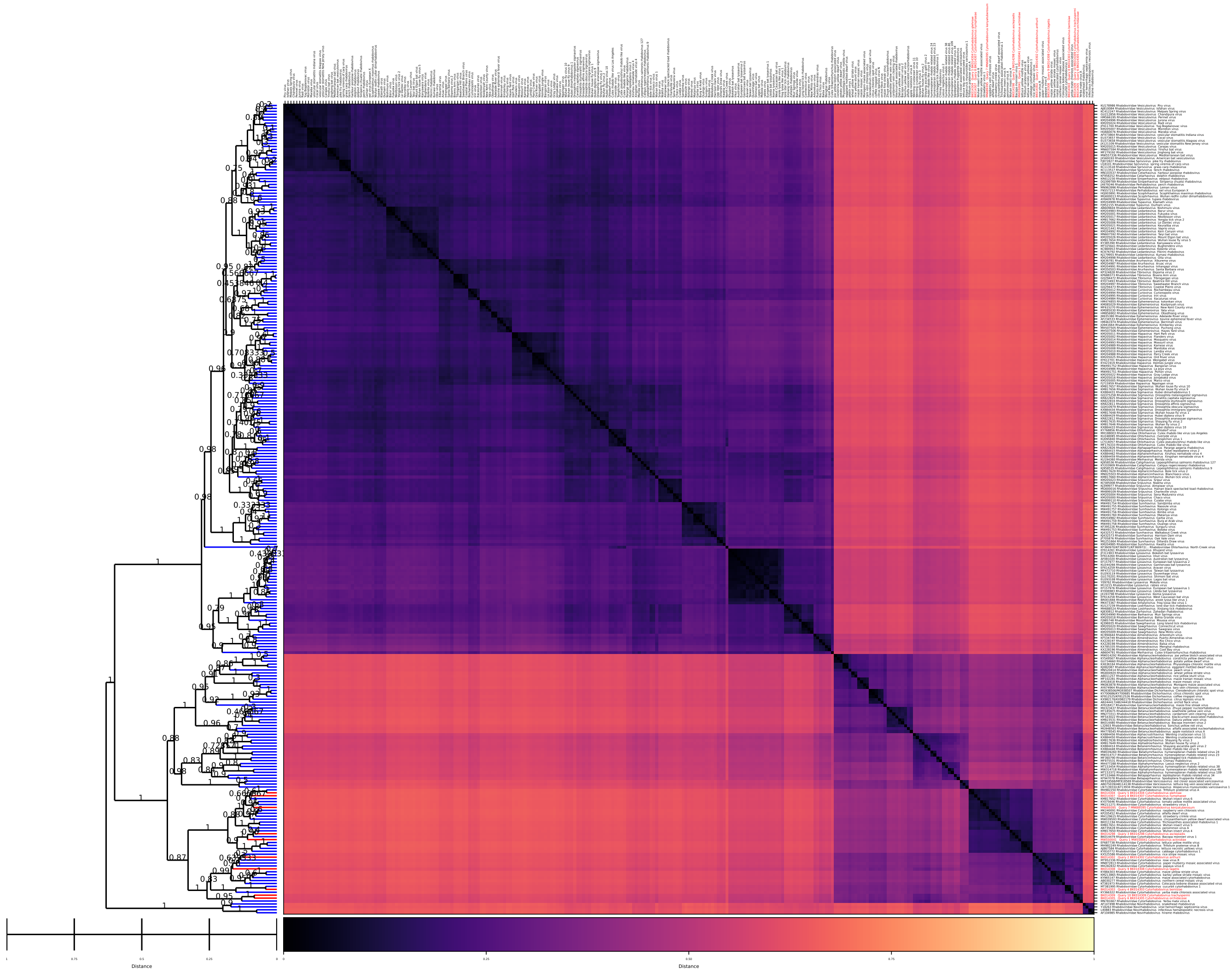

Figure 29: *Cytorhabdovirus*, GRAViTy-V2 heatmap

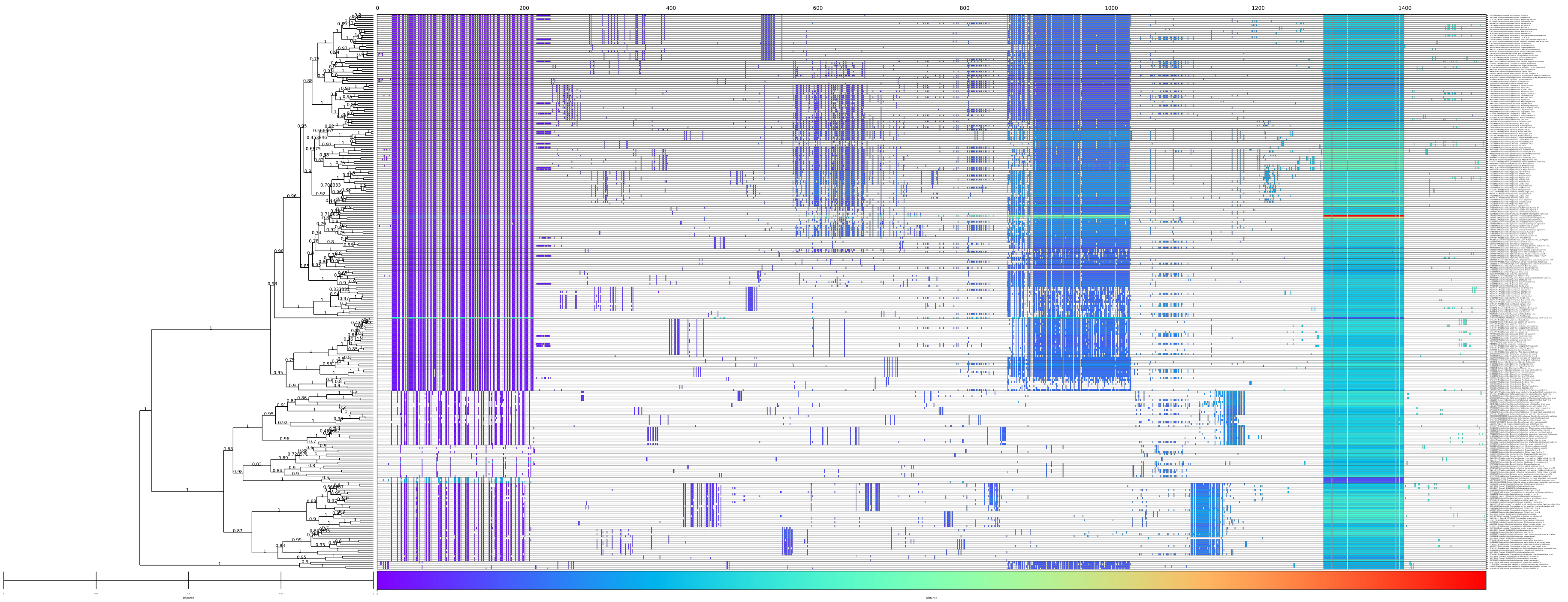

Figure 30: *Cytorhabdovirus*, GRAViTy-V2 barcode

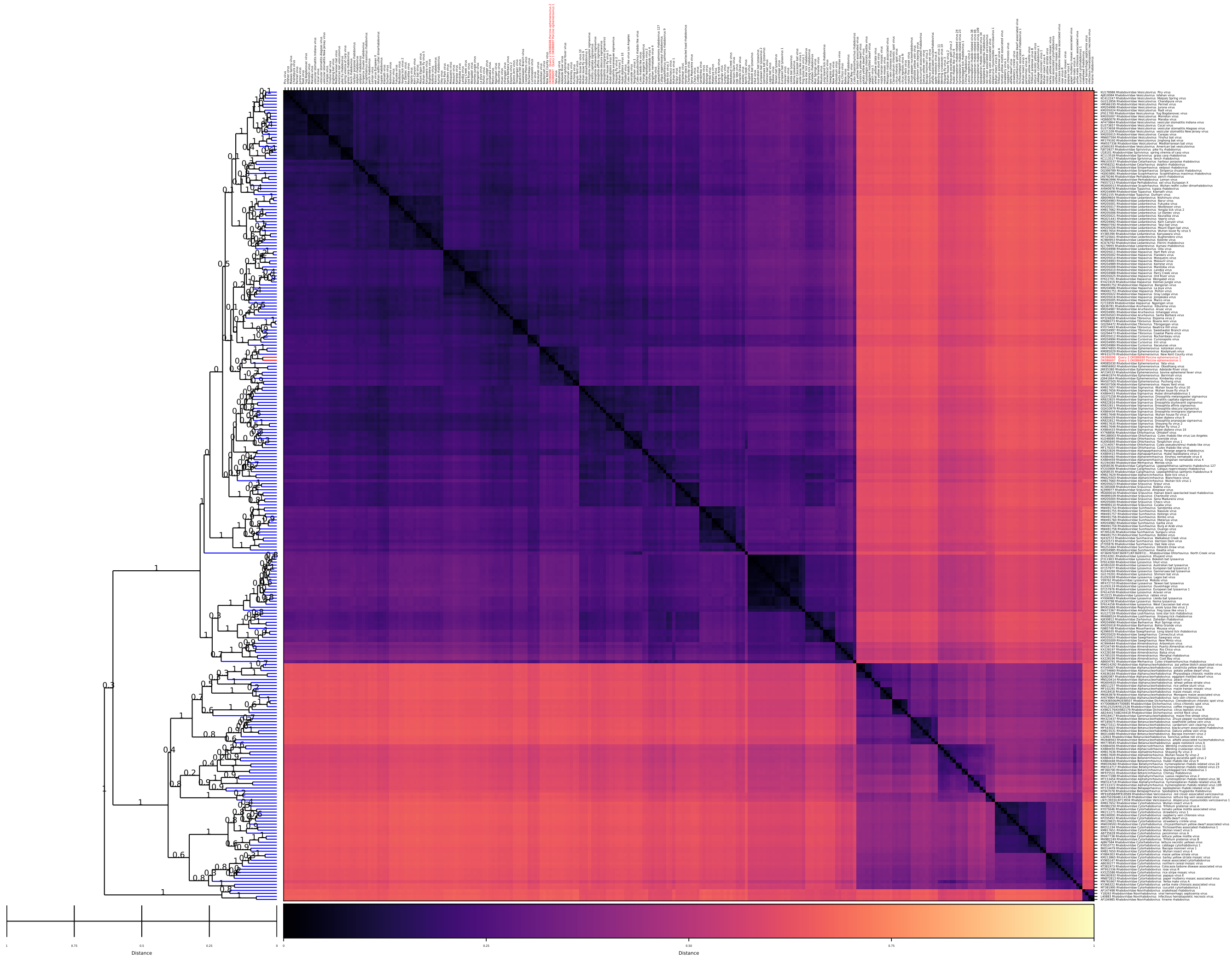

Figure 31: *Ephemerovirus*, GRAViTy-V2 heatmap

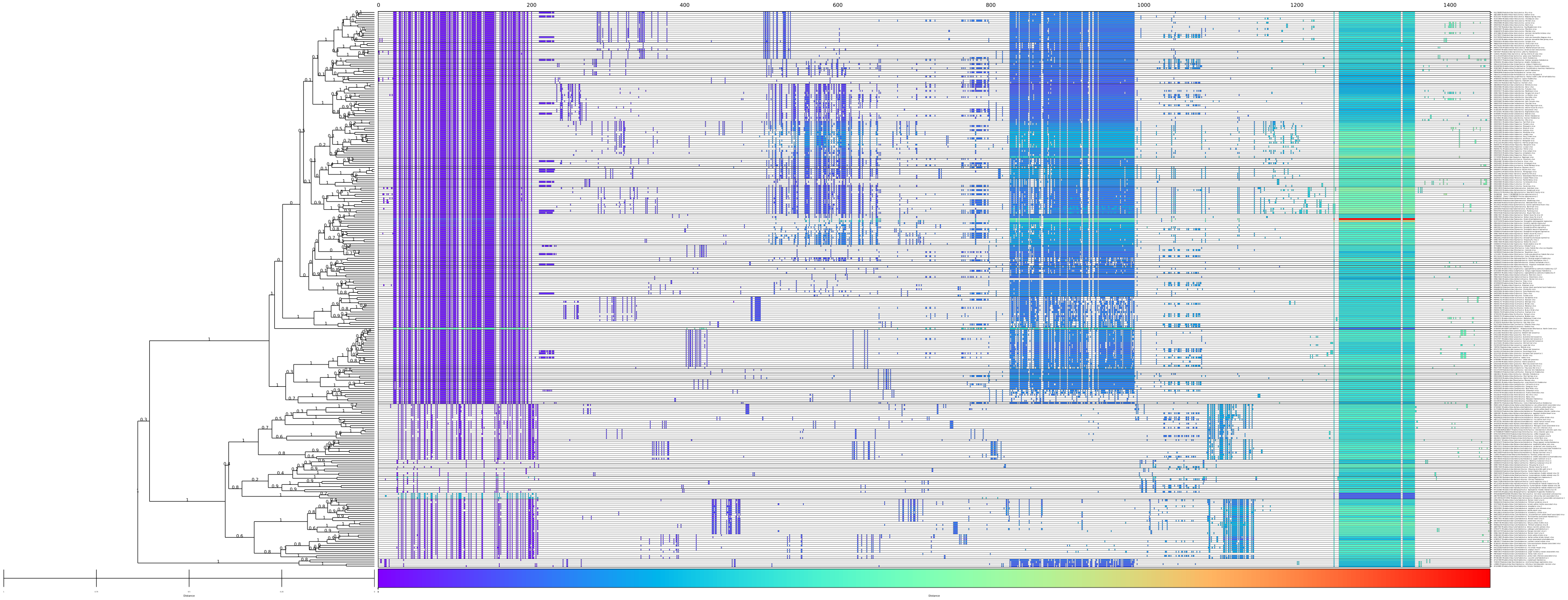

Figure 32: *Ephemerovirus*, GRAViTy-V2 barcode

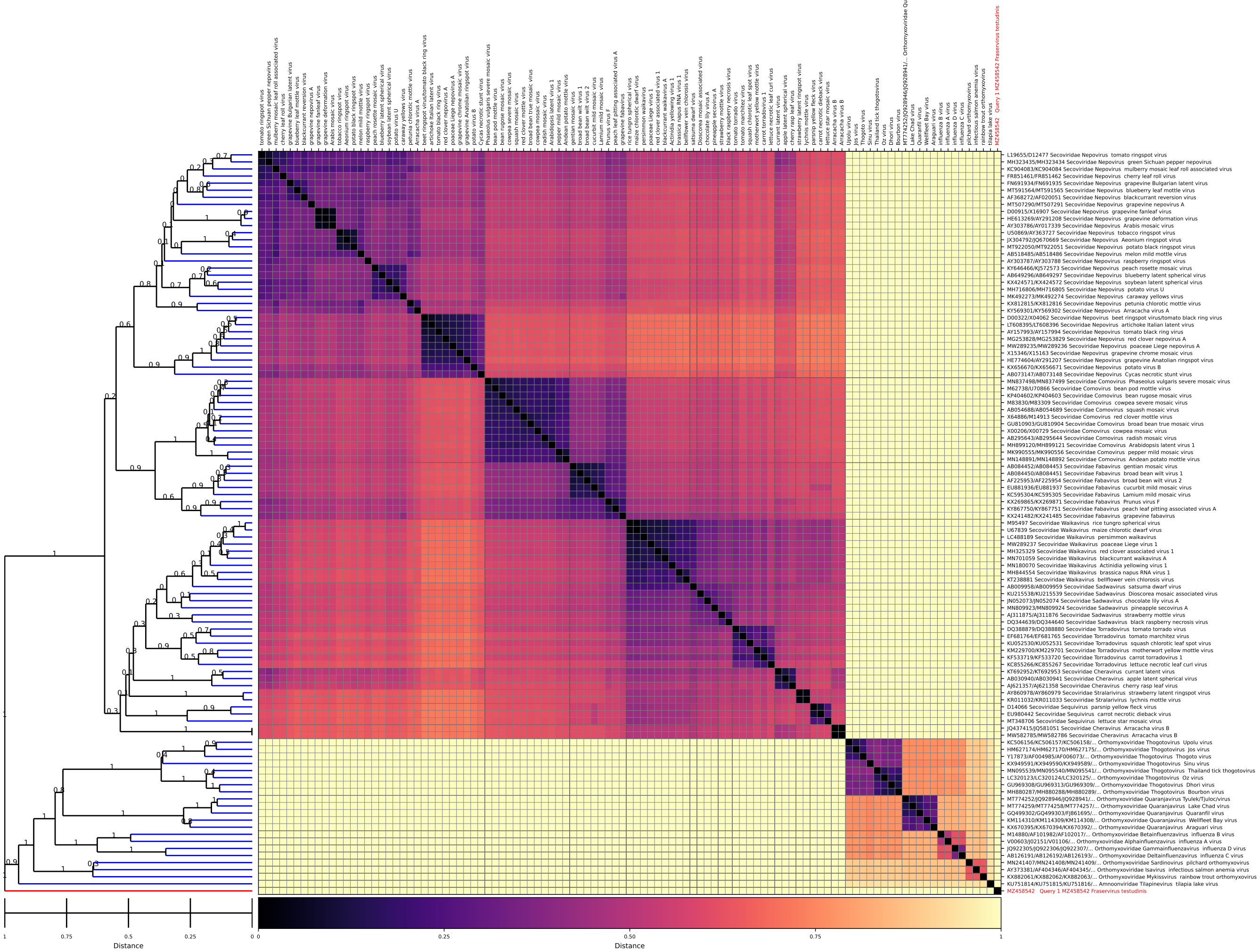

Figure 33: *Fraservirus*, GRAViTy-V2 heatmap

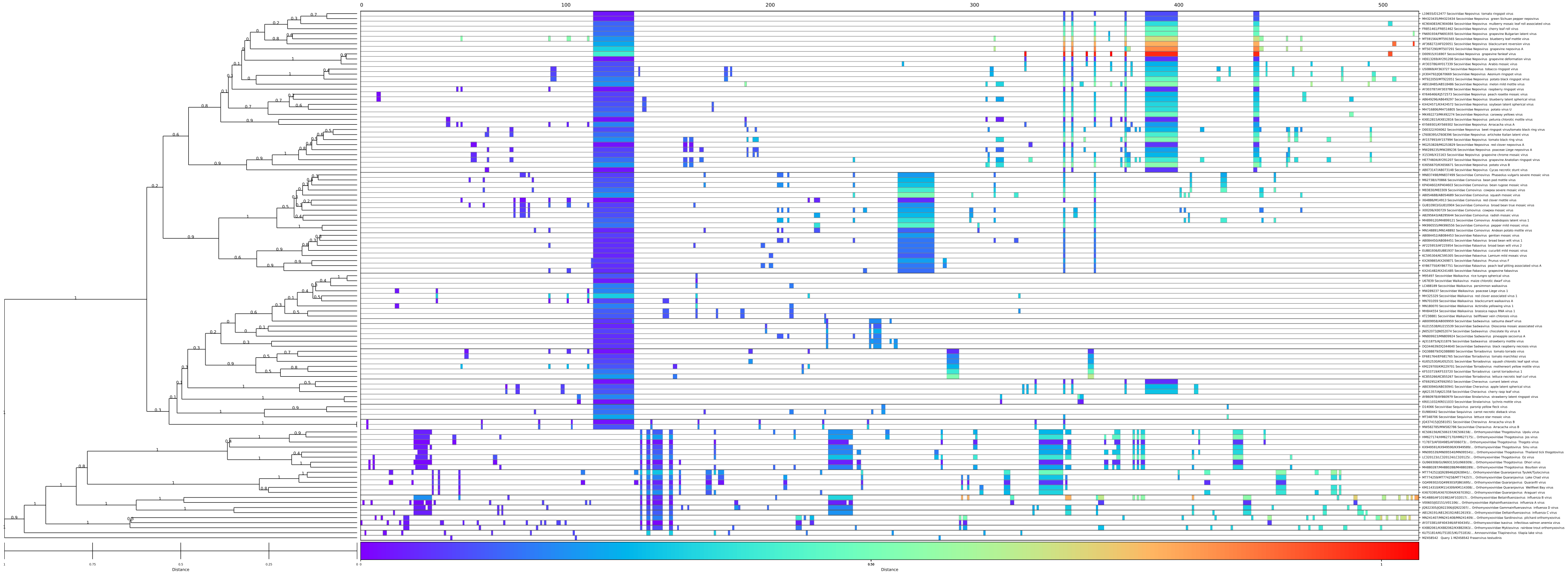

Figure 34: *Fraservirus*, GRAViTy-V2 barcode

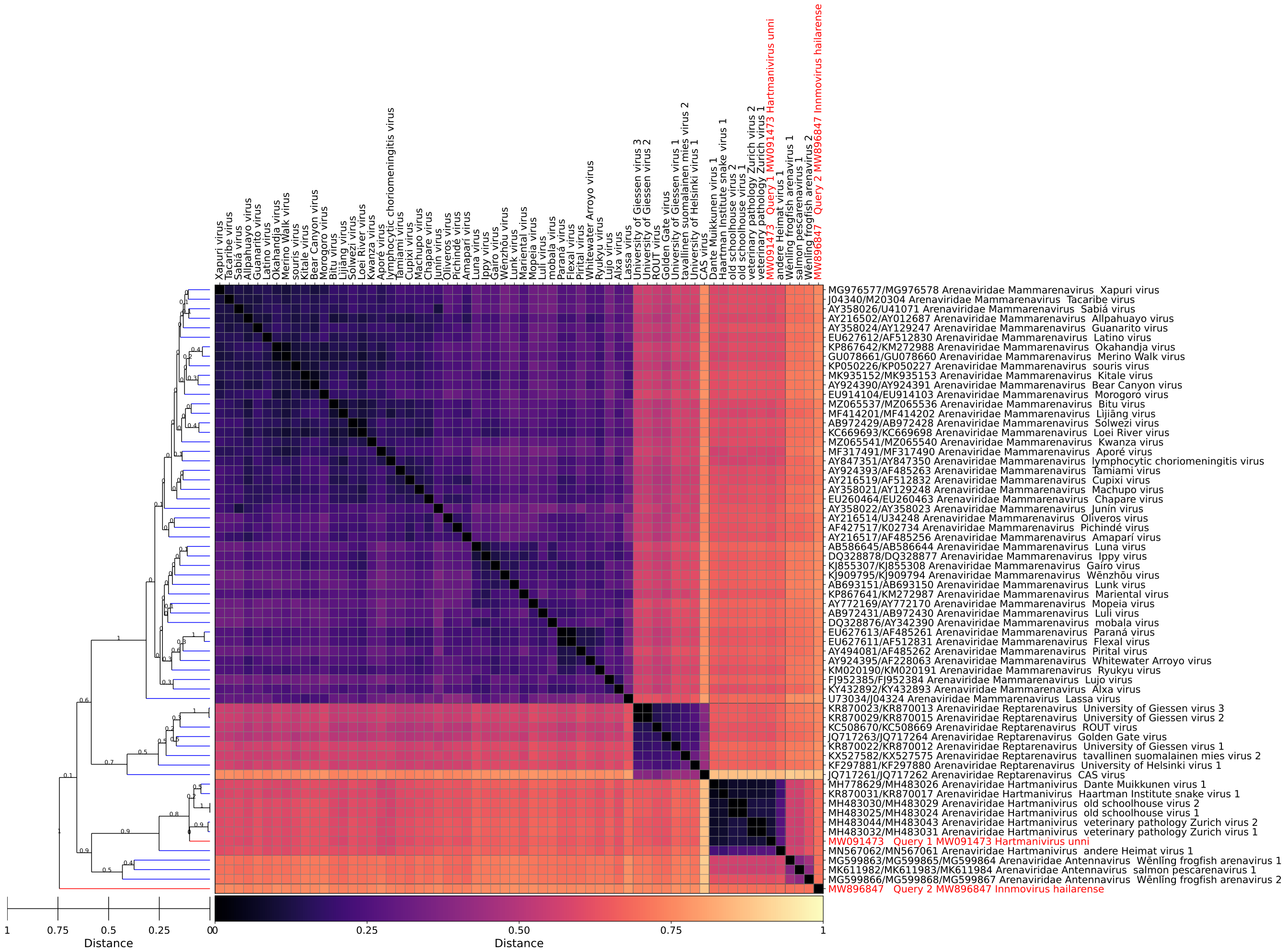

Figure 35: *Hartmanivirus*, GRAViTy-V2 heatmap

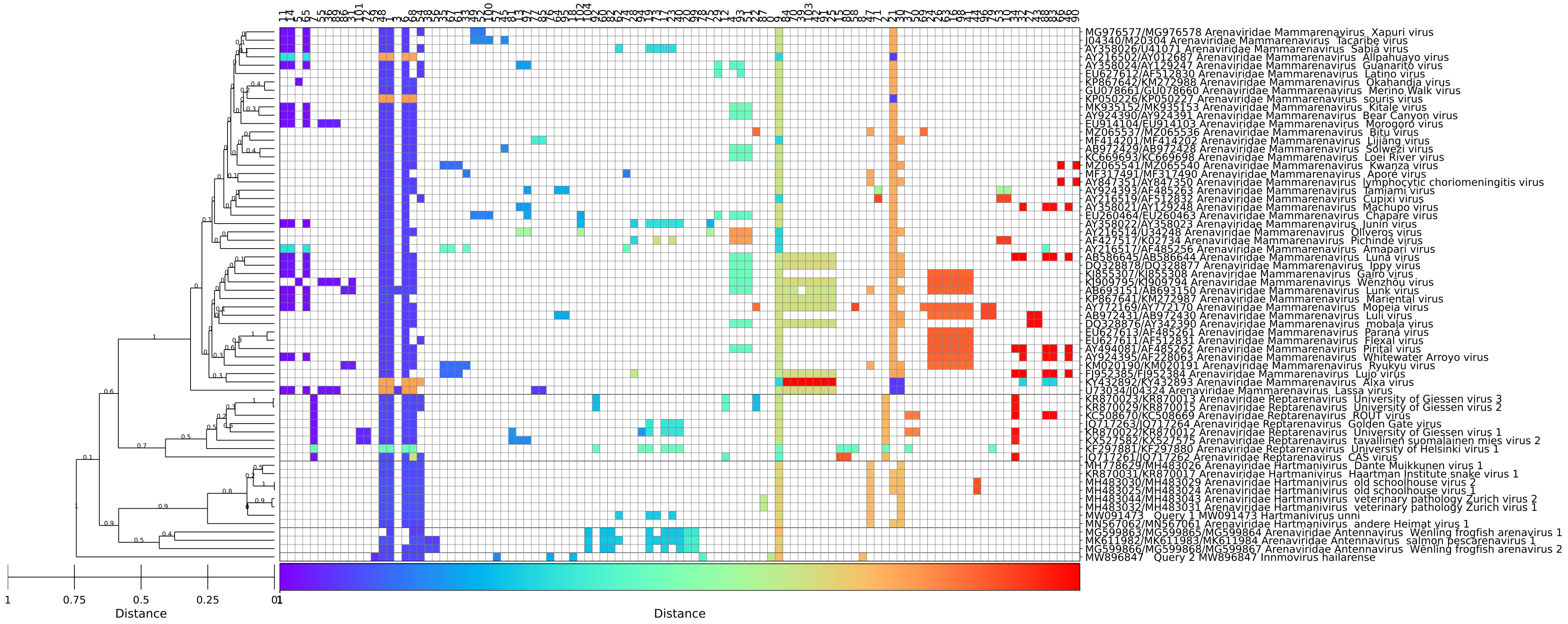

Figure 36: *Hartmanivirus*, GRAViTy-V2 barcode

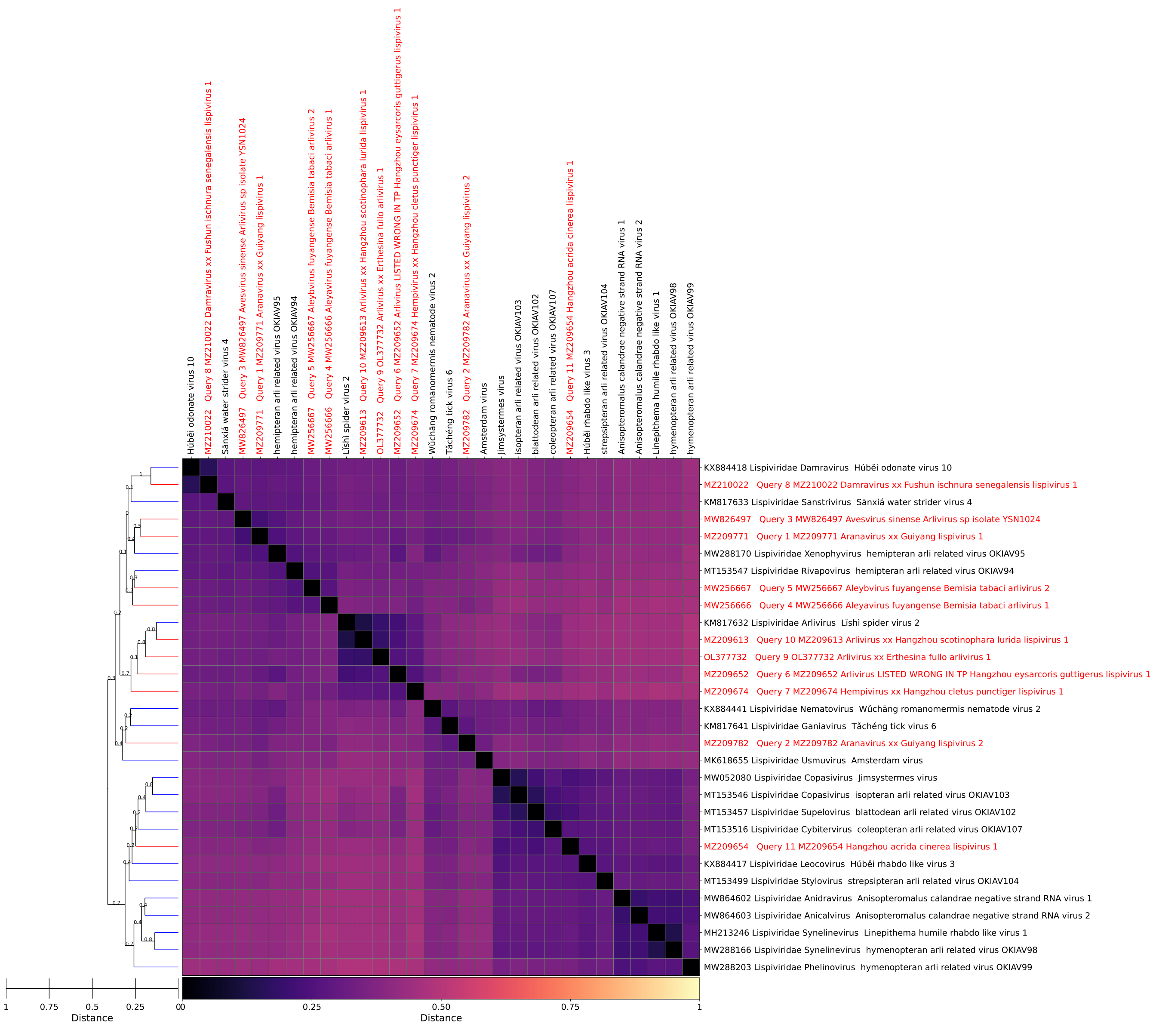

Figure 37: *Lispiviridae*, GRAViTy-V2 heatmap

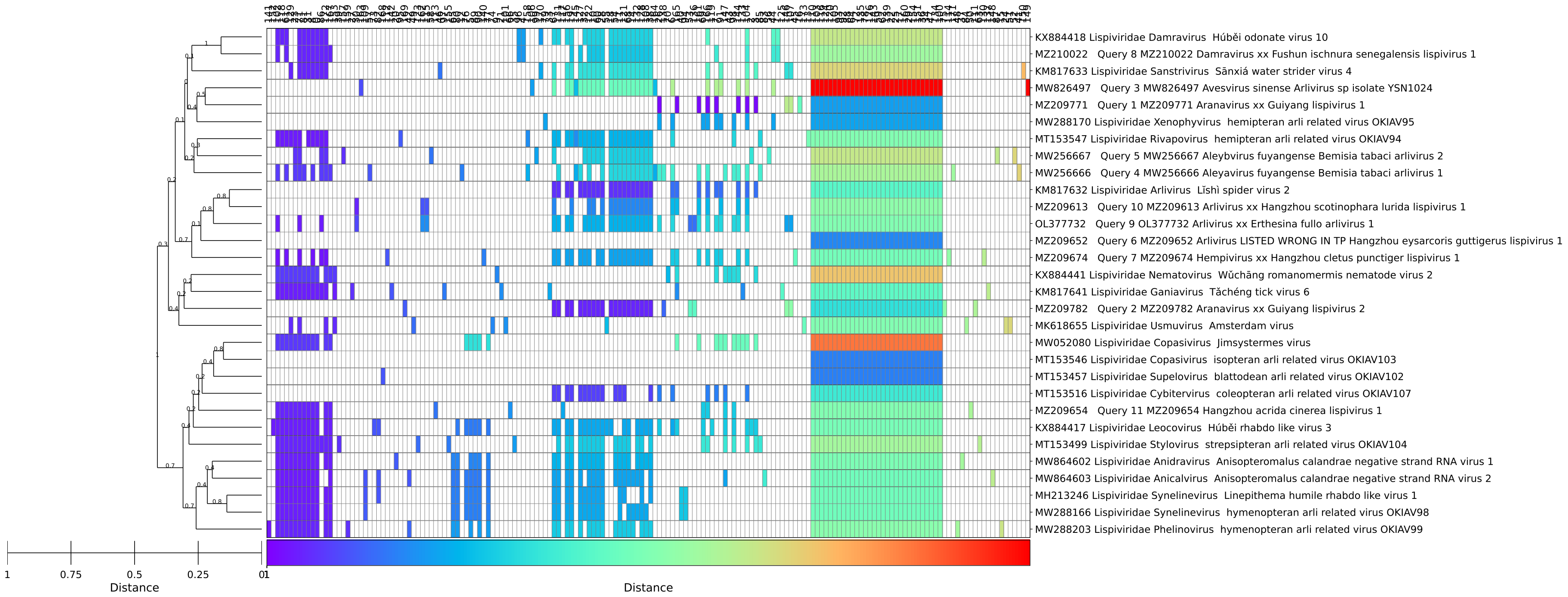

Figure 38: *Lispiviridae*, GRAViTy-V2 barcode

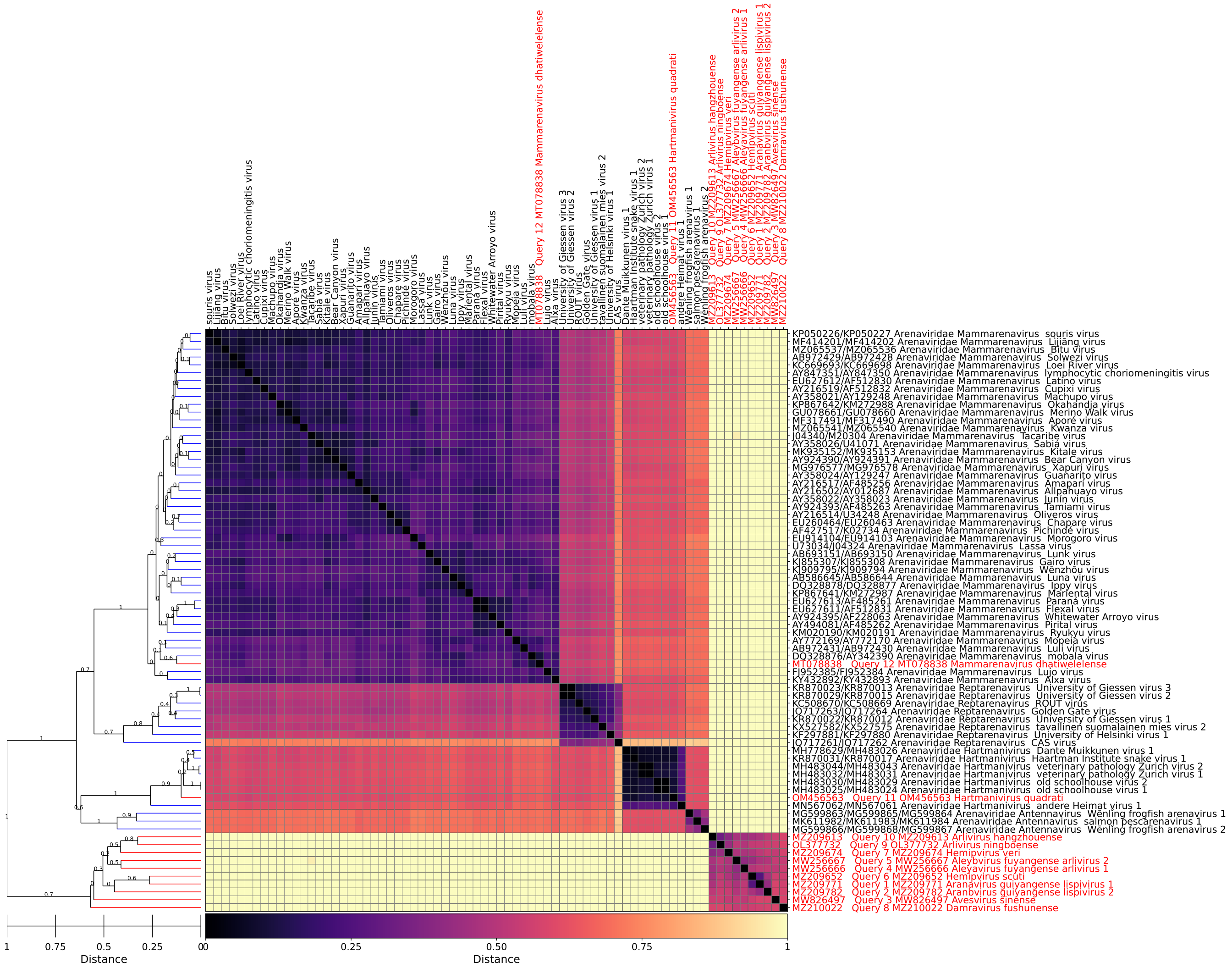

Figure 39: *Mammarenavirus*, GRAViTy-V2 heatmap

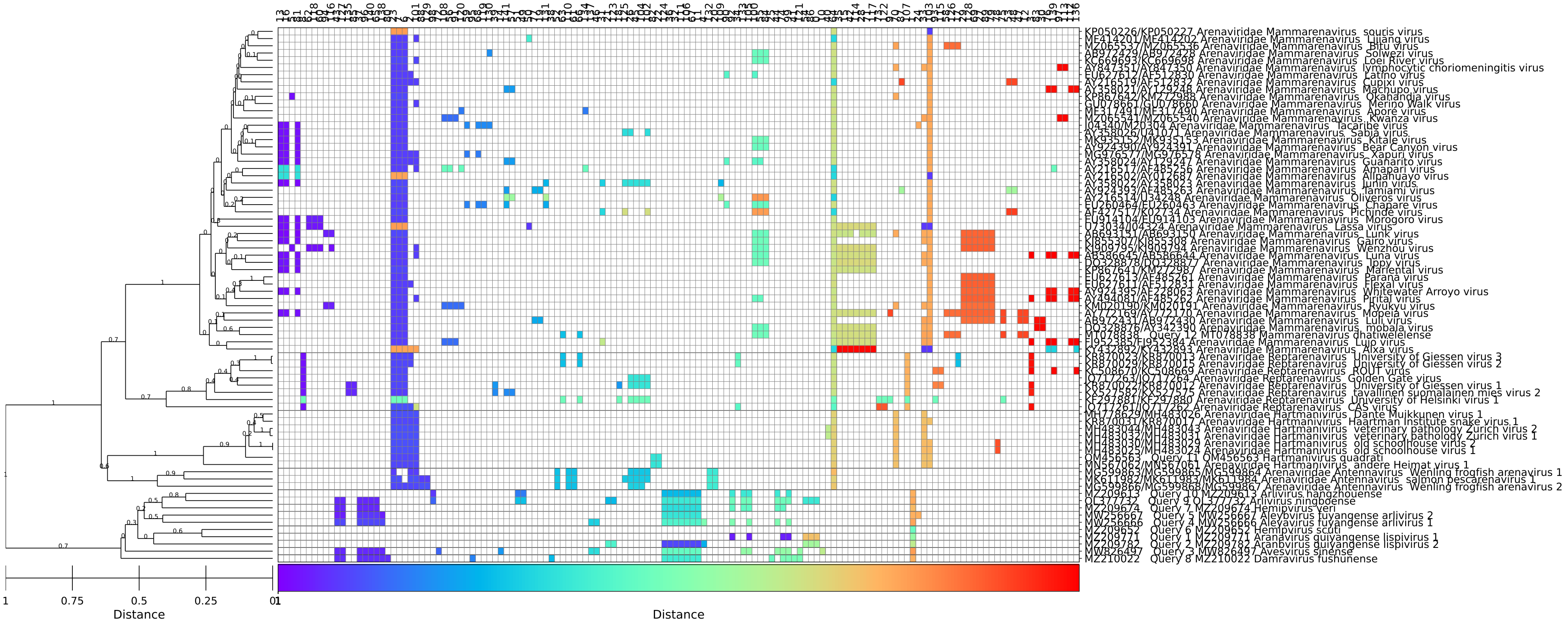

Figure 40: *Mammarenavirus*, GRAViTy-V2 barcode

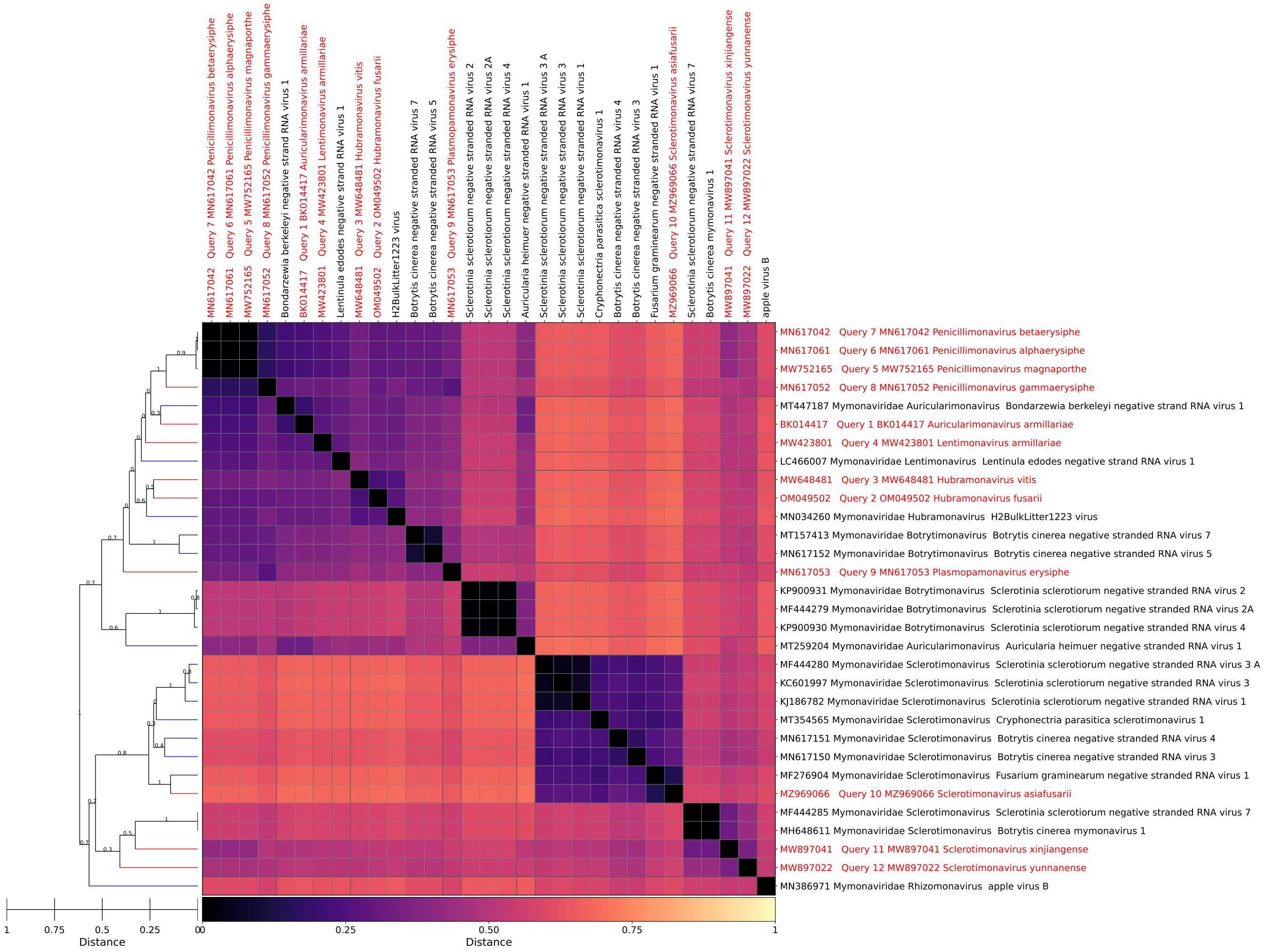

Figure 41: *Mymonaviridae*, GRAViTy-V2 heatmap

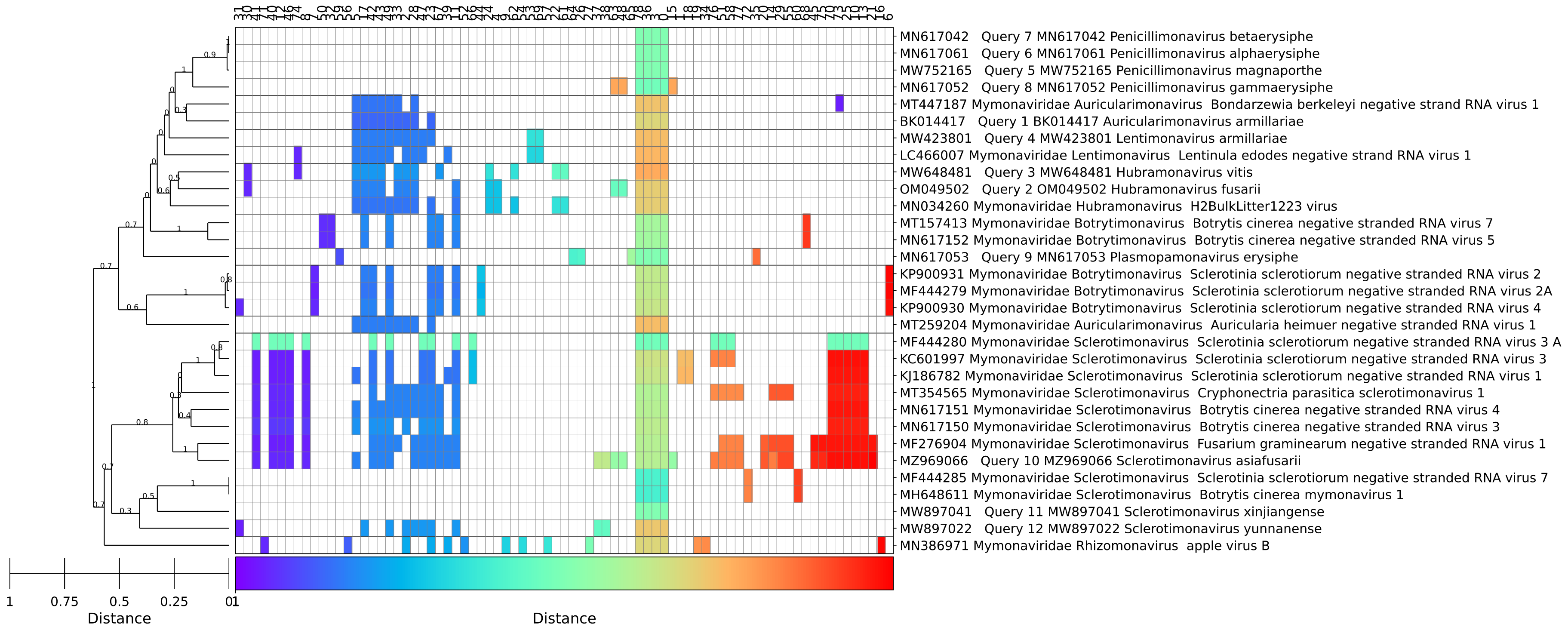

Figure 42: *Mymonaviridae*, GRAViTy-V2 barcode

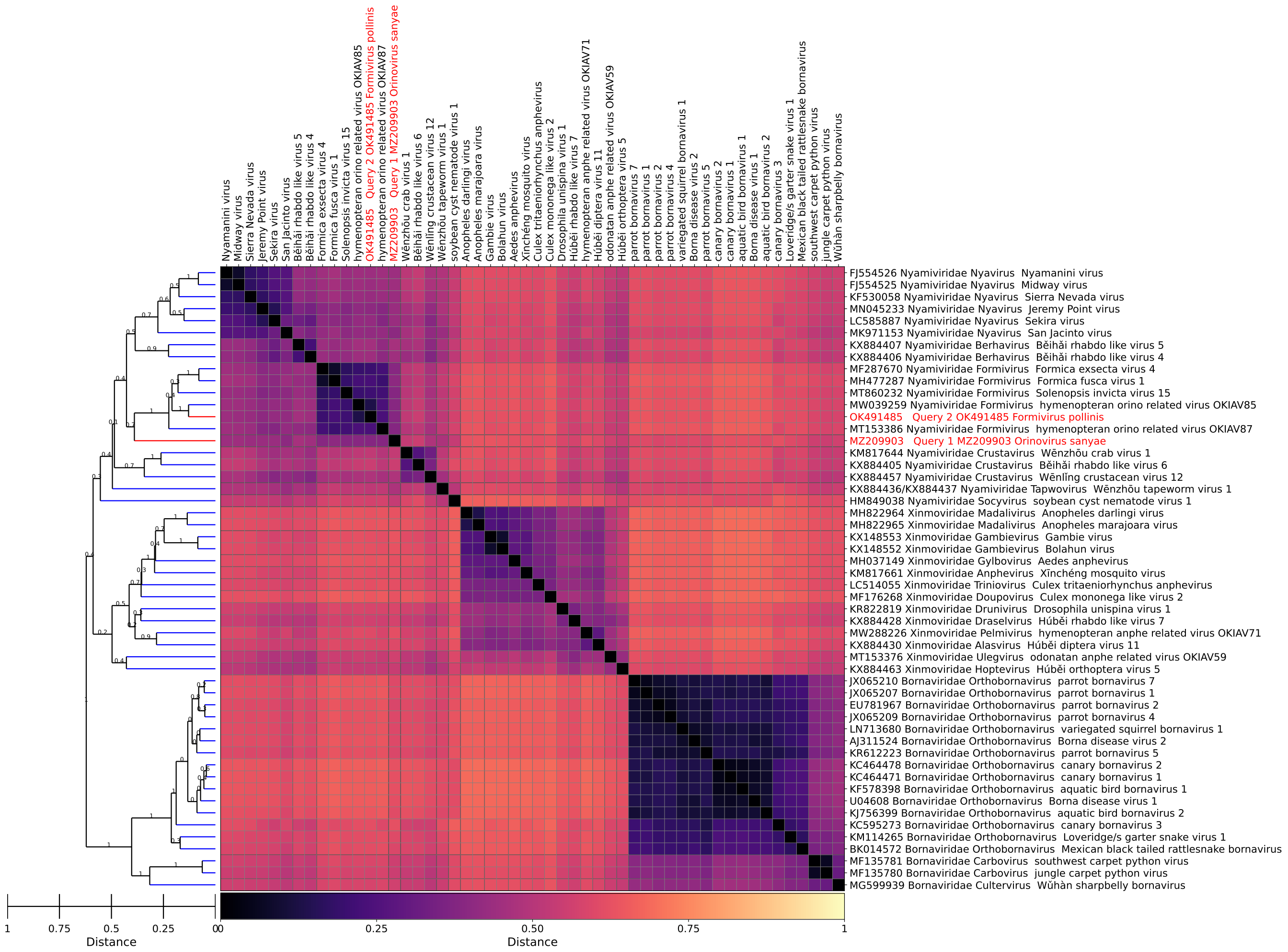

Figure 43: *Nyamiviridae*, GRAViTy-V2 heatmap

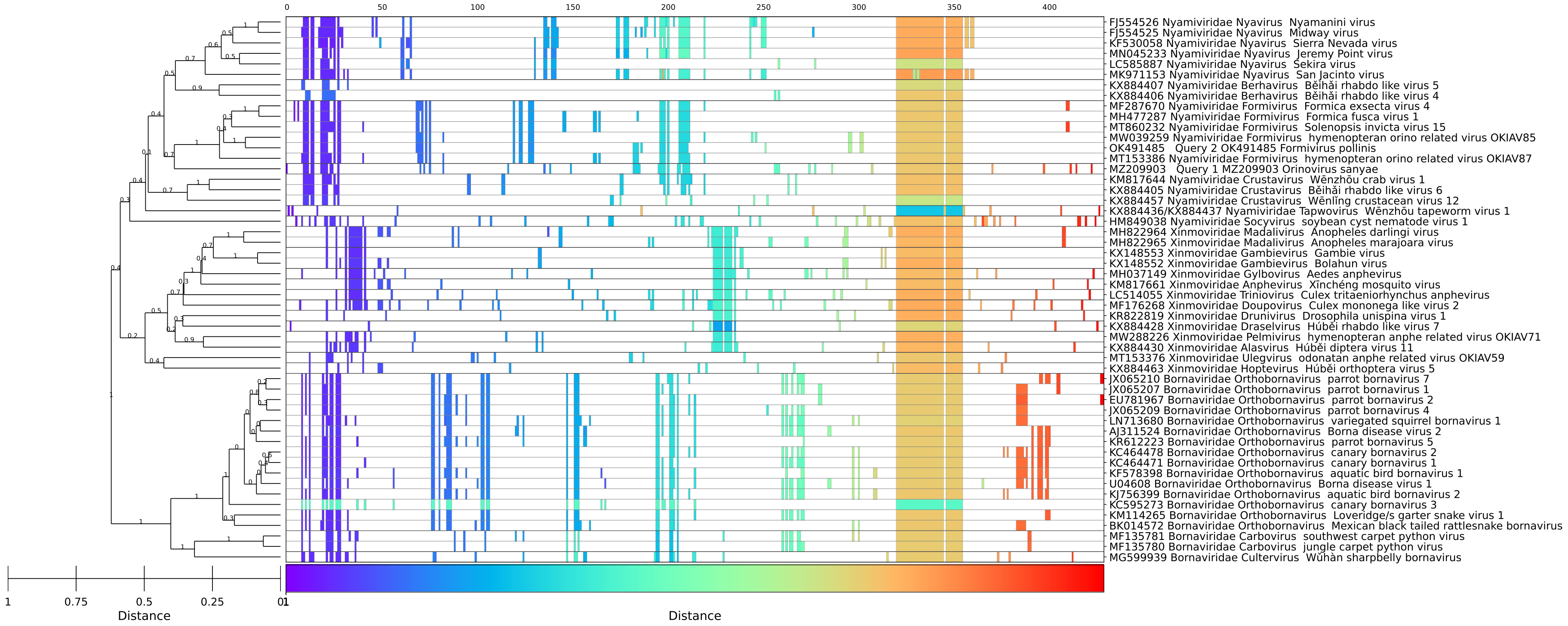

Figure 44: *Nyamiviridae*, GRAViTy-V2 barcode

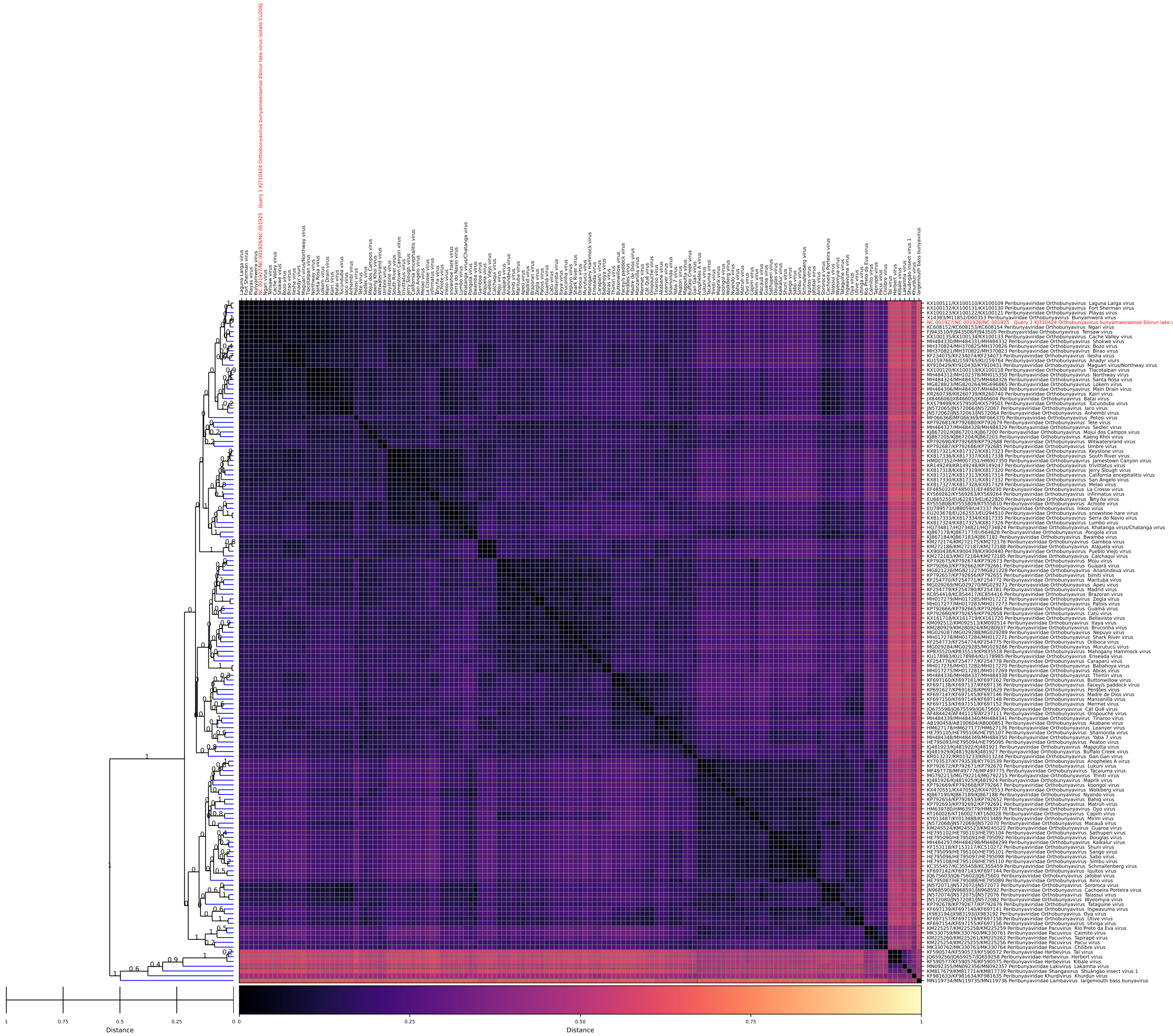

Figure 45: *Orthobunyavirus* (017M), GRAViTy-V2 heatmap

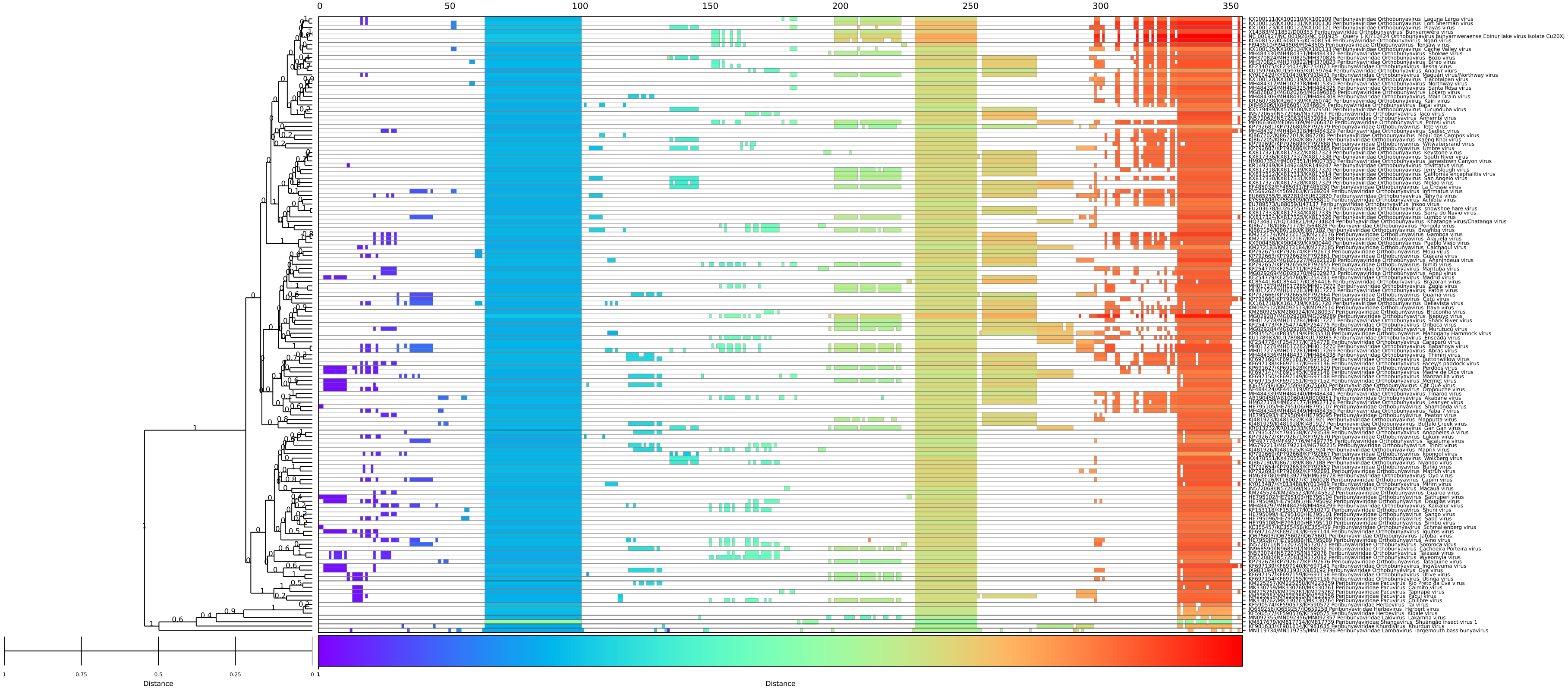

Figure 46: *Orthobunyavirus* (017M), GRAViTy-V2 barcode

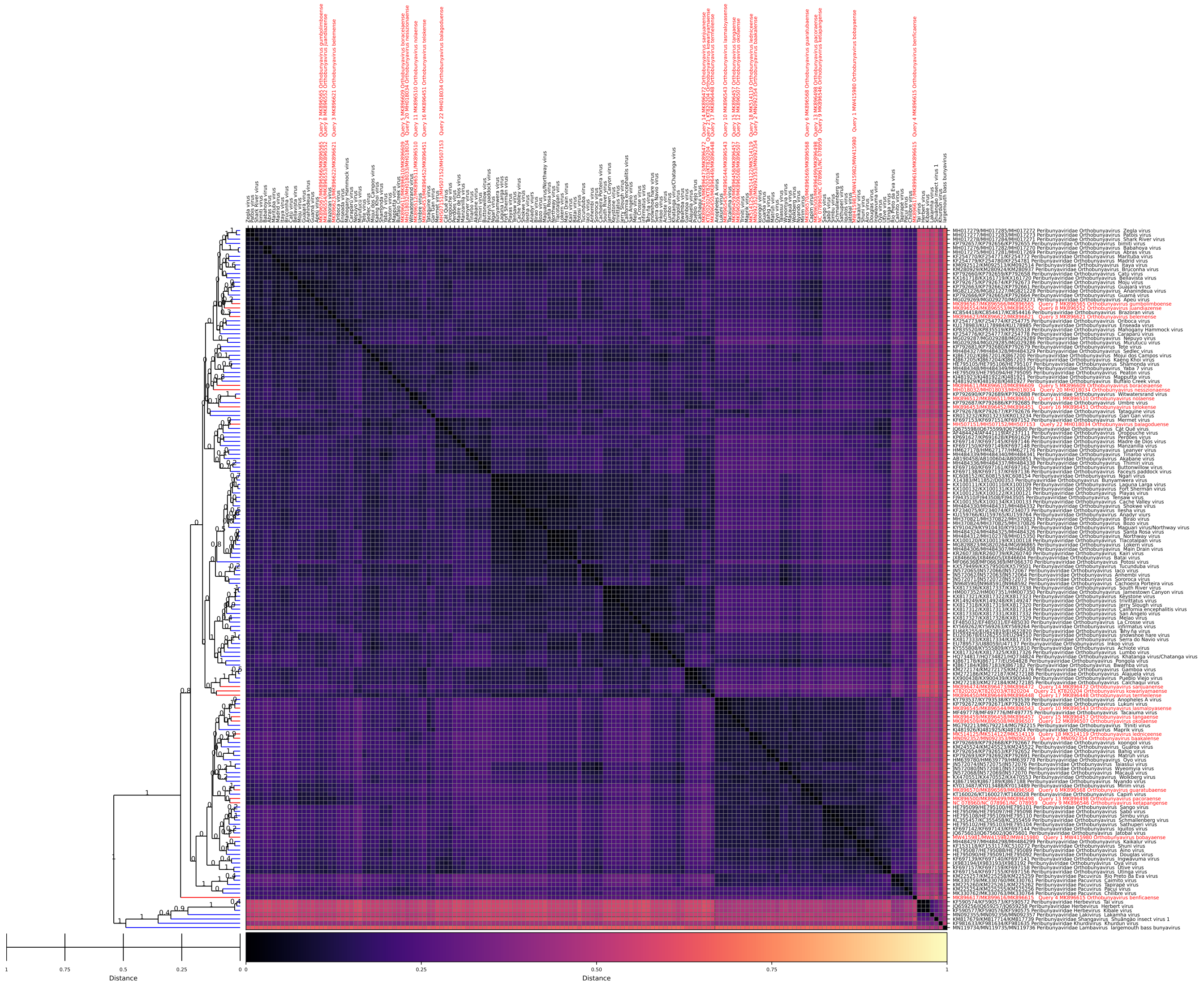

Figure 47: *Orthobunyavirus* (018M), GRAViTy-V2 heatmap

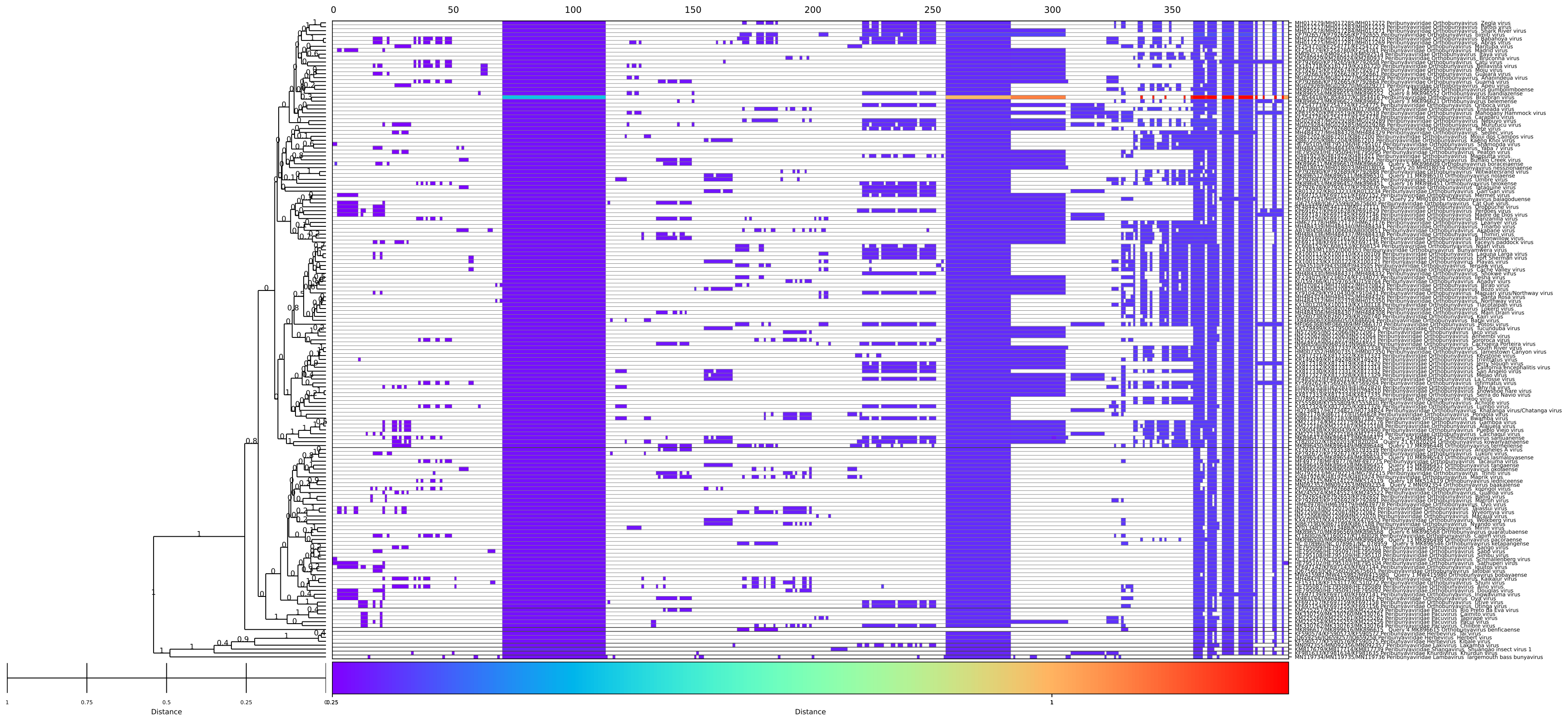

Figure 48: *Orthobunyavirus* (018M), GRAViTy-V2 barcode

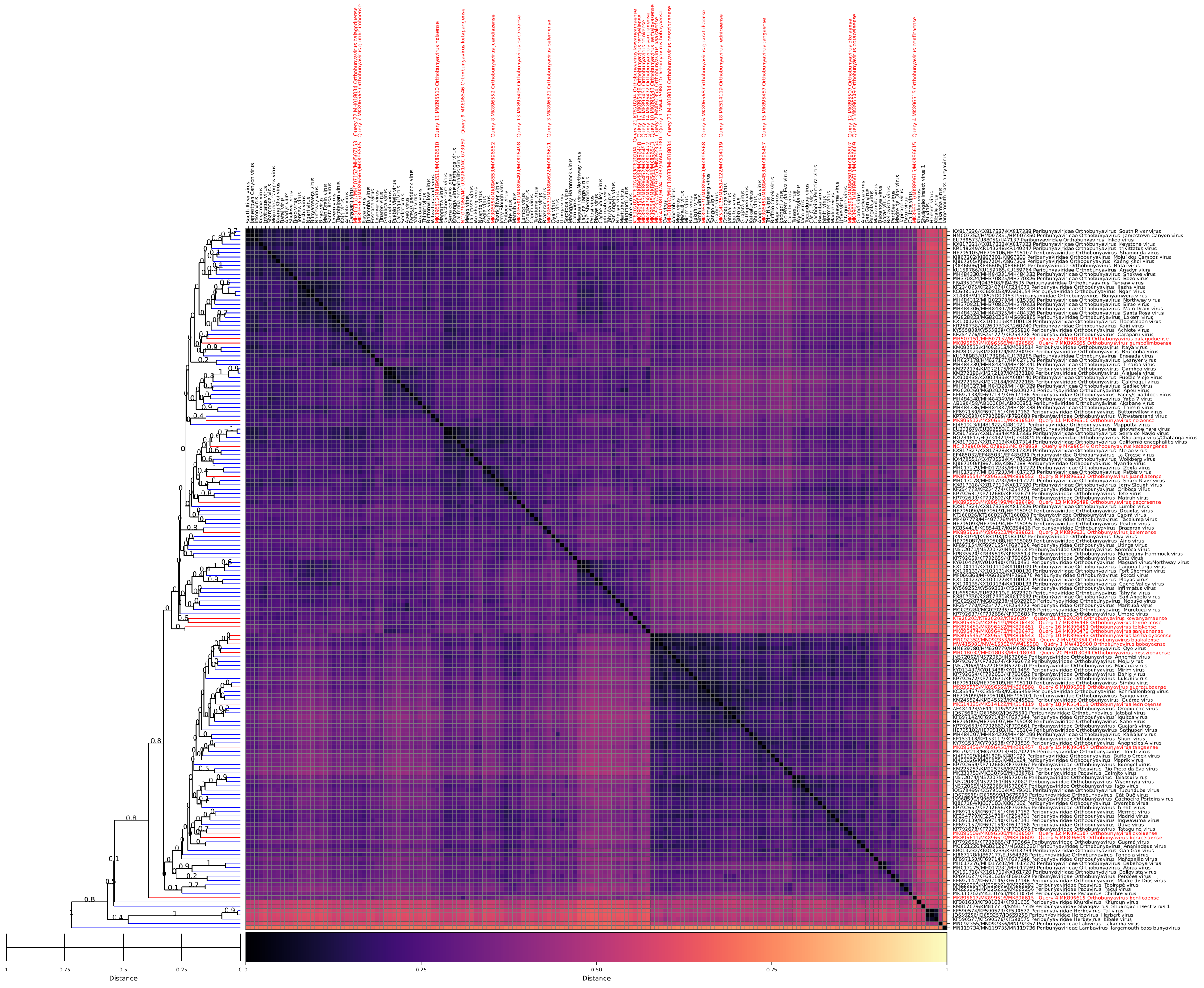

Figure 49: *Phasmaviridae*, GRAViTy-V2 heatmap

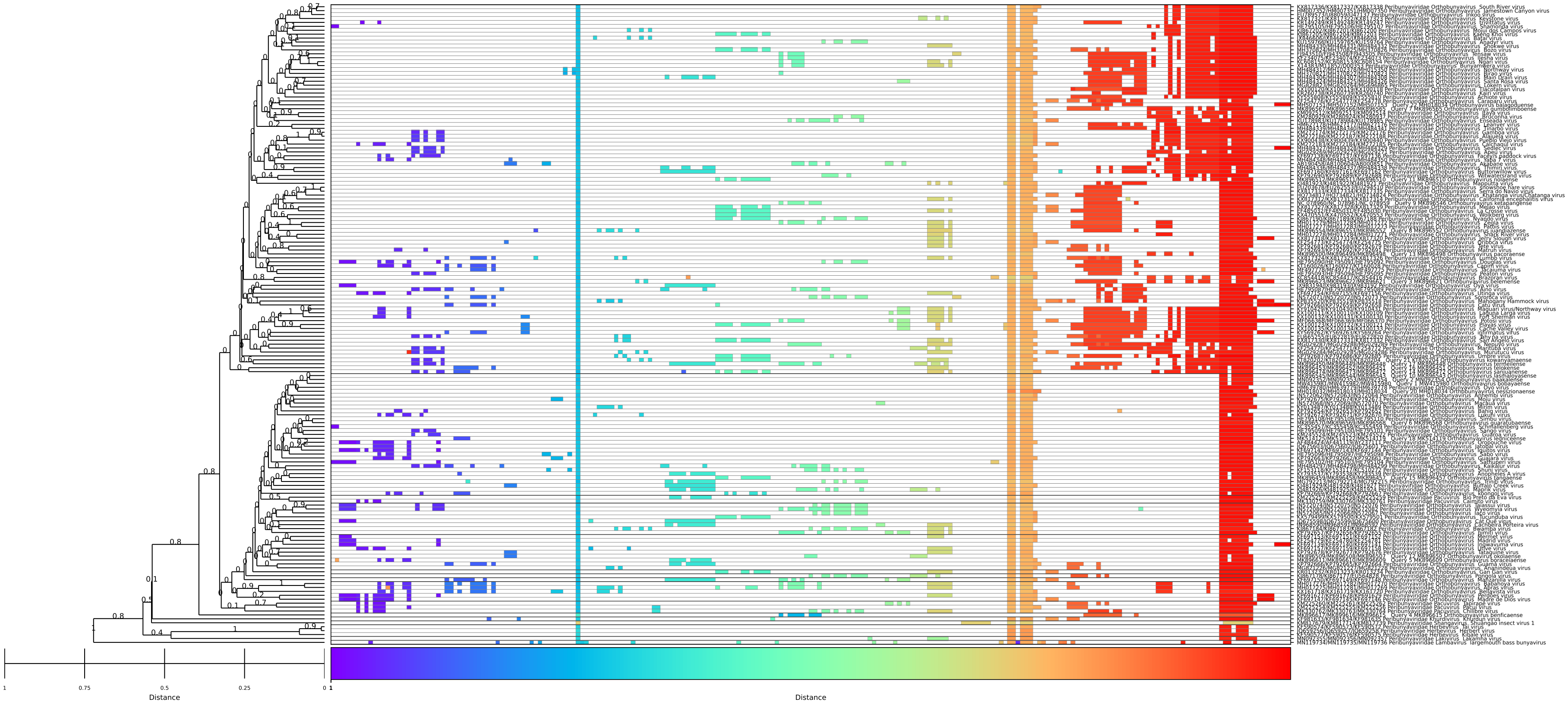

Figure 50: *Phasmaviridae*, GRAViTy-V2 barcode

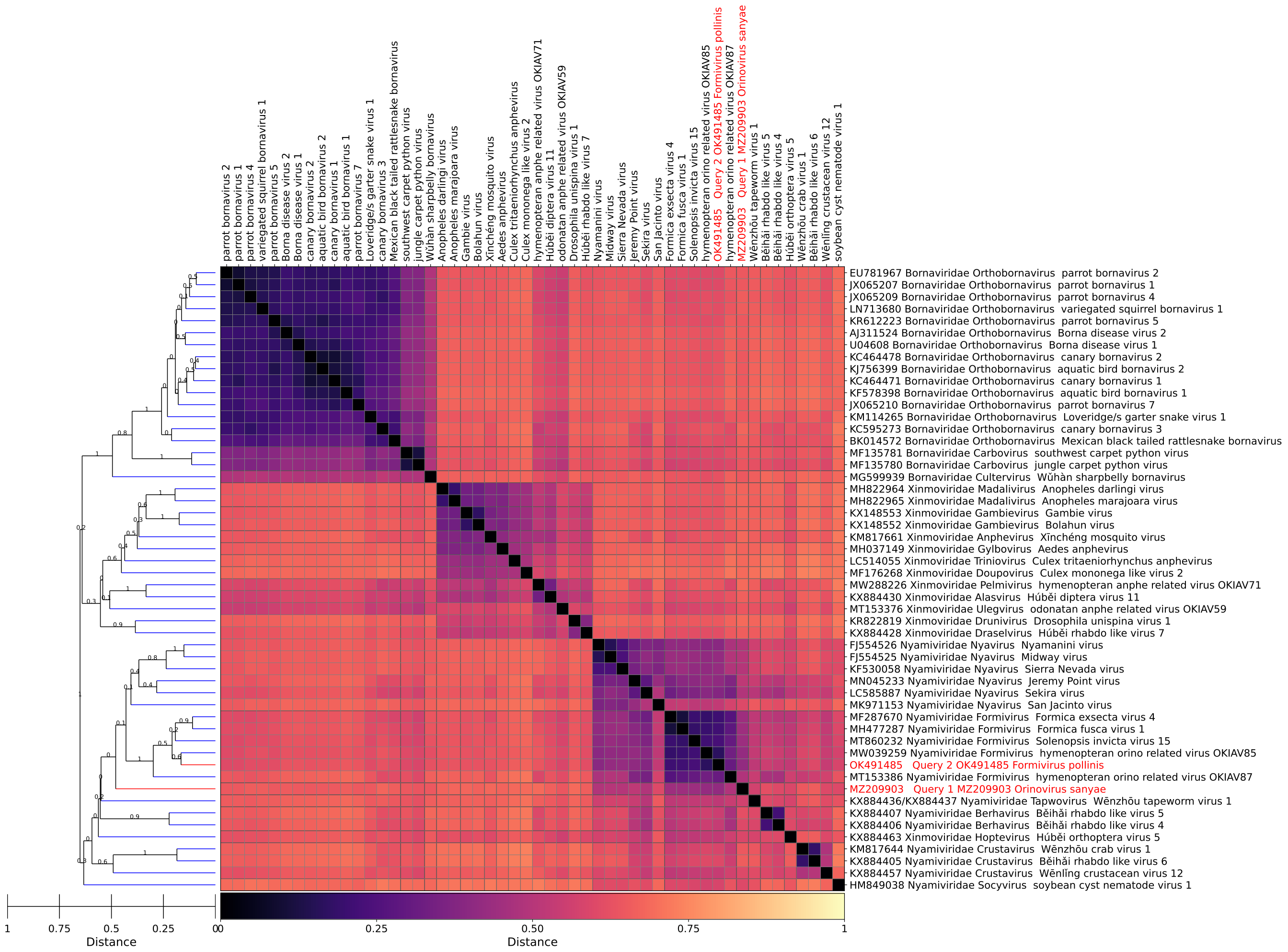

Figure 51: *Phenuiviridae*, GRAViTy-V2 heatmap

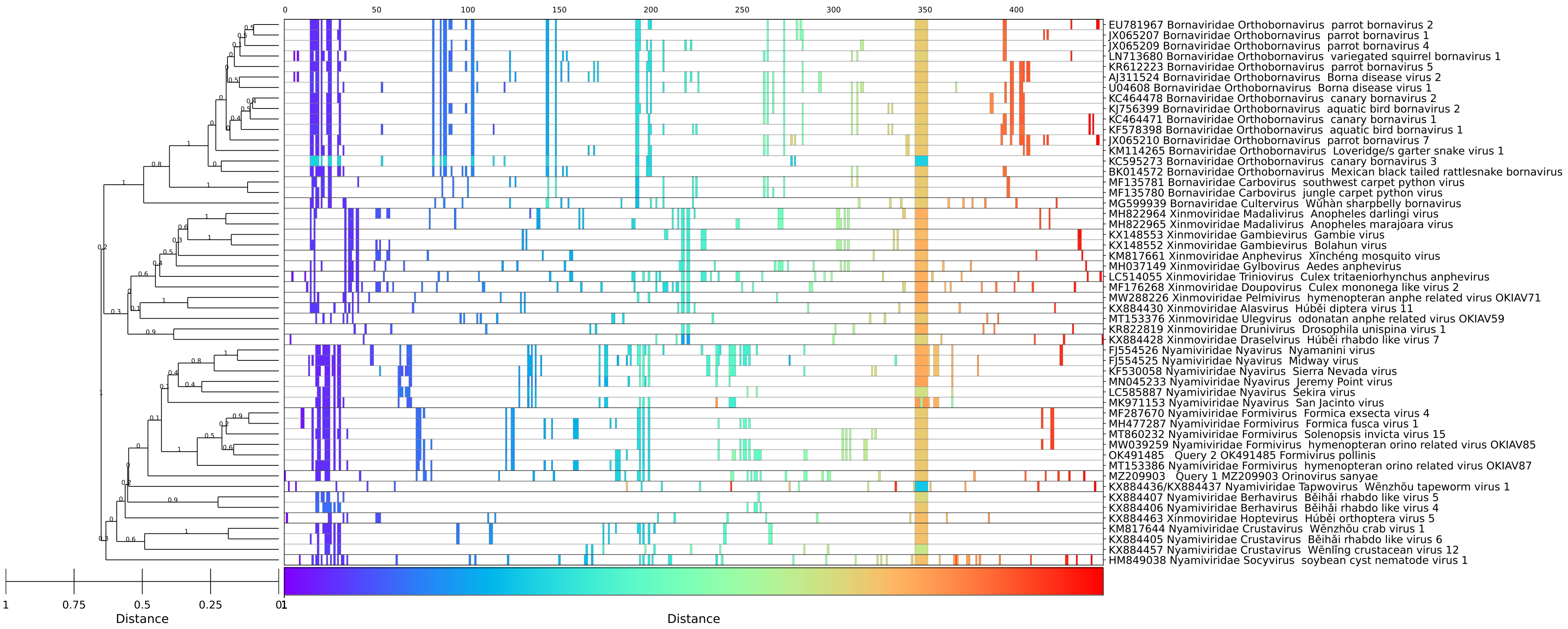

Figure 52: *Phenuiviridae*, GRAViTy-V2 barcode

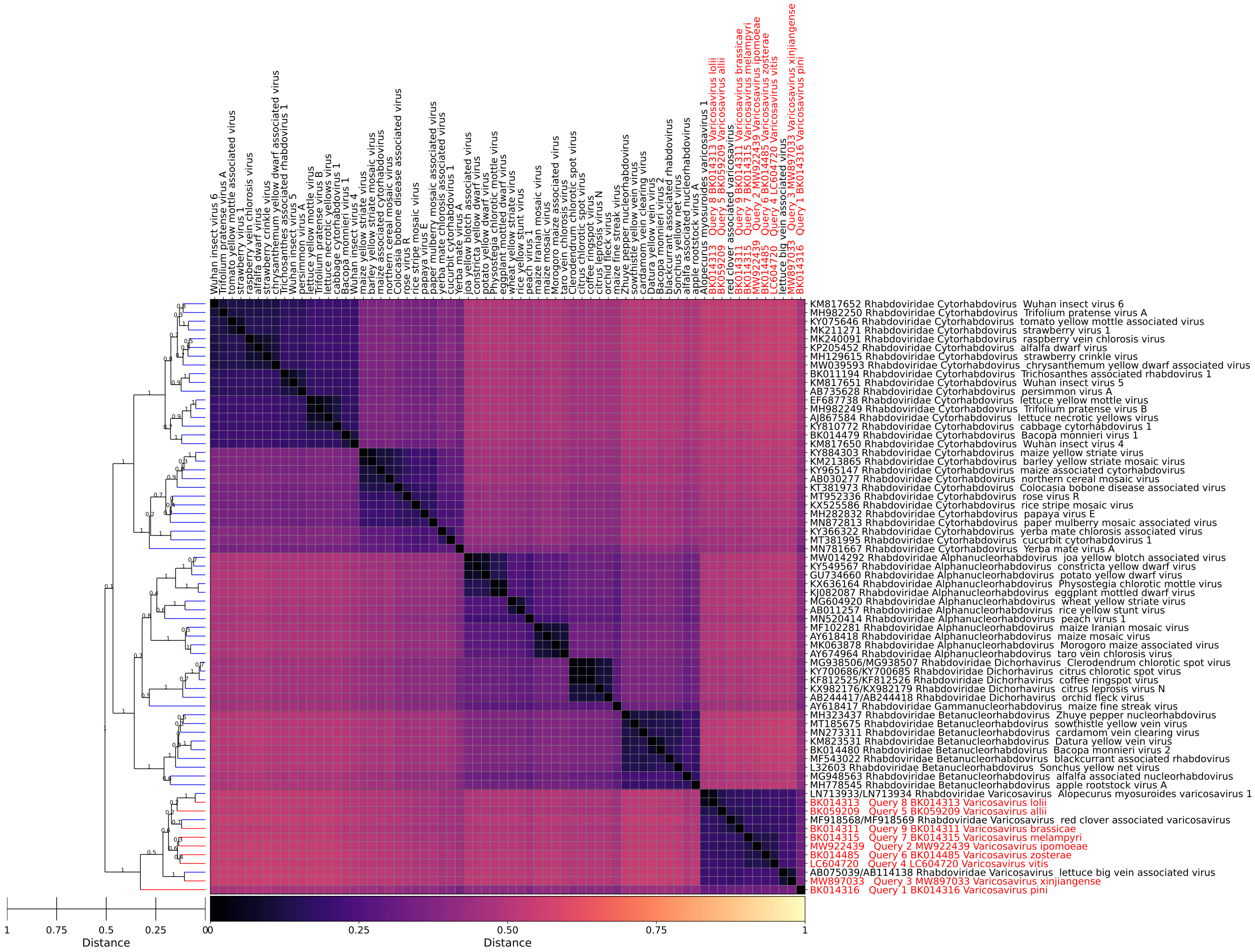

Figure 53: *Varicosavirus*, GRAViTy-V2 heatmap

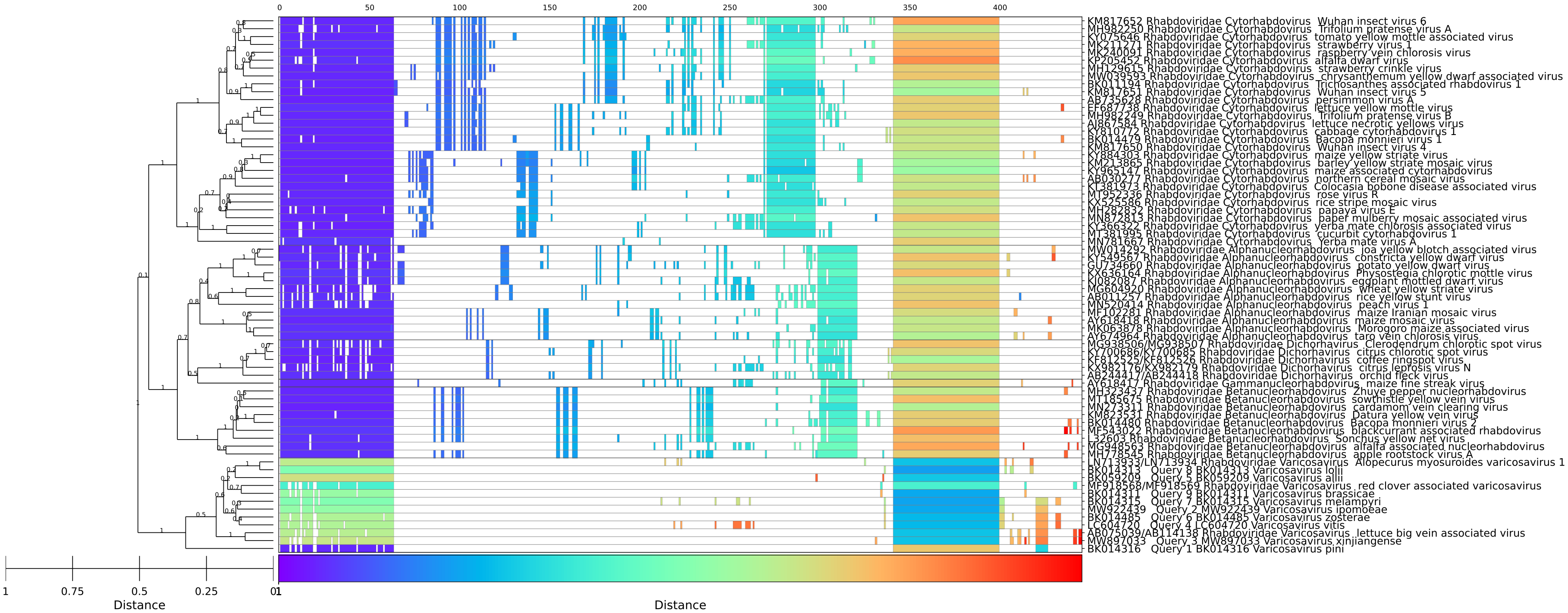

Figure 54: *Varicosavirus*, GRAViTy-V2 barcode

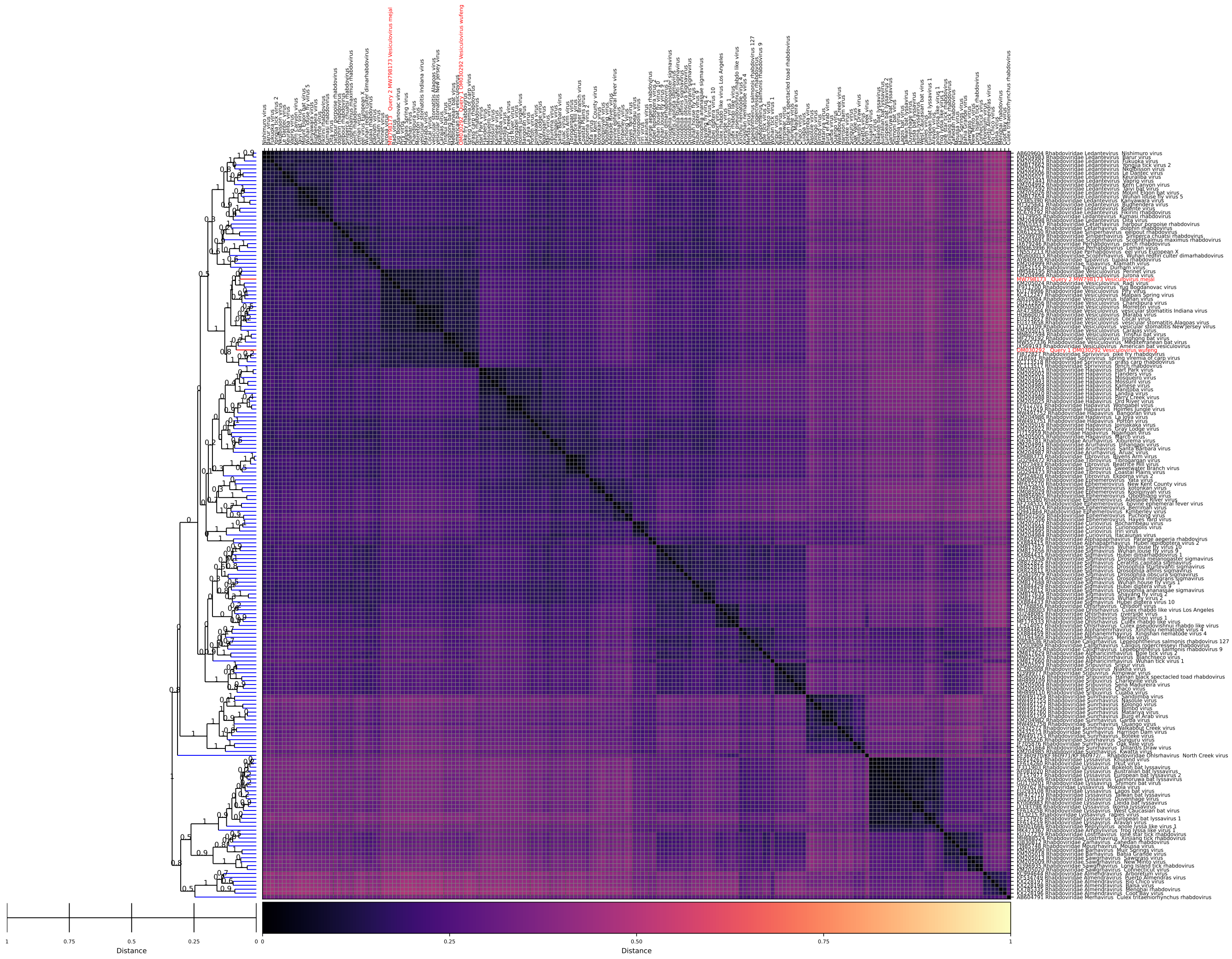

Figure 55: *Vesiculovirus*, GRAViTy-V2 heatmap

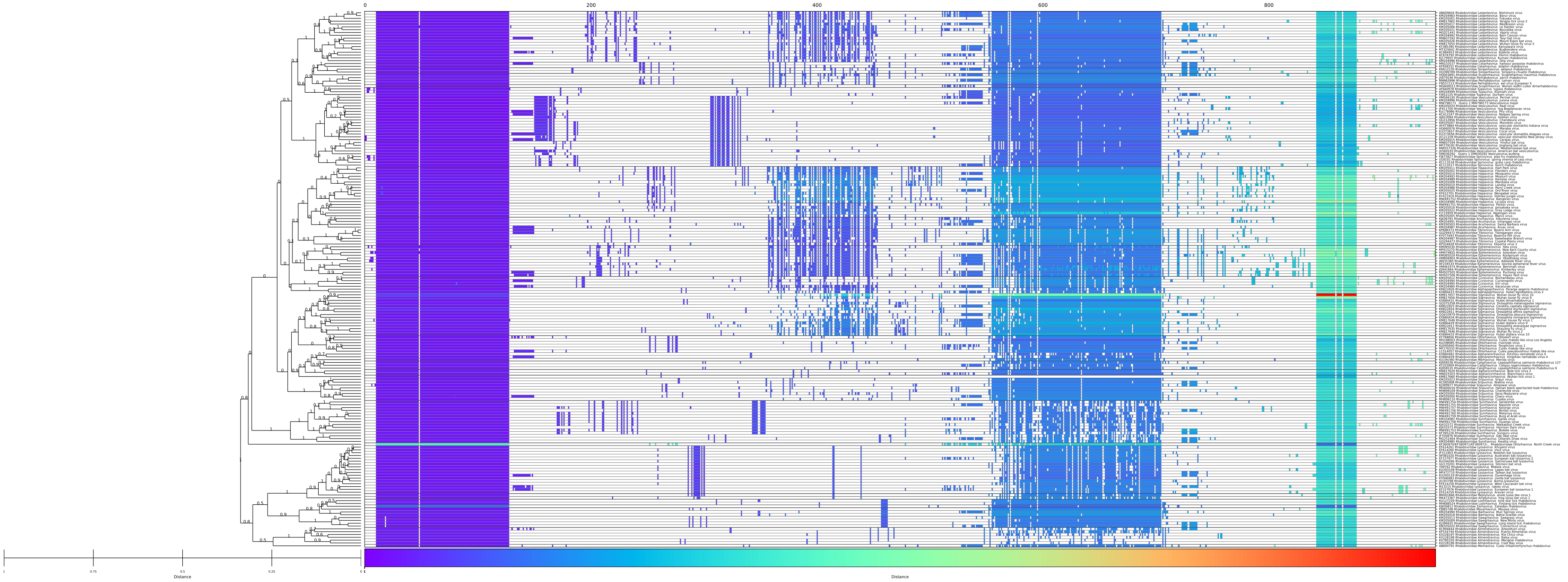

Figure 56: *Vesiculovirus*, GRAViTy-V2 barcode
