# Supplementary material for: GRAViTy-V2: a grounded viral taxonomy application
Source: NAR Genom Bioinform. 2024 Dec 18;6(4):lqae183. doi: 10.1093/nargab/lqae183 (PMC11655284; doi:10.1093/nargab/lqae183)
Supplement: lqae183_Supplemental_Files [file lqae183_supplemental_files.zip › GRAViTy_manuscript_SI_Document_3.pdf]

GRAViTy-V2: a grounded viral taxonomy application

Mayne, R., Aiewsakun, P., Turner, D., Adriaenssens, E. and Simmonds, P. (2024)

Supplementary information, Document 3

		GRAViTy-V2			GRAViTy			Comparison
TP	N	NFV	NGV	Run time	NFV	NGV	Run time	Time diff
Nyamiviridae	21	0	0	1.42	3	1	4.23	0.66
Jingchuvirales	58	5	2	1.93	6	2	4.01	0.52
Pestivirus	21	0	0	0.74	0	0	1.32	0.44
Hartmanivirus	64	0	0	1.8	0	0	3.96	0.55
Phasmaviridae	29	0	0	0.98	1	2	2.04	0.52
Varicosavirus	276	0	0	2.55	8	1	7.3	0.65
Imitervirales	22	0	0	150	-	-	-	
Herpesvirales	112	0	8	506	-	-	-	
Vesiculovirus	269	0	0	9.13	3	0	15.28	0.40
Alternaviridae	62	6	11	11	6	11	19.77	0.44

Table 1: GRAViTy-V2 vs original version run comparison. N = number of genomes in analysis; NFV = number of family violations; NGV = number of genus violations; time diff = difference in run time, 1 − (V2 run time ÷ original run time)

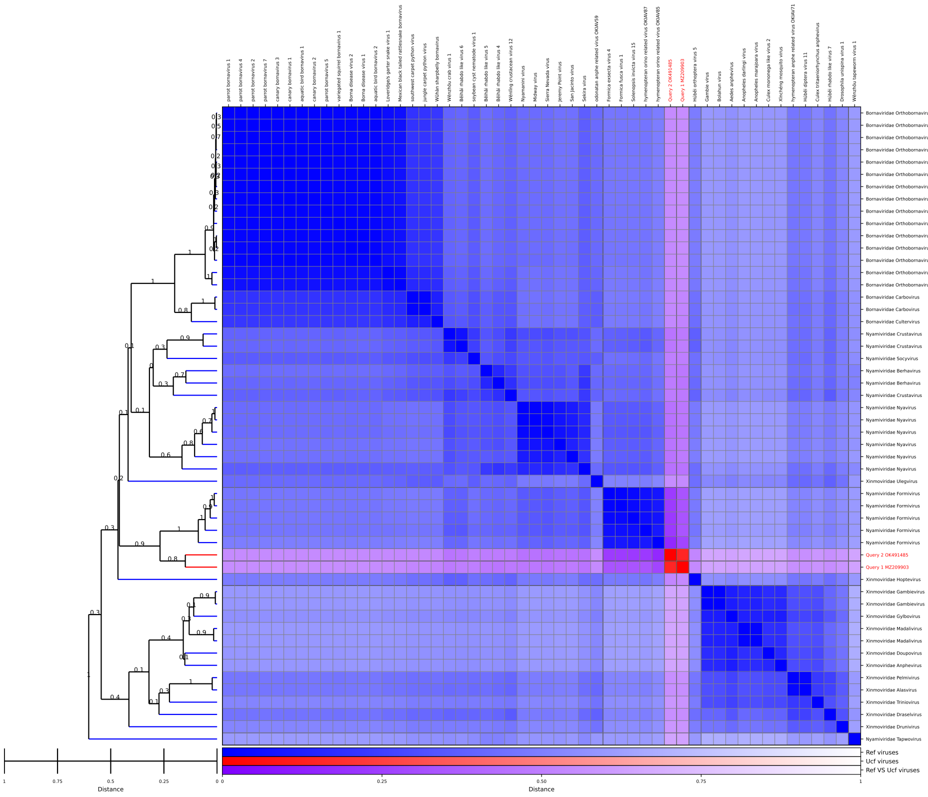

Figure 1: Analysis of *Nyamiviridae* TP with original GRAViTy.

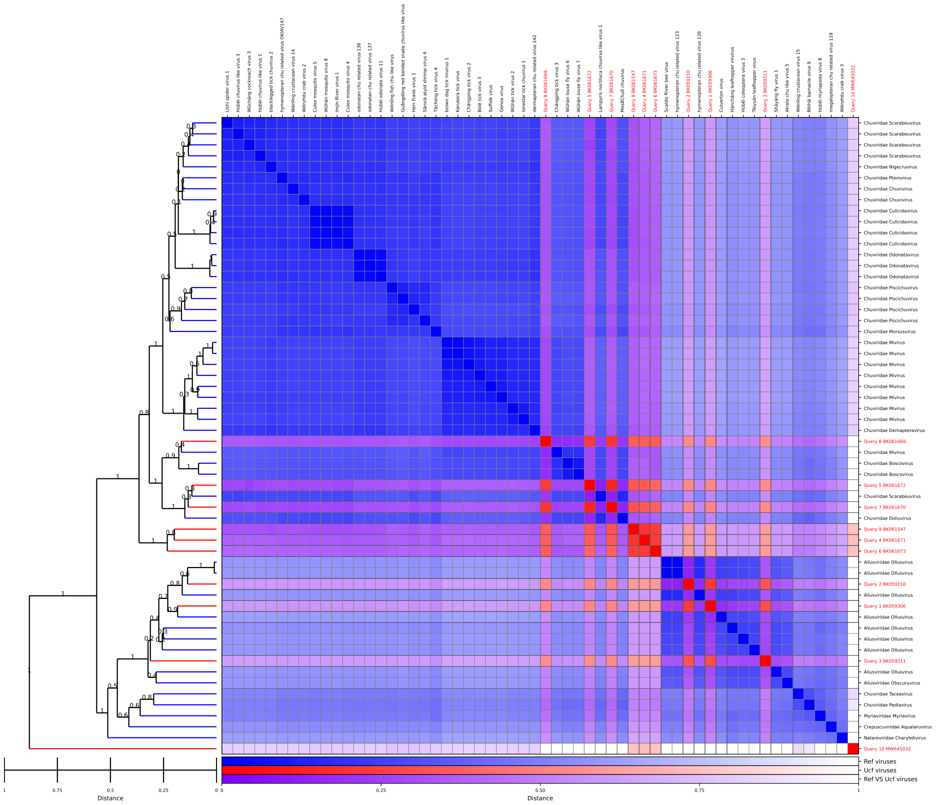

Figure 2: Analysis of *Jingchuvirales* TP with original GRAViTy.

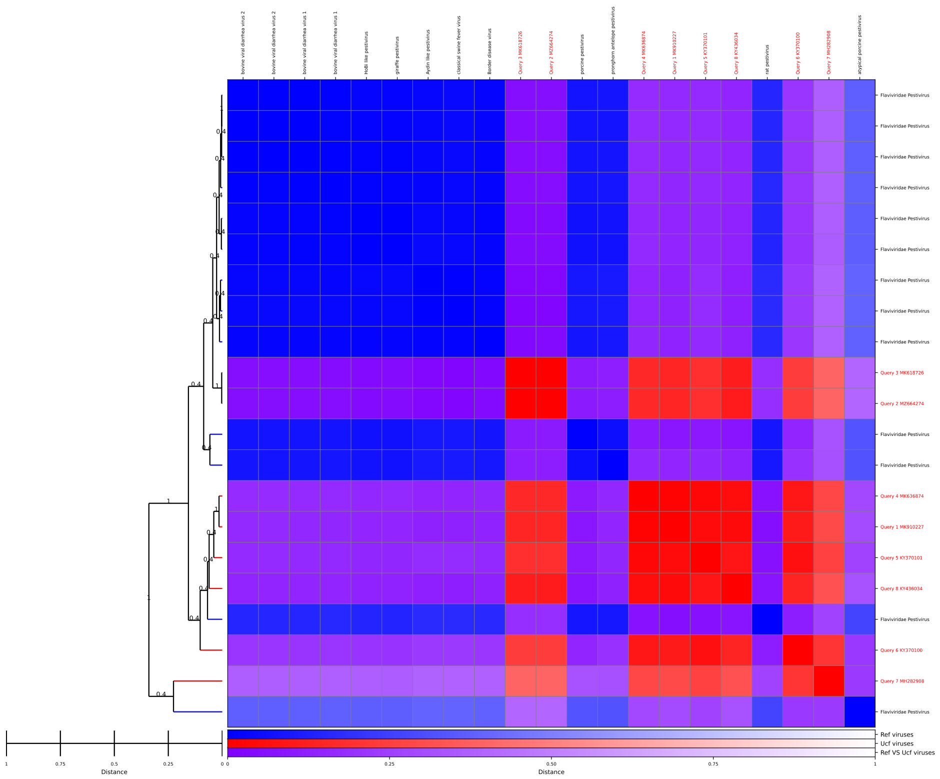

Figure 3: Analysis of *Pestivirus* TP with original GRAViTy.

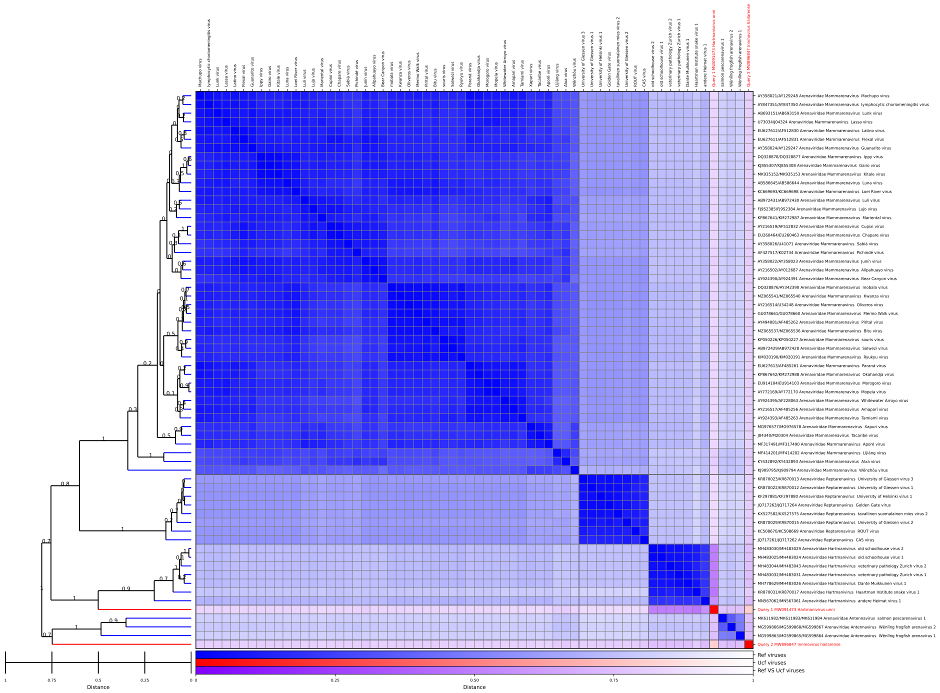

Figure 4: Analysis of *Hartmanivirus* TP with original GRAViTy.

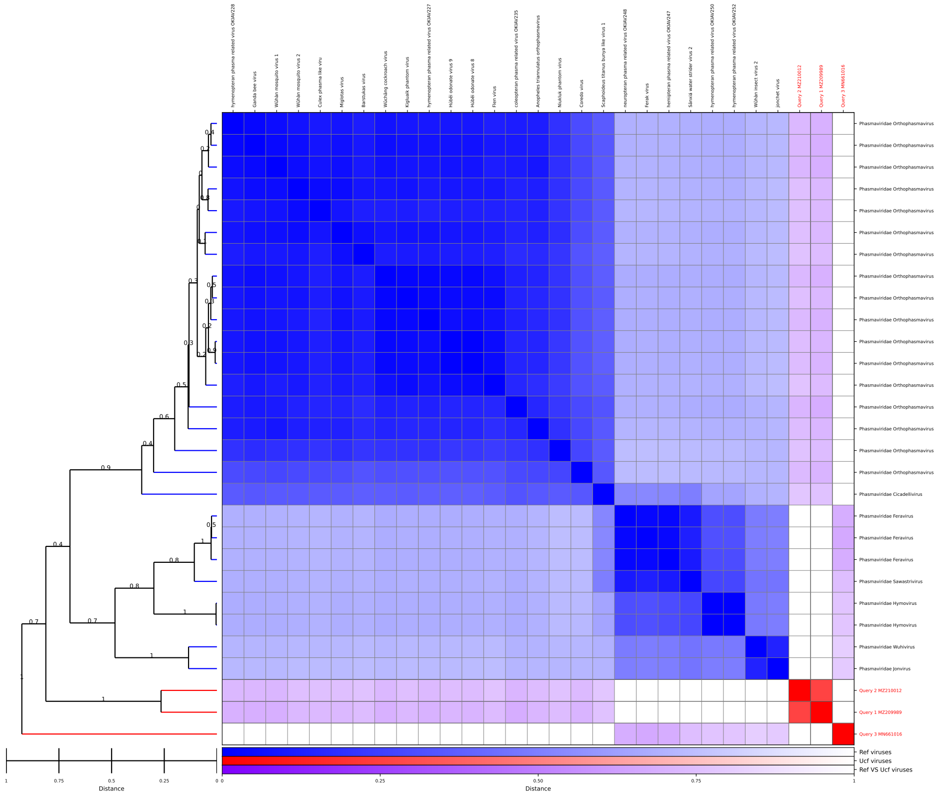

Figure 5: Analysis of *Phasmaviridae* TP with original GRAViTy.

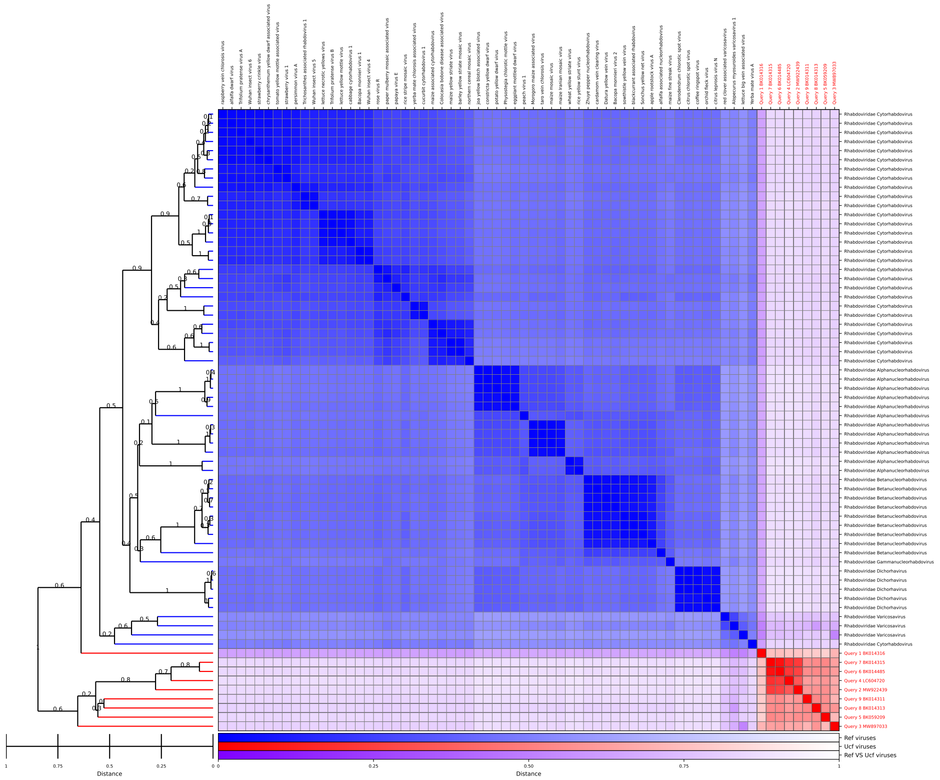

Figure 6: Analysis of *Varicosavirus* TP with original GRAViTy.

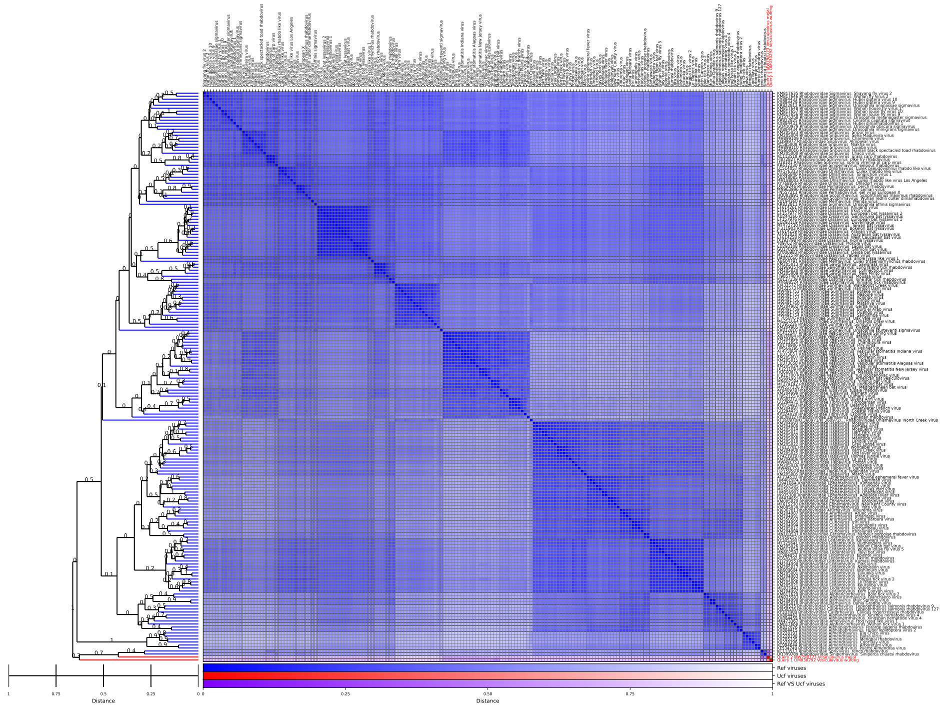

Figure 7: Analysis of *Vesiculovirus* TP with original GRAViTy.

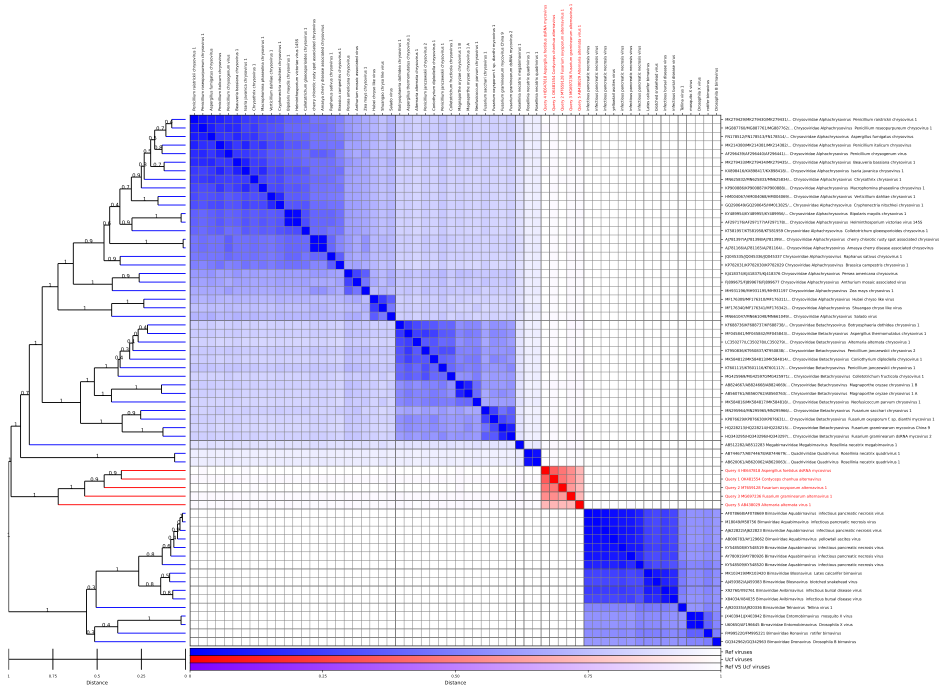

Figure 8: Analysis of *Alternaviridae* TP with original GRAViTy.
